# Inhibition of autophagy induced by tetrandrine promotes the accumulation of reactive oxygen species and sensitizes efficacy of tetrandrine in pancreatic cancer

**DOI:** 10.1186/s12935-024-03410-5

**Published:** 2024-07-10

**Authors:** Yiwei Wang, Ting Xu, Hongcheng Wang, Guanggai Xia, Xinyu Huang

**Affiliations:** Shanghai Sixth People’s Hospital Affiliated to Shanghai Jiao Tong University School of Medicine, 600 Yishan Rd, Shanghai, 200233 China

**Keywords:** Pancreatic cancer, Tetrandrine, Autophagy, Apoptosis, Reactive oxygen species

## Abstract

Pancreatic cancer, characterized by its poor prognosis, exhibits a marked resistance to conventional chemotherapy and immunotherapy, underscoring the urgent need for more effective treatment modalities. In light of this, the present study is designed to assess the potential antineoplastic efficacy of a combined regimen involving tetrandrine, a plant-derived alkaloid, and autophagy inhibitors in the context of pancreatic cancer. Electron microscopy and immunoblots showed that tetrandrine promoted the formation of autophagosomes and the upregulation of LC3II and the downregulation of p62 expression, indicating that tetrandrine induced autophagy in pancreatic cancer cells. Western blot revealed that tetrandrine inhibited the phosphorylation of AKT and mTOR, as well as the expression of Bcl-2, while upregulating Beclin-1 expression. Moreover, tetrandrine promoted the transcription and protein expression of ATG7. Following the combination of autophagy inhibitors and tetrandrine, the apoptotic rate and cell death significantly increased in pancreatic cancer cells. Consistent results were obtained when ATG7 was silenced. Additionally, tetrandrine induced the generation of ROS, which was involved in the activation of autophagy and apoptosis. Further investigation revealed that upon autophagy inhibition, ROS accumulated in pancreatic cancer cells, resulting in decreased mitochondrial membrane potential and further induction of apoptosis. The results of treating subcutaneous xenograft tumors with a combination of tetrandrine and chloroquine validated that autophagy inhibition enhances the toxicity of tetrandrine against pancreatic cancer in vivo. Altogether, our study demonstrates that tetrandrine induces cytoprotective autophagy in pancreatic cancer cells. Inhibiting tetrandrine-induced autophagy promotes the accumulation of ROS and enhances its toxicity against pancreatic cancer.

**Supplementary Information:**

The online version contains supplementary material available at 10.1186/s12935-024-03410-5.

## Introduction

Pancreatic cancer, one of the most aggressive malignancies, exhibits a 5-year survival rate of less than 10% for patients [1]. Early diagnosis of this disease poses challenges, and the majority of patients are unable to undergo curative surgery, leaving drug therapy as their only option [2, 3]. While clinical chemotherapy regimens, including single-agent and combination therapies, have demonstrated some efficacy against pancreatic cancer, they also come with significant side effects [4, 5]. Despite progress in research on chemotherapy drugs for pancreatic cancer, drug tolerance remains a limiting factor in the prognosis of patients [6]. Therefore, a systematic treatment approach is imperative for pancreatic cancer.

Macroautophagy (herein referred to as autophagy), an ancient and highly conserved cellular process, involves the formation of vesicles within cells to encapsulate proteins and organelles and the vesicles eventually fuse with lysosomes, forming autolysosomes that degrade their contents [7]. In normal cells, autophagy plays a role in cellular homeostasis by aiding in the repair and renewal of cells, as well as helping them adapt to stress such as hypoxia and starvation [8]. However, in tumor cells, autophagy exhibits a dual role under different conditions [9, 10]. Recent studies have shown that in pancreatic cancer, autophagy promotes and protects cancer cells and is closely associated with chemotherapy resistance [11, 12]. In vitro experiments indicate that autophagy plays a crucial role in the metabolism of pancreatic cancer cells. Targeted inhibition of autophagy may induce metabolic disruptions in PDAC, thereby impeding pancreatic cancer progression [13]. Clinical studies demonstrated that the autophagy inhibitor hydroxychloroquine (HCQ), when used in combination with gemcitabine, produced no adverse reactions and led to a significant decrease in CA19-9 levels in 61% of patients with pancreatic ductal adenocarcinoma (PDAC), with more than half of the patients benefiting in terms of survival [14]. Consequently, the strategy of enhancing chemotherapy drug sensitivity by inhibiting autophagy holds great potential for the treatment of pancreatic cancer.

*Stephania tetrandra S. Moore* is an herb whose root has been widely used in the treatment of asthma, hyperglycemia, malaria, fever, and tumors [15, 16]. Tetrandrine (TET), extracted from *Stephania tetrandra S. Moore*, is a bisbenzylisoquinoline alkaloid. In recent years, studies have demonstrated the inhibitory effects of TET on various tumors in vitro and in vivo [17]. TET inhibits the proliferation of hepatocellular carcinoma by suppressing CDK4 and CDK2-CyE [18]. Additionally, previous studies showed that TET induced apoptosis by activating the caspase pathway in various cancer [19, 20]. Moreover, TET was found to induce autophagy in liver cancer and oral cancer [21, 22]. However, limited research has been conducted on the antineoplastic effects of TET in pancreatic cancer. Here, we demonstrated that TET simultaneously induces autophagy and apoptosis in pancreatic cancer, and we found that TET-induced autophagy has a protective effect on pancreatic cancer cells. Furthermore, our study revealed that inhibiting TET-induced autophagy promotes the accumulation of reactive oxygen species (ROS) in pancreatic cancer cells, thereby enhancing apoptosis both in vitro and in vivo.

## Materials and methods

### Cell lines and regents

Human pancreatic cancer cell lines PANC-1 (RRID: CVCL_0480), SW1990 (RRID: CVCL_1723) and BxPC-3 (RRID: CVCL_0186) were purchased from the Type Culture Collection of the Chinese Academy of Sciences (Shanghai, China). The PANC-1 and SW1990 cells were cultured in RPMI-1640 medium supplemented with 10% fetal bovine serum (Gibco, USA) and 1% penicillin/streptomycin. The BxPC-3 cells were cultured in DMEM medium supplemented with 10% FBS and 1% penicillin/streptomycin. All the cell lines were maintained in a humidified chamber at 37 ℃ with 5% CO_2_.

Tetrandrine (TET) (Cat. SML3048, Sigma) was dissolved in dimethyl sulfoxide (DMSO) to 60 mg/mL as stock solution. Chloroquine (CQ) and acridine orange were purchased from Yuanye biotech (China). 3-methyladenine (3-MA) and bafilomycin A1 (Baf-A1) were purchased from Sigma (USA). (4,5-dimethylthiazol-2-yl)-3,5-diphenylformazan (MTT), an Annexin V/PI apoptosis kit and a mitochondrial membrane potential assay kit with JC-1 were purchased from Beyotime Biotechnology (China). Primary antibodies against LC3B (3868), P62 (23,214), AKT(4691), phosphor-AKT(Ser473) (4060), mTOR (2972), phosphor-mTOR(Ser2448) (2971), Cleaved-caspase3 (9664), Cleaved-caspase9 (20,750) and Cleaved-caspase PARP (5625) were purchased from CST(USA). Primary antibodies against Beclin-1(66665-1), Bcl-2(60178-1), ATG7(67341-1), ULK1 (29005-1-AP), SOD2 (66474-1), GAPDH (60004-1) were purchased from Proteintech (China). The primers for Real Time PCR were purchased from Sangon Biotech (China).

### Transmission electron microscopy

PANC-1 cells were treated with 10µM TET for 6 h, 24 h and then fixed with 2.5% glutaraldehyde for 2 h. After washing with phosphoric acid rinse solution, cells were subsequently fixed in 1% phosphate-buffered osmium tetroxide for 3 h. Then cells were stained with 3% aqueous uranyl acetate. Subsequently, samples were subjected to a gradient dehydration process using ethanol and acetone, followed by embedding and subsequent solidification. After sectioning the samples, dual staining was performed using a 3% uranyl acetate-lead citrate solution. A Philips EM420 transmission electron microscope was used to observe samples.

### Western blotting

Cells were treated with different reagents for different time. Cell samples were washed with PBS, and total protein was then extracted with RIPA lysis buffer containing 1% PMSF and 2% phosphatase inhibitors (Bytotime Biotechnology, China). Protein concentration was quantified using a BCA Protein Assay Kit (Bytotime Biotechnology, China). 20 µg total protein was loaded onto the sodium dodecyl sulfate polyacrylamide gel (10%,12.5%) and then separated by electrophoresis, and electrically transferred onto polyvinylidene difluoride (PVDF, Millipore) membranes. The membranes were blocked with 5% skim milk diluted in 1×TBST (0.1% Tween) for 2 h at room temperature, followed incubation with the relevant primary antibody at 4℃ overnight. After washing three times with TBST for 10 min each time, the membranes were incubated with second antibody for another 1 h at room temperature. The washing process was repeated 3 times and the protein immunoreactive bands were detected using an enhanced chemiluminescence (ECL) reagent (Affinity Biosciences, USA).

### Quantitative real-time PCR (qPCR) analysis

Cells (5 × 10^5^) were seeded in the six well culture plates. When cell fusion reached 80%, total RNA of cells was isolated using Lysis buffer (EZBioscience, China). 1 µg total RNA was used for cDNA synthesis. Genomic DNA was removed with gDNA Remover and then the RNA was mixed with RT master (EZBioscience, China) to synthesize cDNA. Quantitative reverse-transcriptase polymerase chain reaction (qRT-PCR) was performed using the SYBR Green Master (EZBioscience, China). All qRT-PCR experiments were conducted in triplicate. GAPDH was used to normalized data. 2^−ΔΔCt^ method was presented to determine the relative mRNA expression level. The primer sequences were as follows:

ATG7:

forward 5’-GGCAGGATAGCAAAACCAATA-3’.

reverse 5’-TGTATACCAACACACTCGA-3’.

ATG5:

forward 5’- TGTGCTTCGAGATGTGTG-3’.

reverse 5’- GTCAAATAGCTGACTCTTGG-3’.

GAPDH:

forward 5’- CAGGAGGCATTGCTGATGAT-3’.

reverse 5’- GAAGGCTGGGGCTC-ATTT − 3’.

### Cell proliferation assay

MTT assay was used to detect cell viability. Approximately cells (6000/well) were seeded in 96-well plates, incubating at 37℃ overnight. The MTT assay was performed after appropriate treatment. The medium was removed and then 100µL medium which was mixed with 20% MTT (Sigma, USA) was added into the wells. Then the culture plates were incubated at 37℃ for 3 h. The medium was discarded and 110µL DMSO (Sigma, USA) was added into the wells, incubating at 37℃ for 20 min. After shaking the plates for 5 min, the absorbance was detected at an OD 490 nm using a microplate reader (BioRad).

### Apoptosis assay

Apoptosis was detected using an Annexin V/PI apoptosis kit. Cells were seeded in 6 well plates, incubating at 37℃ overnight. After designated treatment, the cell culture medium was aspirated and cells were washed once with PBS by gently swirling. ell dissociation solution containing trypsin was added to digest the cells. The cells were incubated at room temperature until gentle tapping allowed the adherent cells to detach. Cells were collected and resuspended with Annexin V-FITC binding solution. 5µL Annexin V-FITC and 10µL propidium iodide staining solution were added and gently mix. Cell samples were incubated at room temperature (20–25 °C) in the dark for 10–20 min, followed by placing it on ice. During the incubation, the cells can be gently resuspended 2–3 times to improve staining efficiency. Apoptosis was then examined using a flow cytometry.

### Measurement of ROS

Cells were seeded in 96-well plates overnight and then treated with different reagents. Dichlorodihydrofluorescein diacetate (DCFH-DA) (Bytotime Biotechnology, China) was diluted in serum-free culture medium at a ratio of 1:1000, resulting in a final concentration of 10µM. The cell culture medium was then removed, and 1 ml diluted DCFH-DA was added into plate. Cells were stained at 37℃ for 30 min and then washed with serum-free medium for three times. Cells were captured using a fluorescence microscope.

Cells were seeded in 96-well plates overnight and then treated with different reagents. The process of staining was mentioned as above. A fluorescence microplate (excitation wavelength: 488 nm; emission wavelength: 525 nm) was used to examine the levels of fluorescence (ROS levels).

### Measurement of mitochondrial membrane potential (MMP)

The mitochondrial membrane potential was measured by JC-1 assay. After treatment, cells were washed and collected to resuspend in the culture medium. JC-1 staining working solution (Beyotime Biotechnology, China) was added and thoroughly mixed. Cells were then incubated at 37 °C in a cell culture incubator for 20 min. After the 37 °C incubation period, the cells were centrifuged at 600 g and 4 °C for 3–4 min to pellet the cells. The supernatant was discarded, and JC-1 staining buffer (1X) was added to resuspend the cells. The cells were then washed in this way twice. Flow cytometry (excitation wavelength: 490 nm; emission wavelength:530 nm) was used to examine MMP.

### 3D organoid model

PDAC tissues were obtained from Department of Surgery, Shanghai Sixth People’s Hospital affiliated to Shanghai Jiaotong University School of Medicine. No chemotherapy, radiotherapy, or immunotherapy was received pre-surgery. This study was approved by the Research Ethics Committee of Shanghai Sixth People’s Hospital. A 2 cm^3^ fresh tissue was cut and soaked in PBS containing 5% penicillin–streptomycin and antibiotics (2% primocin, Invitrogen, California, USA) for 30 min, and then, minced for enzymatic digestion by 1.5 mg/mL collagenase IV (7426, STEMCELL, British Columbia, Canada) for 1 h at 37 °C. Cells were washed twice by advanced DMEM/F12, and filtered by 40 μm cell strainer (27,305, STEMCELL, British Columbia, Canada). After centrifuged for 5 min at 1000 rpm, cells were seeded into Matrigel (356,235, Corning, New York, USA) and overlayed with human IntestiCult™ organoid growth medium (06010, STEMCELL, British Columbia, Canada) containing 10 µM Y-27632,1% Primocin and 1% penicillin–streptomycin. TrypLE™ Express Enzyme (12,604,021, Thermo Scientific, Massachusetts, USA) was used to digest and resuspended cancer cells for organoid passages.

### Xenograft tumor model

Six weeks-old male BALB/c nude mice (Shanghai Si Lai Ke Laboratory Animal Co. Ltd, China) were housed in a pathogen-free room in the Animal Experiment Center of Shanghai Jiaotong University Affiliated Sixth People’s Hospital. PANC-1 cells(8 × 10^6^) were subcutaneously injected into axillary region of mice. After xenograft tumors grew to be palpable, 16 mice were randomly divided into four groups and treated with either saline, TET (30 mg/kg, once every three days), CQ (60 mg/kg, once every three days), or a combination of CQ (60 mg/kg) and TET (30 mg/kg) (once every three days). Tumors size was measured every 4 days. All the mice were euthanized under general anesthesia four weeks after 28-days treatment.

### Immunohistochemical (IHC) analysis

Immunohistochemical (IHC) staining and quantification were performed on xenograft tumors according to previous studies [23, 24]. The staining intensity was ranged from 0 to 3 (0, no staining; 1, light staining; 2, moderate staining; 3, dark staining). The percentage score of positively staining cells was ranged from 0 to 3 (1, < 5%; 2, 5–30%; 3, 31–70%; 4, > 70%). The final staining score was calculated by multiplying the two scores.

### Statistical analysis

Data were presented as mean ± SD and P-value was determined using the Student’s t-test and one-way ANOVA. P-values (two-side) < 0.05 were considered to be statistically significant. The Graphpad software was used for statistical analysis.

## Results

### Tetrandrine (TET) induces autophagy in pancreatic cancer cells

**Fig. 1:**
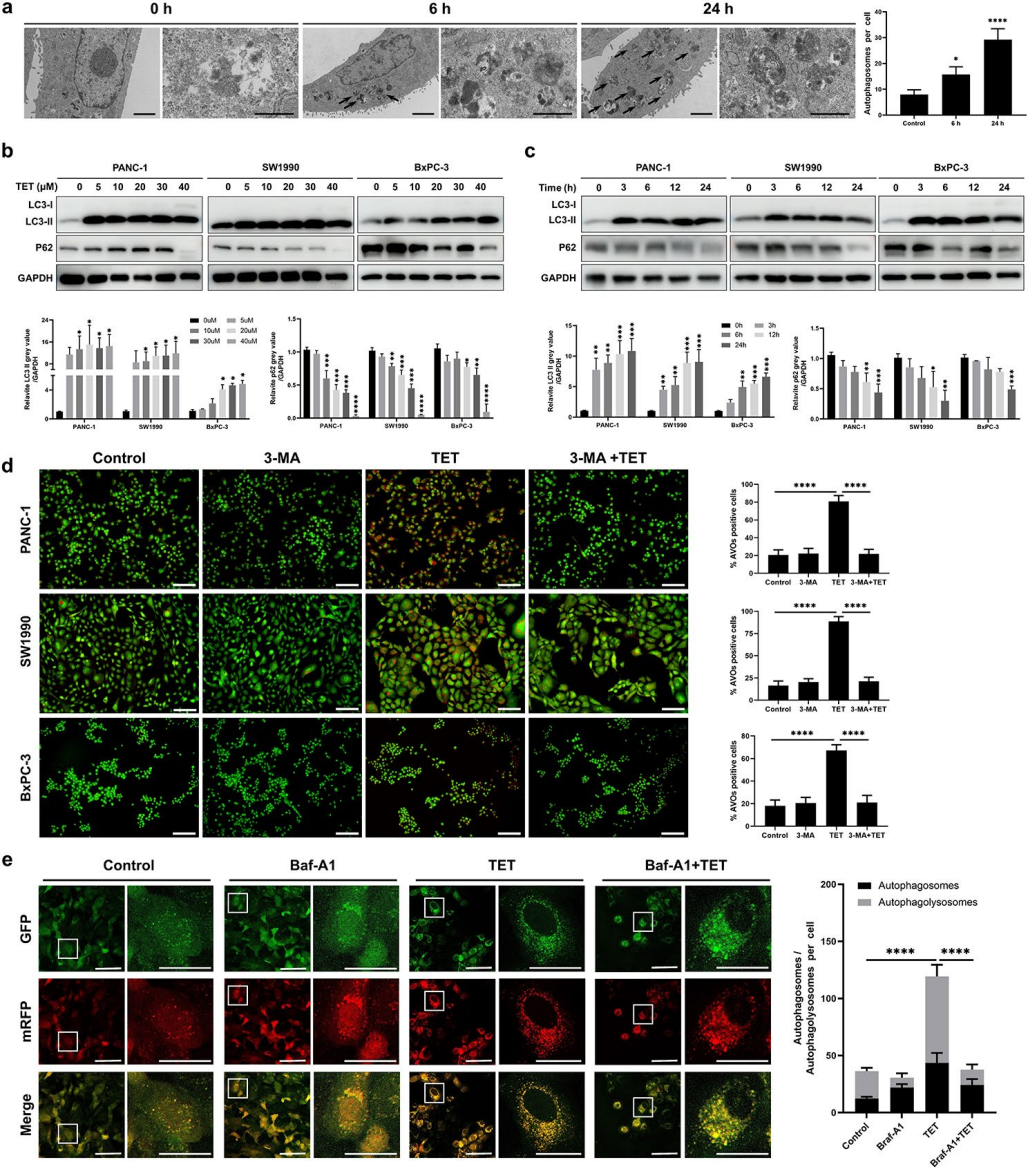
Tetrandrine (TET) induces autophagy in pancreatic cancer cells. **a** PANC-1 cells were treated with or without TET for 6 and 24 h, and transmission electron microscopy was used to observe autophagosomes. **b, c** PANC-1, SW1990 and BxPC-3 cells were treated with TET in gradient concentrations for 24 h. PANC-1, SW1990 and BxPC-3 cells were treated with TET for indicated time. LC3 and p62 protein expression was detected by western blot. **d** PANC-1, SW1990 and BxPC-3 cells were pretreated with 10mM 3-MA for 24 h and then treated with 10µM TET for another 24 h. Acidic vesicular organelles (AVOs) were detected with acridine staining, and the number of cells with AVOs was quantified. Scale bars indicate 200 μm. **e** SW1990 cells were transfected with GFP-mRFP-LC3 and then treated with TET and Bafilomycin A1 for 24 h. Cells were observed using a fluorescence microscope and the number of autophagosomes (yellow dots) and autolysosomes (red dots) was counted. Scale bars indicate 50 μm.c Data was presented as mean ± SD of three independent experiments.* indicates *p*<0.05, *** indicates *p*<0.005, **** indicates *p*<0.001

TET induces autophagy and apoptosis in various tumors, exhibiting distinct biological effects. To investigate whether TET induces autophagy in pancreatic cancer cell, we utilized transmission electron microscopy. Under transmission electron microscopy, a significant increase in the number of autophagosomes was observed in PANC-1 cells following TET treatment, and the effect is time-dependent. (Fig. 1a). Immunoblot analysis was performed on PANC-1 SW1990 and BxPC-3 cells after TET treatment. The results revealed the conversion of LC3I to LC3II and enhanced degradation of p62. Moreover, these effects were enhanced with increasing concentrations of TET and prolonged treatment time (Fig. 1b, c). Autophagy generates numerous acidic vesicular organelles (AVOs), thus we employed acridine orange staining to detect AVOs in TET-treated pancreatic cancer cells. The results indicated that TET stimulated the production of more AVOs in pancreatic cancer cells, and the aggregation of AVOs was significantly reduced after co-treatment with the autophagy inhibitor 3-MA (Fig. 1d). To further elucidate the impact of TET on the autophagic flux in pancreatic cancer cells, we performed GFP-mRFP-LC3 lentiviral transduction in SW1990 cells. Following TET treatment, red fluorescent puncta were observed in pancreatic cancer cells, while the green fluorescent signal, indicative of GFP degradation in acidic environments, was diminished in the cells. We also found co-localization of red and green fluorescent puncta in cells treated with the autophagy inhibitor Baf-A1 in combination with TET (Fig. 1e). The result indicated that TET promotes the accumulation of autophagosomes and the fusion with lysosomes, thereby enhancing autophagic flux in pancreatic cancer cells. These findings collectively demonstrate that TET can induce autophagy in pancreatic cancer cells.

### Tetrandrine induces autophagy by inhibiting AKT/mTOR pathway and activating autophagy-related gene 7 (ATG7)

**Fig. 2:**
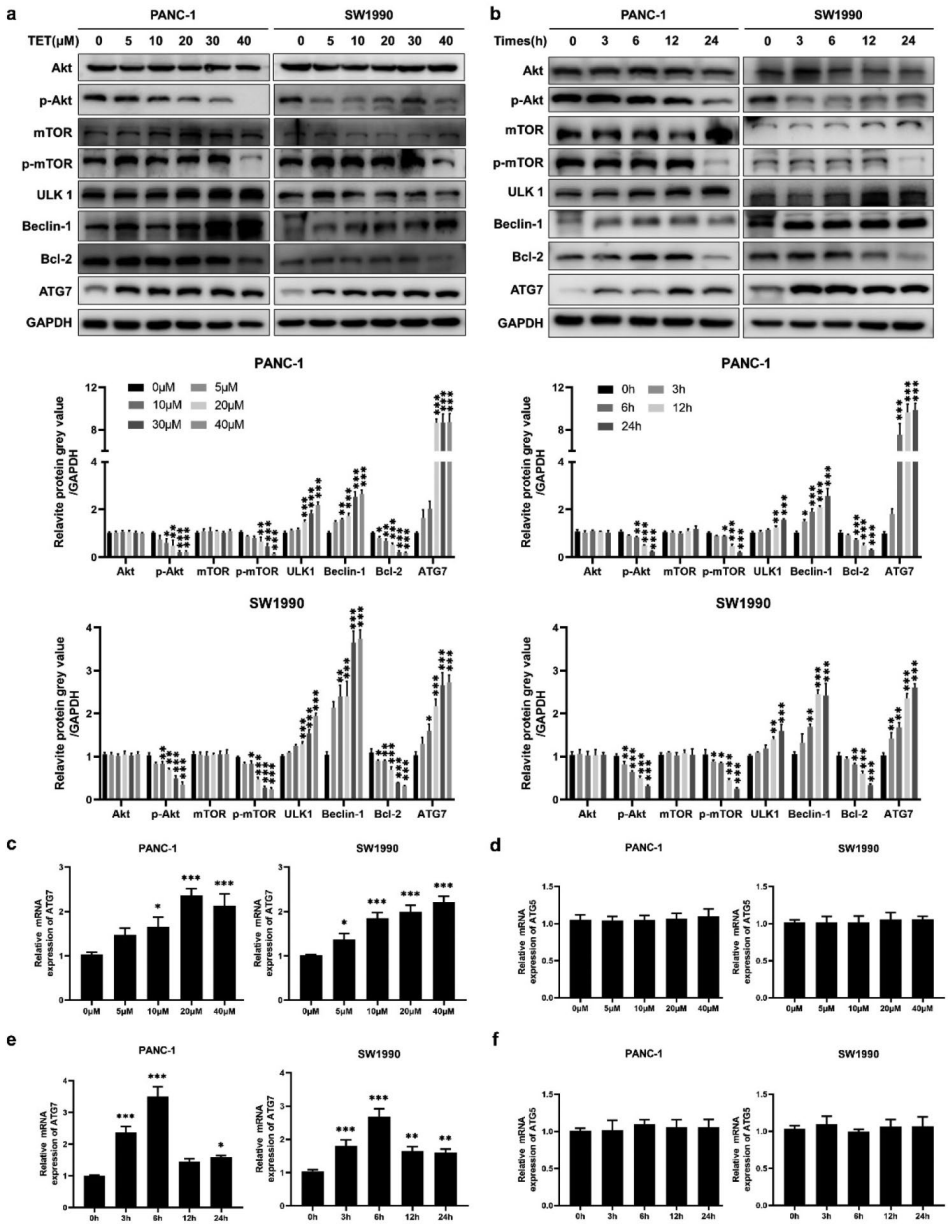
Tetrandrine induces autophagy by inhibiting AKT/mTOR pathway and activating autophagy-related Gene 7 (ATG7). **a** PANC-1, SW199 and BxPC-3 cells were treated with TET in gradient concentrations for 24 h and the indicated protein expression was detected by western blot. **b** PANC-1, SW199 and BxPC-3 cells were treated with TET for 0,1,6,12,24 h and the indicated protein expression was detected by western blot. **c, d** PANC-1, SW199 and BxPC-3 cells were treated with TET in gradient concentrations for 24 h and the mRNA levels of ATG7 and ATG5 were detected by q-PCR. **e, f** PANC-1, SW199 and BxPC-3 cells were treated with TET for 0,1,6,12,24 h and the mRNA levels of ATG7 and ATG5 were detected by q-PCR. Data was presented as mean ± SD of three independent experiments.* indicates *p*<0.05, ** indicates *p*<0.01,*** indicates *p*<0.005, ****indicates *p*<0.0005

Inhibition of the AKT/mTOR pathway is a crucial mechanism for activating autophagy. We examined the expression of AKT/mTOR pathway proteins in PANC-1 and SW1990 cells treated with TET through immunoblot analysis. We found that phosphorylated AKT and mTOR in the cells were inhibited in a dose-dependent and time-dependent manner following TET treatment. Subsequently, the expression of ULK1 was upregulated after TET treatment. Furthermore, protein immunoblotting experiments indicated that TET could suppress the expression of Bcl-2 in pancreatic cancer cells. The inhibition of these proteins further led to the upregulation of Beclin-1 expression (Fig. 2a, b). Additionally, we observed that TET promoted the expression of ATG7 protein in pancreatic cancer cells in a dose-dependent manner (Fig. 2a, b). To further validate the regulation of ATG7 by TET, we performed qPCR and demonstrated that TET treatment resulted in an upregulation of ATG7 mRNA levels (Fig. 2c, e). In addition, the changes of ATG7 mRNA levels appeared 6 h after TET treatment, preceding the changes in protein expression. We also explored whether TET affects the expression of ATG5. As shown in Fig. 2d, f, the expression of ATG5 was not affected after TET treatment. These findings indicate that TET inhibits the AKT/mTOR pathway and promotes the transcription of ATG7 to activate autophagy in pancreatic cancer cells.

### Inhibition of TET-induced autophagy enhances the apoptotic effect of TET in pancreatic cancer cells

**Fig. 3:**
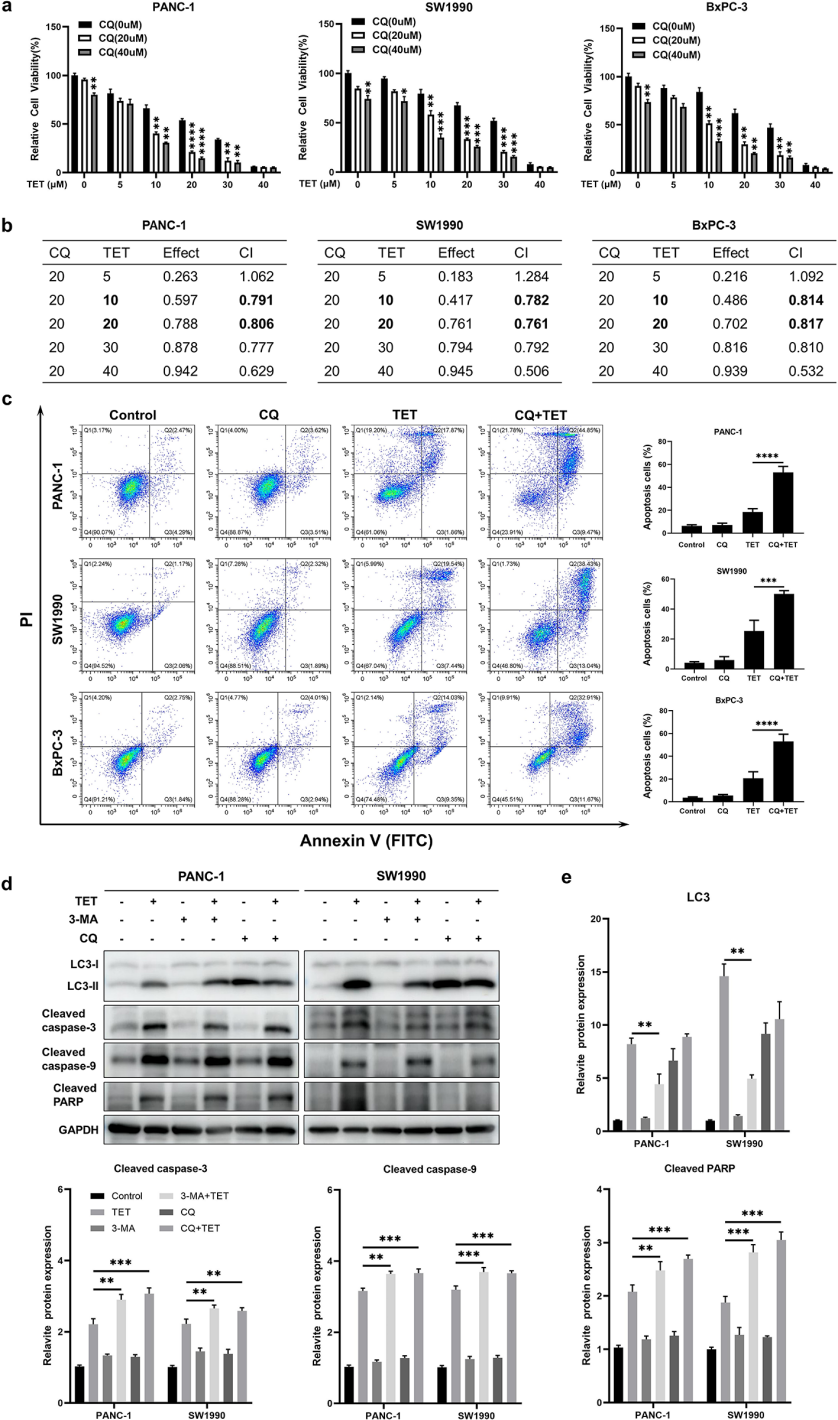
Inhibition of TET-induced autophagy enhances the pro-apoptotic effect of TET in pancreatic cancer cells. **a** MTT assay shows cell viability of PANC-1, SW1990 and BxPC-3 cells treated with CQ and TET in gradient concentrations. **b** The combination index of TET and CQ in three kinds of pancreatic cancer cell. **c** PANC-1, SW1990 and BxPC-3 cells were treated with 20µM CQ or 10µM TET. Flow cytometry was performed to detected apoptosis with Annexin V/PI double stain. **d** PANC-1 and SW1990 cells were pretreated with 10mM 3-methyladenine (3-MA) or 20µM CQ for 24 h, and then treated with 10µM TET for another 24 h. The expression of LC3II, cleaved-PARP, cleaved caspase-3, and cleaved caspase-9 proteins was detected using western blot. **e** The quantification of western blot. Data was presented as mean ± SD of three independent experiments. ** indicates *p*<0.01, *** indicates *p*<0.005, **** indicates *p*<0.001

**Fig. 4:**
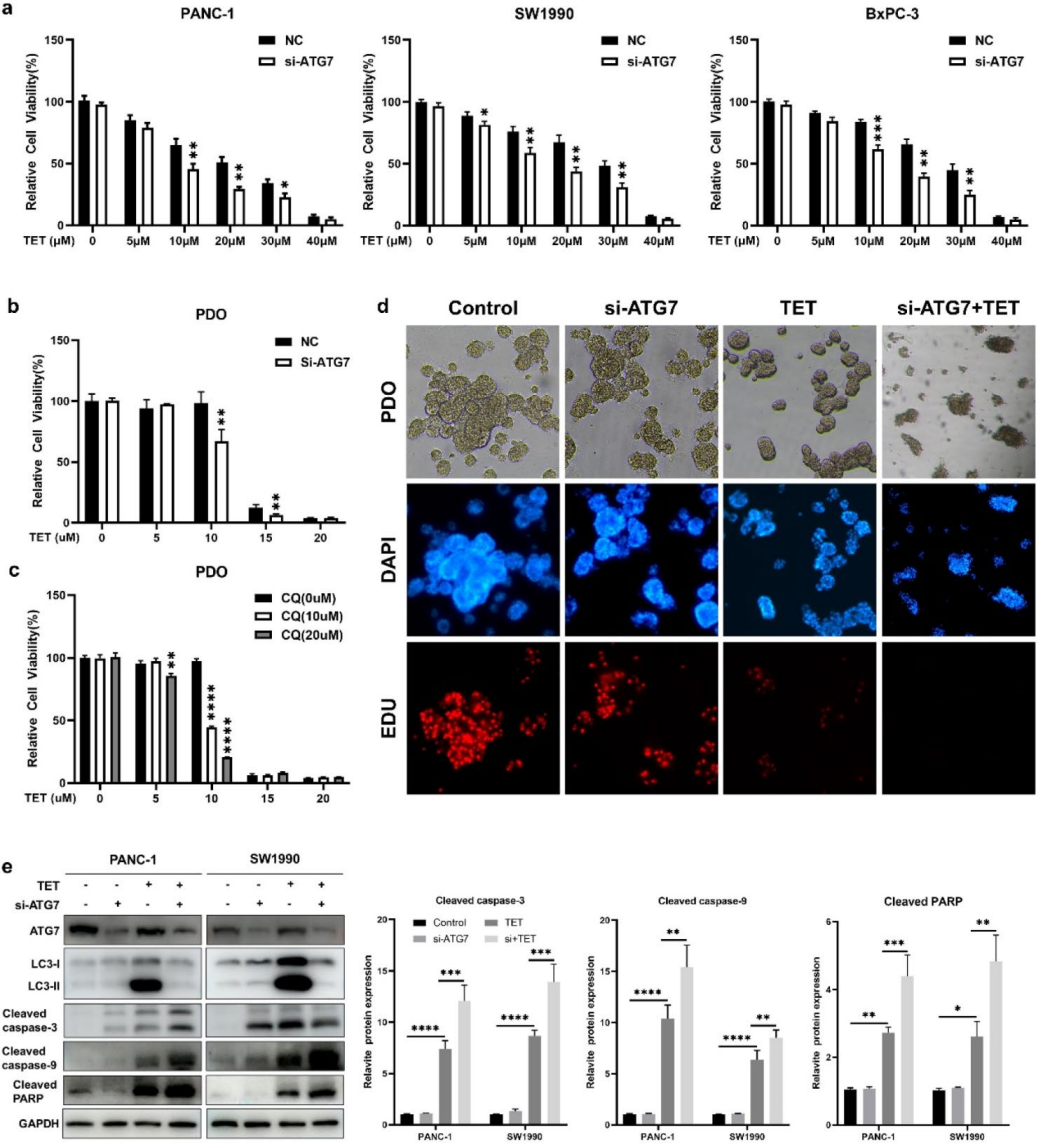
Inhibition of TET-induced autophagy by interfering with ATG7 expression enhances the toxicity of TET in pancreatic cancer cells. **a** PANC-1, SW1990 and BxPC-3 cells were transfected with si-RNA of ATG7 for 48 h and then treated with TET for another 24 h. Cell viability was detected with MTT assay. **b** Organoids constructed from PDAC tissues was transfected with si-RNA of ATG7 for 48 h and then treated with TET for another 24 h. Cell viability was detected with MTT assay. **c** PDAC organoids was treated with CQ and TET. Cell viability was detected with MTT assay. **d** The proliferation of PDAC organoid was detected using EDU staining(100×). **e** The expression of LC3II, cleaved-PARP, cleaved caspase-3, and cleaved caspase-9 proteins was detected using western blot. Data was presented as mean ± SD of three independent experiments. ** indicates *p*<0.01, *** indicates *p*<0.005, **** indicates *p*<0.001

Based on previous findings, we have observed that TET can induce apoptosis and autophagy in pancreatic cancer. To elucidate the relationship between TET-induced autophagy and apoptosis, we inhibited TET-induced autophagy and observed its impact on apoptosis in pancreatic cancer cells. Initially, PANC-1, SW1990, and BxPC-3 cells were pre-treated with the lysosome inhibitor CQ for 24 h, followed by combined treatment with TET for another 24 h. Cell proliferation and apoptosis assays were conducted. The results of the cell proliferation assay demonstrated a significant increase in cytotoxicity of TET when cells were pre-treated with CQ (Fig. 3a). The combination index (Fig. 3b) indicated the synergistic effect between CQ and TET. As shown in Fig. 3c, the apoptosis assay results also indicated that CQ combined TET significantly promoting apoptosis in pancreatic cancer cells. Furthermore, we pre-treated PANC-1 and SW1990 cells with two autophagy inhibitors, CQ and 3-MA, followed by the addition of TET. Immunoblot analysis revealed a significant upregulation of cleaved-PARP, cleaved caspase-3, and cleaved caspase-9 expression after combined treatment with TET and autophagy inhibitors (Fig. 3d, e). To further investigate the impact of autophagy inhibition on TET-induced cytotoxicity, we used siRNA to interfere with the expression of ATG7. We found that TET exhibited significantly enhanced cytotoxicity in pancreatic cancer cells after inhibiting ATG7 expression (Fig. 4a). Additionally, Western blot showed that inhibiting ATG7 expression yielded the same effect as the pharmacological inhibition of autophagy combined with TET treatment (Fig. 4e). Furthermore, we successfully constructed organoids from PDAC tissue. MTT assay and EDU staining showed that inhibition of autophagy enhanced cytotoxicity of TET in PDAC organoid (Fig. 4b, c,d), which showed that interference of ATG7 effected similarly with CQ. Thus, inhibiting TET-induced autophagy can enhance the pro-apoptotic effect of TET in pancreatic cancer cells.

### The generation of reactive oxygen species (ROS) is also involved in the TET-induced autophagy and apoptosis

**Fig. 5:**
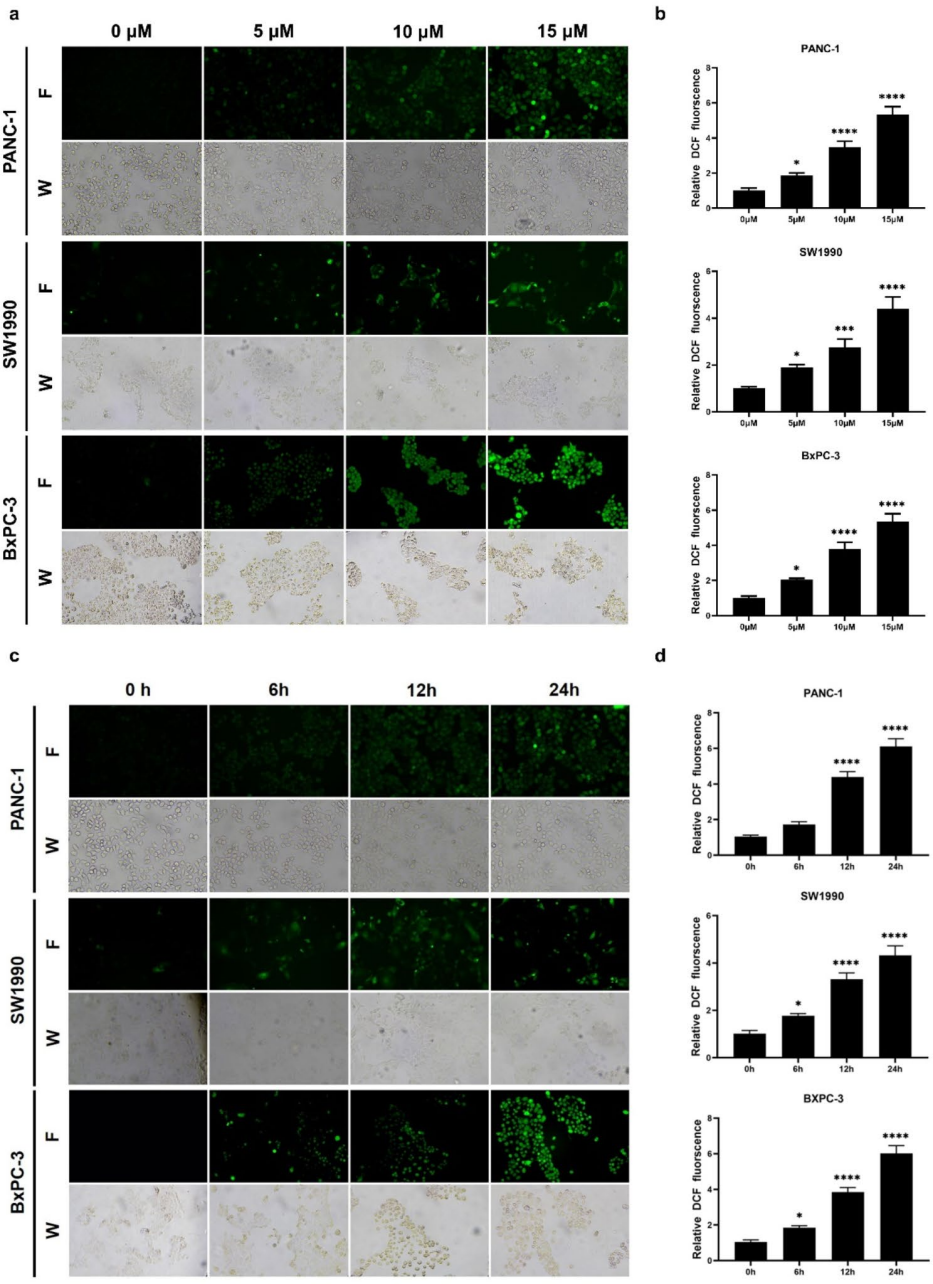
TET induce the generation of ROS in pancreatic cancer cells. PANC-1, SW199 and BxPC-3 cells were treated with TET in gradient concentrations for 24 h or at different time point. DCFH-DA staining was performed. **a, c** The generation of ROS was visualized under a fluorescence microscope (×100). **b, d** The levels of ROS production were measured using a fluorescence microplate. Data was presented as mean ± SD of three independent experiments. * indicates *p*<0.05, *** indicates *p*<0.005, **** indicates *p*<0.001

**Fig. 6:**
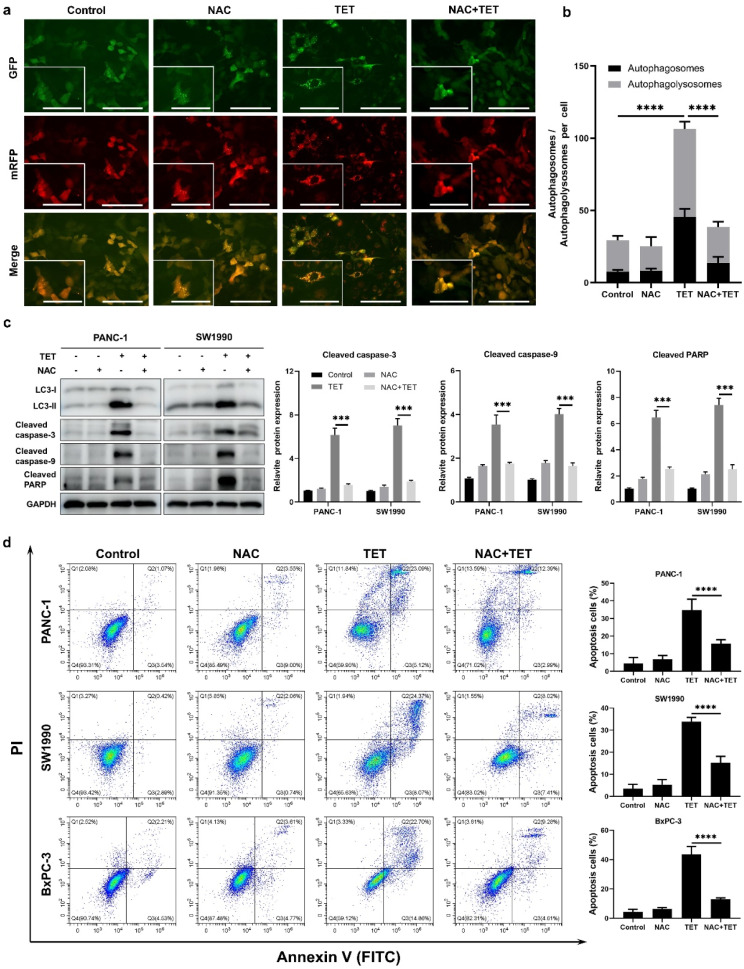
The generation of reactive oxygen species (ROS) is also involved in the TET-induced autophagy and apoptosis. **a** Stable GFP-mRFP-LC3 transfected SW1990 cells were treated with 20µM TET or 20mM NAC and observed under a fluorescence microscope. Scale bar indicate 200 μm. **b** Count of autophagosomes (yellow dots) and autolysosomes (red dots). **c** PANC-1 and SW1990 cells were treated with 10µM TET or 20mM NAC for 24 h and the expression of LC3II, cleaved-PARP, cleaved caspase-3, and cleaved caspase-9 proteins was detected using western blot. **d** PANC-1, SW1990 and BxPC-3 cells were treated with 20µM TET or 20mM NAC for 24 h. Apoptosis was detected using Annexin V/PI double stain and flow cytometry was performed. Data was presented as mean ± SD of three independent experiments. * indicates *p*<0.05, *** indicates *p*<0.005, **** indicates *p*<0.001

The generation of reactive oxygen species (ROS) is not only a significant factor in activating autophagy but also in inducing cell apoptosis, as demonstrated in various tumor cells [25, 26]. Therefore, we examined the levels of ROS in pancreatic cancer cells after treatment with TET using fluorescence microscopy and a fluorescence microplate reader. As shown in Fig. 5a-d, TET significantly increased the levels of ROS in pancreatic cancer cells, following a dose-dependent and time-dependent pattern. To further clarify whether ROS are involved in TET-induced autophagy and apoptosis in pancreatic cancer cells, we used the ROS scavenger NAC to suppress the production of ROS in pancreatic cancer cells. Detection of autophagic flux in pancreatic cancer cells SW1990 revealed a decrease in yellow and red fluorescence in cells when ROS production stimulated by TET was inhibited with NAC (Fig. 6a, b). This suggests that the inhibition of ROS generated by TET can suppress autophagic flux in pancreatic cancer cells. Furthermore, the results of the apoptosis assay demonstrated that the combined use of NAC and TET significantly inhibited the cytotoxicity of TET in pancreatic cancer cells (Fig. 6d). This indicates that the accumulation of ROS also contributes to TET-induced apoptosis in pancreatic cancer cells. Additionally, immunoblotting results showed that the expression of cleaved-PARP, cleaved-caspase3, and cleaved-caspase9 in PANC-1 and SW1990 cells decreased when ROS were inhibited by NAC (Fig. 6c). These findings indicate that TET can stimulate the production of ROS in pancreatic cancer cells, and the generation of ROS plays an important role in both TET-induced autophagy and apoptosis.

### Inhibition of TET-induced autophagy promotes the accumulation of ROS and apoptosis in pancreatic cancer cells

**Fig. 7:**
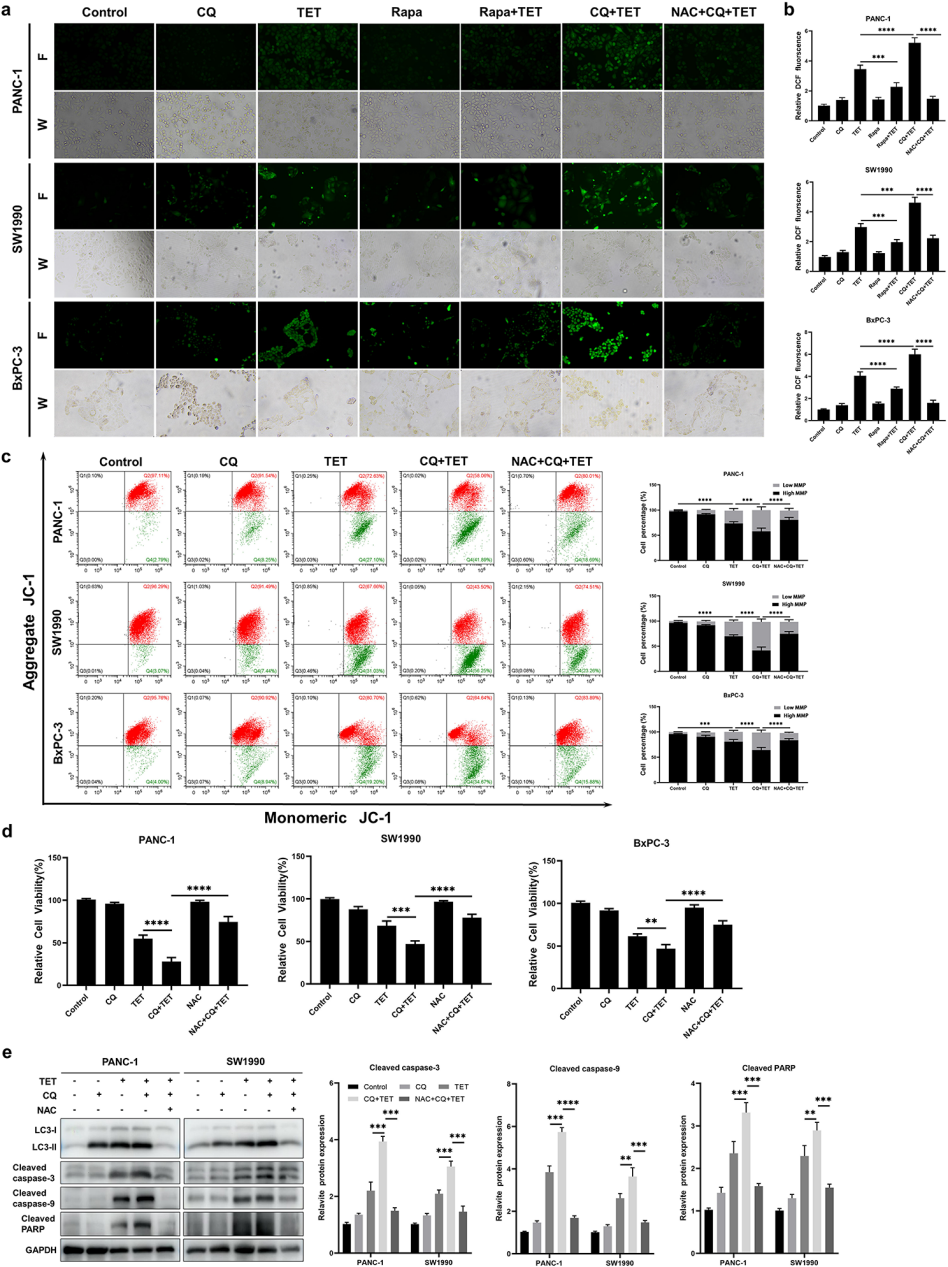
Inhibition of TET-induced autophagy promotes apoptosis in pancreatic cancer cells through the accumulation of ROS. **a** PANC-1, SW199 and BxPC-3 cells were pretreated with 20µM CQ or 20mM NAC or 150 nm Rapamycin (Rapa) for 24 h and then treated with TET for another 24 h. DCFH-DA staining was performed. The generation of ROS was visualized under a fluorescence microscope (×100). **b** The levels of ROS production were measured using a fluorescence microplate. **c** PANC-1, SW199 and BxPC-3 cells were pretreated with 20µM CQ or 20mM NAC for 24 h and then treated with TET for another 24 h. JC-1 assay was performed to detected the mitochondrial membrane potential. **d** Cell viability was detected using MTT assay. **e** The expression of LC3II, cleaved-PARP, cleaved caspase-3, and cleaved caspase-9 proteins was detected using western blot. Data was presented as mean ± SD of three independent experiments. ** indicates *p*<0.01, *** indicates *p*<0.005, **** indicates *p*<0.001

Autophagy is an important pathway through which ROS is cleared in cells. Based on the aforementioned research findings, we hypothesized that the enhanced cytotoxicity of TET induction could be attributed to the accumulation of ROS. To verify our hypothesis, we inhibited autophagy with CQ and measured the ROS levels in pancreatic cancer cells. The results showed a significant increase in ROS accumulation after treatment with CQ and TET induction. Conversely, when autophagy was stimulated using the autophagy agonist, rapamycin, the ROS levels in pancreatic cancer cells significantly decreased (Fig. 7a, b). Accumulated ROS in cells can induce lipid peroxidation, leading to the disruption of various organelle membrane structures, including mitochondria. Therefore, we examined the mitochondrial membrane potential in pancreatic cancer cells. We found that TET treatment resulted in a decrease in mitochondrial membrane potential. Furthermore, when cells were pretreated with CQ, TET further reduced the mitochondrial membrane potential (Fig. 7c). These findings highlight the crucial role of autophagy in ROS clearance in pancreatic cancer cells. Moreover, the combination of CQ and TET-induced cell death was significantly reduced after pretreatment with NAC (Fig. 7d). Western blot analysis revealed an increase in the expression of apoptosis-related proteins, cleaved-PARP, cleaved-caspase3, and cleaved-caspase9, after co-treatment with CQ and TET, consistent with previous experimental results. However, the expression of these proteins significantly decreased when intracellular ROS was eliminated by NAC (Fig. 7e). These experimental results indicate that the enhanced cytotoxicity of TET induction through autophagy inhibition is due to the accumulation of ROS.

### Inhibition of autophagy enhances TET-mediated cytotoxicity against pancreatic cancer in vivo

**Fig. 8:**
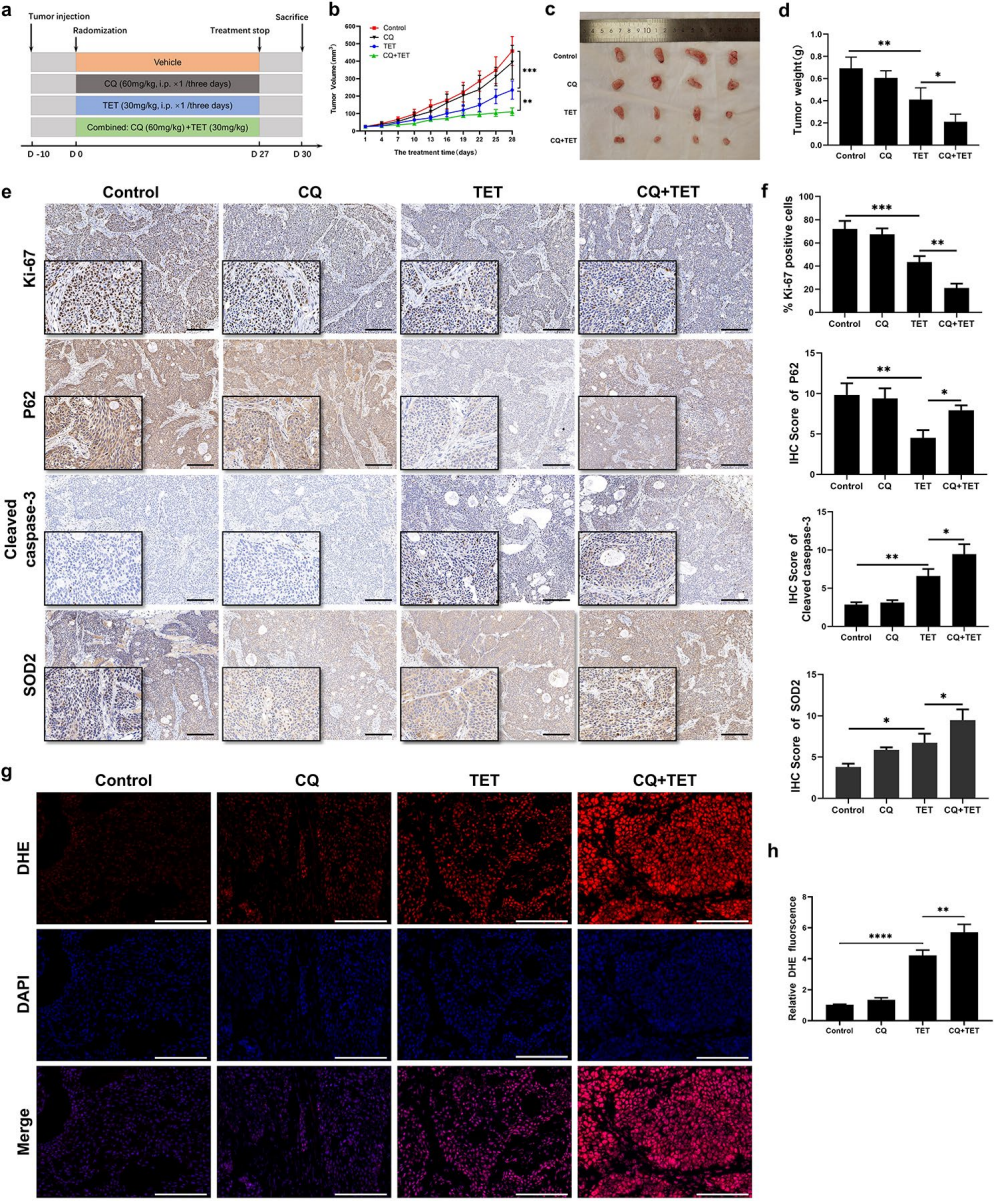
Inhibition of autophagy enhances TET-mediated cytotoxicity against pancreatic cancer in vivo. **a** Schematic illustration for the nude mouse experimental design. **b** Changes in tumor volume. **c** Xenograft tumors (*n* = 4). **d** Tumor weight(*n* = 4). **e** Immunohistochemical (IHC) assay of Ki-67, P62, cleaved caspase-3, SOD2 in tumor specimens. Scale bars indicate 200 μm. **f** Quantification of IHC staining of Ki-67(*n* = 5), P62 and cleaved caspase-3. **g** ROS accumulation in tumor specimens were detected using DHE staining. **h** Quantification of DHE fluorescent intensity (*n* = 5). Scale bars indicate 200 μm. Data was presented as mean ± SD. * indicates *p*<0.05, ** indicates *p*<0.01, *** indicates *p*<0.005, **** indicates *p*<0.001

According to the aforementioned results, we observed that TET can induce cytoprotective autophagy in pancreatic cancer cells, and inhibition of this autophagy can enhance the cytotoxicity of TET against pancreatic cancer in vitro. To further validate whether this mechanism exists in vivo, we established subcutaneous tumor xenografts of PANC-1 cells in nude mice. The tumor-bearing mice were randomly divided into four groups and treated with either saline, TET, CQ, or a combination of CQ and TET (Fig. 8a). After 30 days of treatment, the mice were euthanized, and the subcutaneous tumors were isolated. The results of the animal experiment showed that the volume of subcutaneous tumors in the TET treatment group were significantly smaller than those in the saline treatment group, and the combination with CQ further enhanced the inhibitory effect on tumor proliferation (Fig. 8b, c). The tumor weight of the mice during the treatment period also followed this trend (Fig. 8d). Furthermore, we performed immunohistochemical staining on the subcutaneous tumor tissues of the mice. The IHC results of Ki67 indicated the inhibitory effect of TET on tumor proliferation. In addition, the staining results of P62 suggested that TET induced autophagy in pancreatic cancer and can be inhibited by CQ. Moreover, the IHC analysis of cleaved caspase-3 indicated that TET induced apoptosis in pancreatic cancer cells. Importantly, the apoptotic effect was further enhanced when TET was combined with CQ (Fig. 8e, f). This observation is consistent with the results of in vitro experiments, further validating the synergistic effect of TET and CQ in the treatment of pancreatic cancer. To elucidate the role of TET in stimulating ROS production in pancreatic cancer subcutaneous tumors, we further performed IHC staining of SOD2 and DHE staining on the subcutaneous tumors. IHC staining of SOD2 indicated that treatment of TET led to an increased expression of SOD2 in pancreatic cancer, suggesting an elevated level of oxidative stress in the cells. Moreover, when TET was combined with chloroquine, the oxidative stress levels were further augmented. The result of DHE staining demonstrated that TET can stimulate the production of ROS in tumor cells, and the accumulation of ROS in tumor cells is significantly enhanced when combined with CQ (Fig. 8g, h). These results are consistent with the previous in vitro experiments, indicating that TET can induce autophagy in pancreatic cancer cells both in vitro and in vivo. Furthermore, inhibiting this autophagy can enhance its cytotoxicity. These findings highlight the therapeutic potential of a treatment strategy combining chemotherapy drugs with autophagy inhibitors.

## Discussion

Chemotherapeutic drugs purified from natural products, such as paclitaxel and camptothecin, have achieved tremendous success in tumor treatment, highlighting the potential of monomeric drugs extracted from plants in cancer therapy [27, 28]. While the anticancer effects of TET have been reported in various types of tumors, its efficacy in pancreatic cancer treatment is rarely studied. In the present study, we discovered that TET induced both cytoprotective autophagy and apoptosis in pancreatic cancer while stimulating the production of ROS. More remarkably, inhibiting TET-induced autophagy further enhanced its cytotoxicity in pancreatic cancer both in vitro and vivo.

Autophagy, an ancient and widespread cellular metabolic process, exists at a basal level in all cells and can be further activated by various stimuli [29]. In our study, we observed that TET stimulated the formation of autophagosomes, promoted autophagic flux. Meanwhile, TET was found to increase the conversion of LC3I to LC3II and enhance the degradation of p62 in a time- and dose-dependent manner. These findings indicated that TET significantly induced autophagy in pancreatic cancer cells beyond the basal level.

The process of autophagosome formation involves a series of coordinated events with the involvement of multiple proteins and regulation by various signal pathways. The initiation of autophagy starts with the formation of the ULK1 complex, and inhibition of the AKT/mTOR signaling pathway is known to be one of the key signaling pathways for autophagy activation [30–32]. Our study revealed that TET inhibited the phosphorylation of AKT and mTOR in pancreatic cancer cells in a dose- and time-dependent manner. Evidence has shown that mTOR regulates the activation of autophagy by modulating the ubiquitination of ULK1 [33]. Our findings also indicated that TET promoted the upregulation of ULK1 protein expression in pancreatic cancer cells. Additionally, TET promoted the expression of Beclin-1, partly by inhibiting the AKT/mTOR signaling pathway. Previous studies proved that the binding of Beclin-1 to Bcl-2 leads to its downregulation and inhibition of its activity [34, 35]. In our research, we found that TET inhibited the expression of Bcl-2. Consequently, the inhibition of Bcl-2 is another pathway through which TET activates autophagy in pancreatic cancer. Autophagy-related genes, including ATG5 and ATG7, play a critical role in autophagosome formation [36]. Our results demonstrated that TET promotes the transcription and protein expression of ATG7 in pancreatic cancer cells, suggesting that TET also regulates autophagy by promoting the formation of autophagosomes. Moreover, TET stimulated the production of ROS in pancreatic cancer cells in a dose- and time-dependent pattern, indicating the involvement of ROS in TET-induced autophagy. These results collectively demonstrate that TET-induced autophagy regulation occurs through multiple pathways in pancreatic cancer cells. However, the precise role of autophagy in pancreatic cancer remains to be elucidated.

As an essential cellular degradation system dependent on lysosomes, autophagy plays a crucial role in maintaining cellular homeostasis [8]. Previous studies reported that the inhibition of autophagy in normal tissue cells leads to various diseases [37–39]. In our study, we observed that inhibiting TET-induced autophagy through drugs or genetic means enhances its toxicity to pancreatic cancer by promoting mitochondria-dependent apoptosis, which was further confirmed in vivo. Furthermore, enhancing autophagy resulted in a further reduction of cytotoxicity in pancreatic cancer cells, indicating that autophagy induced by TET functions as a protective mechanism. Cytoprotective autophagy is a significant factor contributing to tumor cell resistance to chemotherapy drugs and can be induced by the chemotherapy drugs themselves or by the surrounding stress environment [40, 41]. Therefore, autophagy has emerged as a novel target in cancer treatment [42].

Reactive oxygen species (ROS) are highly reactive and oxidizing small molecules produced during cellular metabolism and have been closely associated with autophagy and apoptosis [43, 44]. ROS can react with various cellular substances, including DNA, lipids, and proteins [45]. Particularly, the oxidation of polyunsaturated fatty acids by oxidative radicals is susceptible to lipid peroxidation, which promotes autophagy and apoptosis [46]. Although ROS are primarily produced by mitochondria as byproducts of respiration, excessive accumulation of ROS can lead to lipid peroxidation of mitochondrial membranes, resulting in mitochondrial damage, which is an important factor in ROS-induced apoptosis [44]. Our study revealed that reducing the generation of ROS led to a decrease in autophagy levels and a significant attenuation of TET-induced apoptosis, confirming the involvement of ROS in both TET-induced autophagy and apoptosis in pancreatic cancer cells.

Chemotherapeutic agents have been found to induce autophagy in various cancer [47, 48]. Oxidative stress induced by ROS can stimulate the production of autophagy. As a metabolic pathway, autophagy can remove damaged mitochondria and reduce cellular ROS levels. However, if cellular clearance mechanisms fail to maintain homeostasis and stress persists, cells undergo apoptosis [49]. This provides the rationale for the therapeutic strategy of enhancing chemotherapy sensitivity by combining autophagy inhibitors with chemotherapy drugs. It is well known that pancreatic cancer has a high frequency of Kras mutations and RAS pathway mutations are closely associated with high levels of autophagy [50, 51]. Therefore, pancreatic cancer is highly sensitive to changes in autophagy status. Studies in Kras-mutant pancreatic cancer mice have shown that the absence of autophagy genes such as ATG5 or ATG7 blocks the progression from low- to high-grade pancreatic intraepithelial neoplasia and pancreatic ductal adenocarcinoma [52]. Thus, inhibiting autophagy holds great potential in pancreatic cancer treatment. However, inhibiting autophagy alone does not lead to satisfactory results in combating pancreatic cancer. In our study, individually inhibiting autophagy using drugs or genetic methods did not result in significant apoptosis in pancreatic cancer cells. Additionally, using chloroquine (CQ) alone to inhibit autophagy did not lead to excessive accumulation of ROS or enhanced apoptosis. These findings are consistent with previous clinical research [53]. In contrast, the combination therapy of CQ with TET showed significant efficacy. The context-dependent nature of autophagy in cells may explain these results. In summary, our research demonstrates the anticancer effects of TET on pancreatic cancer while also activating autophagy. Inhibiting autophagy can enhance the anticancer effects of TET on pancreatic cancer, supporting the exploration of combined autophagy inhibition strategies during chemotherapy for pancreatic cancer. Genetic methods for autophagy inhibition may result in non-selective effects on normal cells, leading to certain side effects. Currently, autophagy inhibitors appear to be a preferable choice. However, the selection of more suitable autophagy inhibitors remains an area for further research.

## Conclusions

**Fig. 9:**
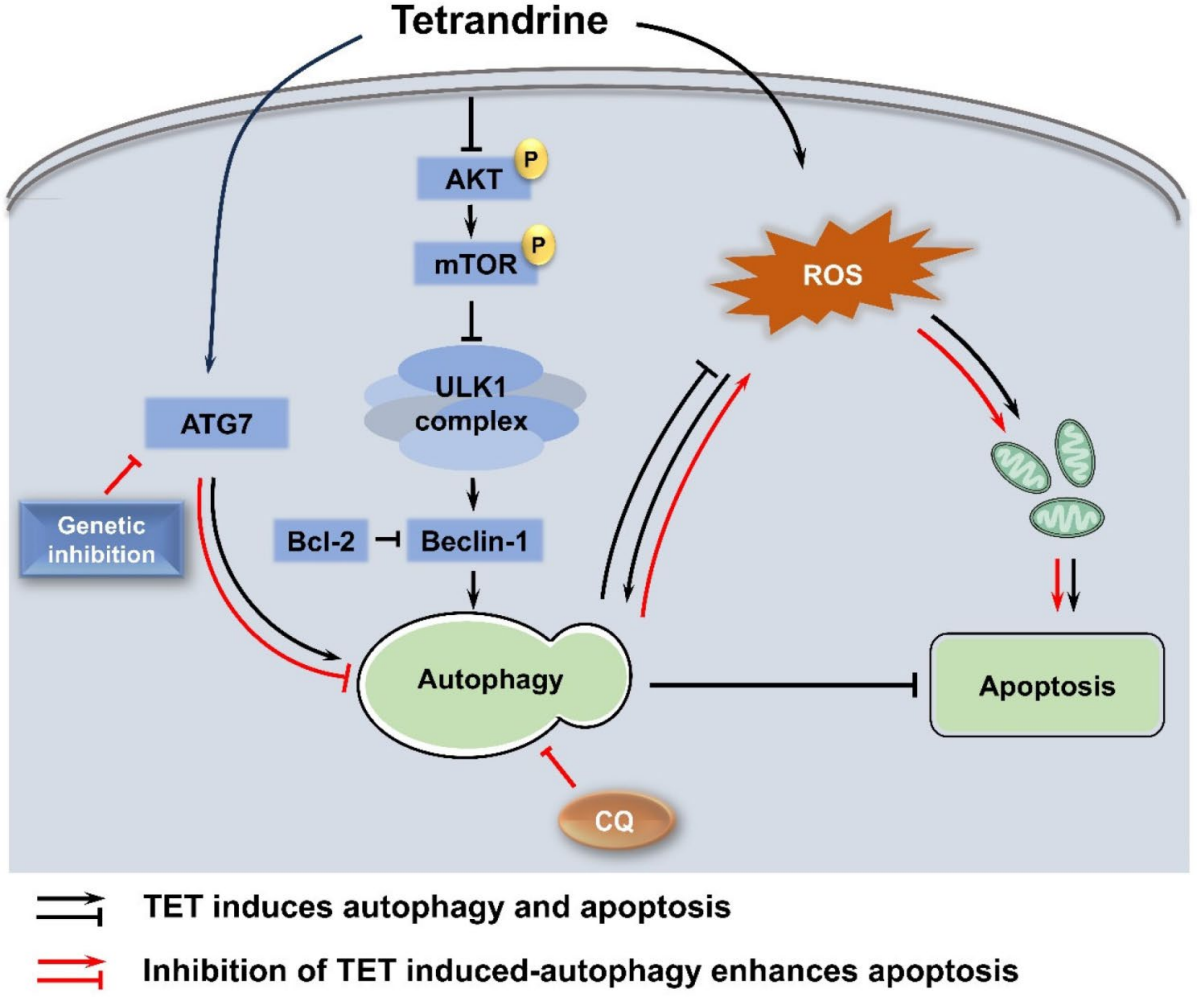
Inhibition of autophagy induced by tetrandrine promotes the accumulation of reactive oxygen species (ROS) and sensitizes efficacy of tetrandrine in pancreatic cancer

Based on the above, as depicted in Fig. 9, our results show that TET activates autophagy in pancreatic cancer cells through the inhibition of the AKT/mTOR signaling pathway and modulates autophagy by promoting the transcription of ATG7. Additionally, TET induces the production of reactive oxygen species (ROS) in pancreatic cancer cells, contributing to autophagy activation and apoptosis induction. Furthermore, inhibiting TET-mediated autophagy leads to the accumulation of ROS in pancreatic cancer cells, significantly enhancing the pro-apoptotic effect of TET. Our findings demonstrate the anti-tumor role of TET in pancreatic cancer and support the effectiveness of combining autophagy inhibition with chemotherapy in cancer treatment. Nevertheless, further clinical experiments are required to substantiate these findings.

### Electronic supplementary material

Below is the link to the electronic supplementary material.

Supplementary Material 1

## Data Availability

No datasets were generated or analysed during the current study.

## Abbreviations

3-MA: 3-methyladenine
AKT: Protein kinase B
ATG7: Autophagy-related gene 7
AVO: Acidic vesicular organelles
Baf: A1-Bafilomycin A1
CQ: Chloroquine
DMEM: Dulbecco’s modified Eagle’s medium
DMSO: Dimethyl sulfoxide
ECL: Enhanced chemiluminescence
HCQ: Hydroxychloroquine
IHC: Immunohistochemical
mTOR: Mammalian target of rapamycin
MTT: 3-(4,5-dimethylthiazol-2-yl)-2,5-diphenyl tetrazolium bromide
NAC: N-acetylcysteine
PBS: Phosphate-buffered saline
ROS: Reactive oxygen species
TET: Tetrandrine
