# Supplementary material for: Inhibition of autophagy induced by tetrandrine promotes the accumulation of reactive oxygen species and sensitizes efficacy of tetrandrine in pancreatic cancer
Source: Cancer Cell Int. 2024 Jul 10;24:241. doi: 10.1186/s12935-024-03410-5 (PMC11238362; doi:10.1186/s12935-024-03410-5)
Supplement: Supplementary file 1 — Supplementary Material 1 [file 12935_2024_3410_MOESM1_ESM.pptx]

## Slide 1
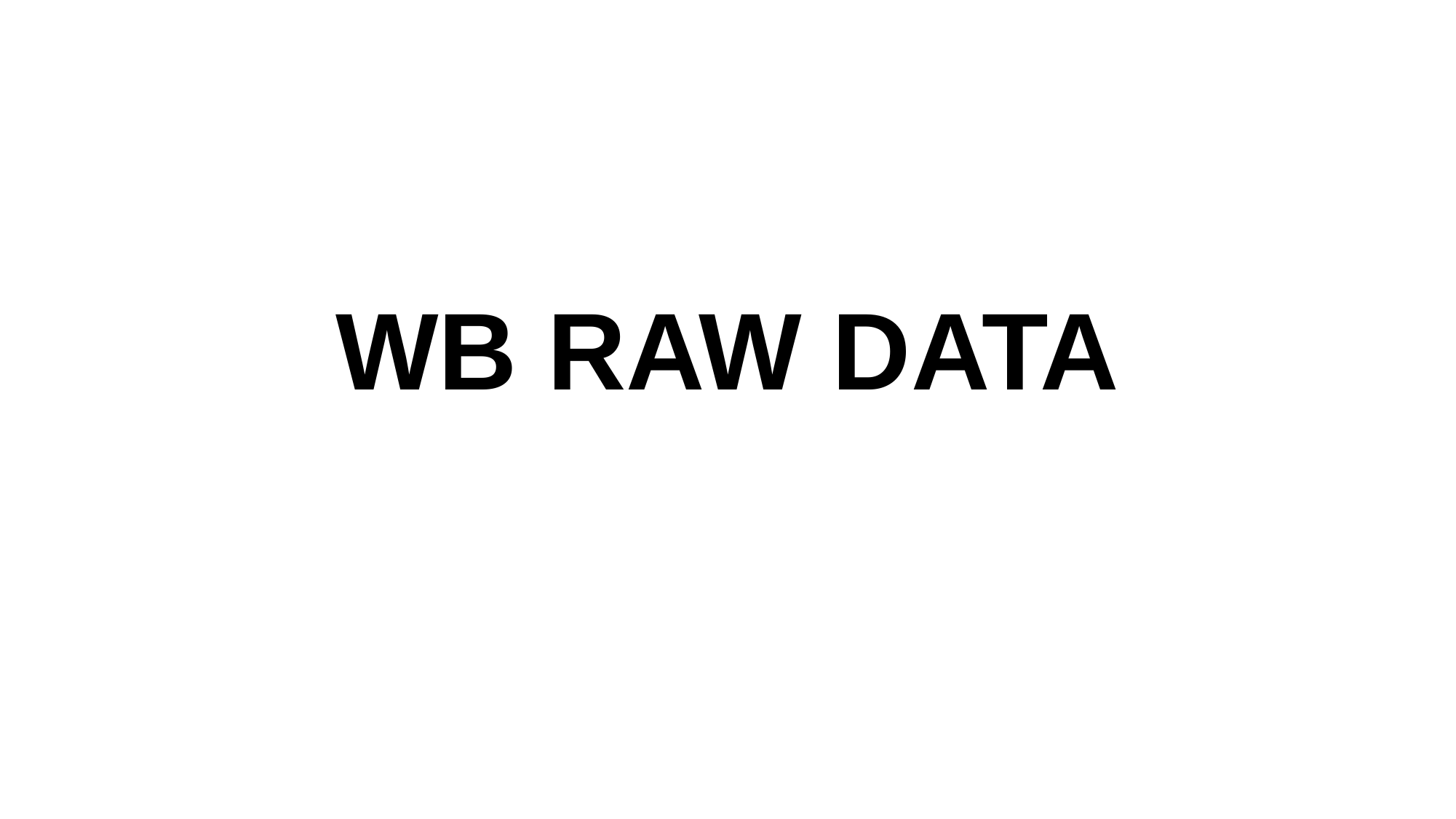

# WB RAW DATA

## Slide 2
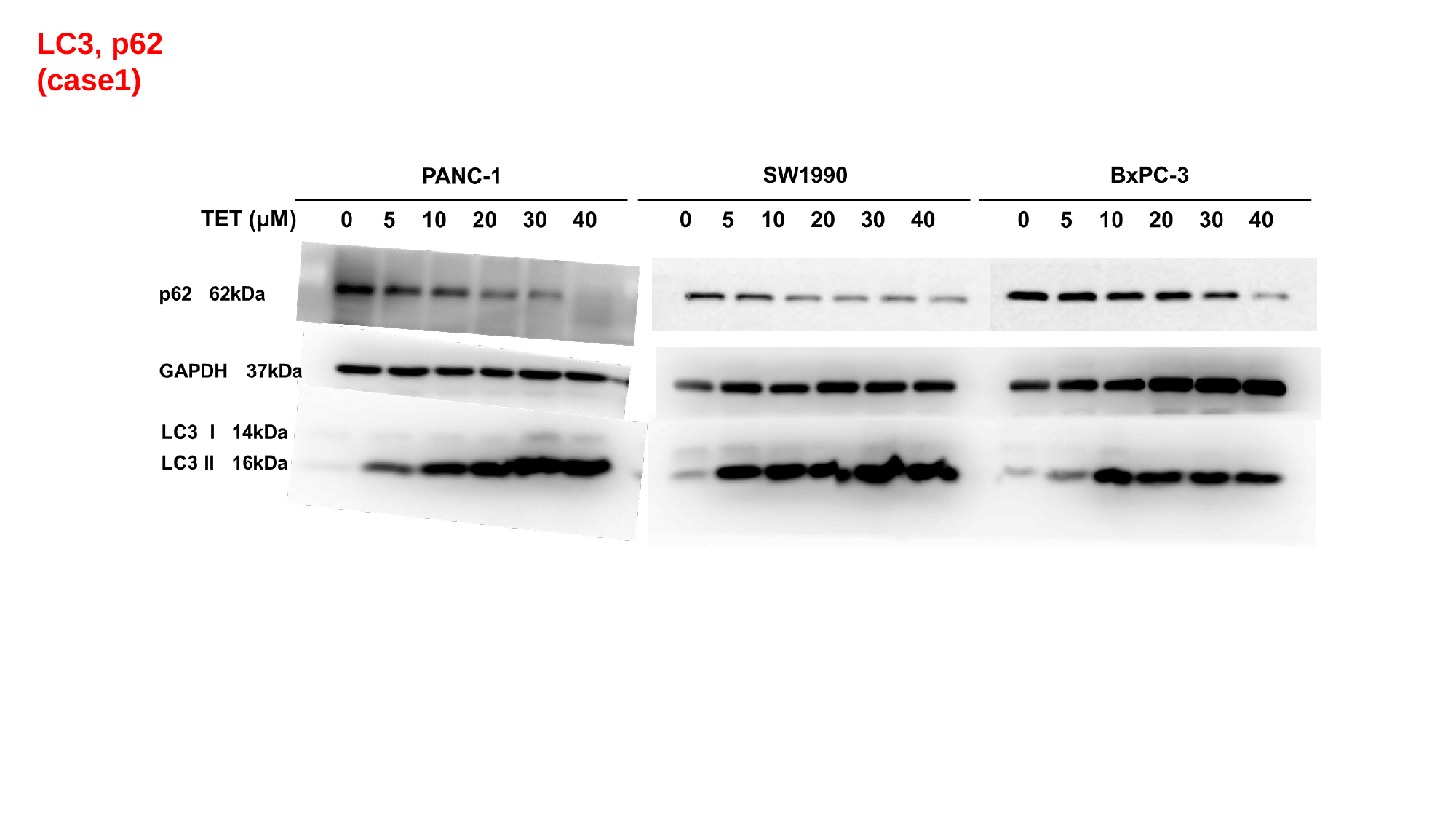

LC3, p62 (case1)

## Slide 3
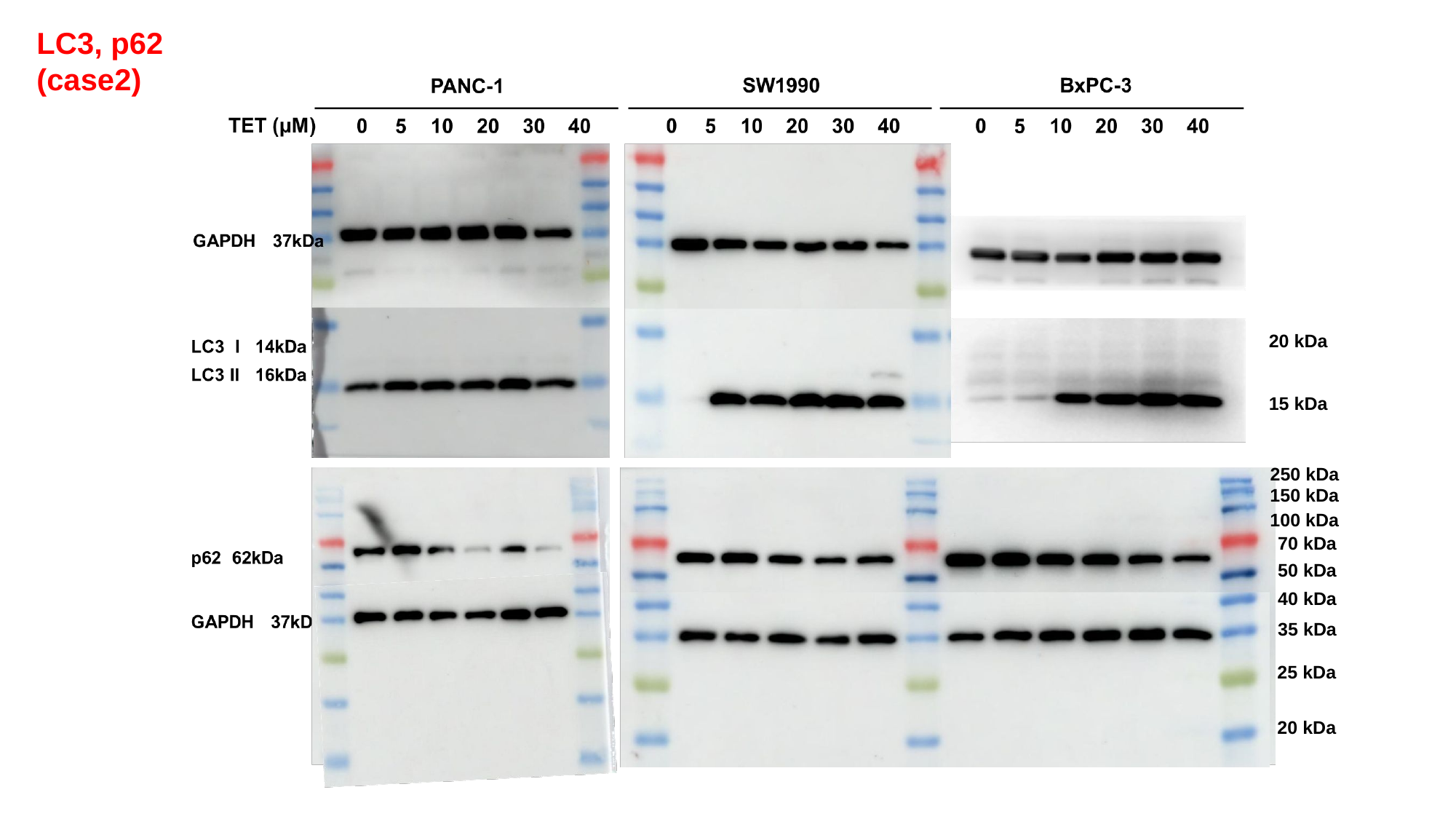

LC3, p62 (case2)
20 kDa
15 kDa
250 kDa
150 kDa
100 kDa
70 kDa
50 kDa
40 kDa
35 kDa
25 kDa
20 kDa

## Slide 4
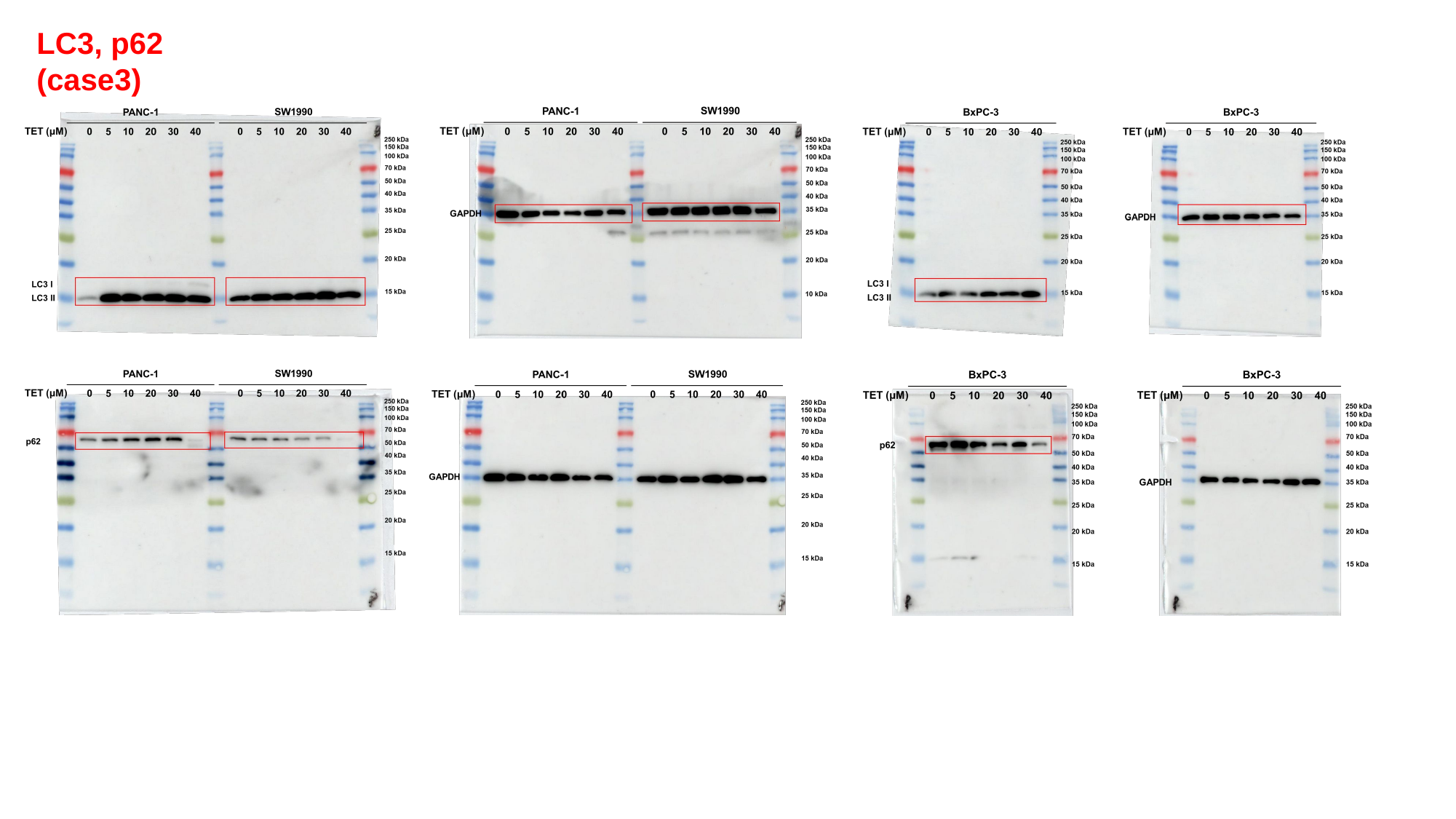

LC3, p62 (case3)

## Slide 5
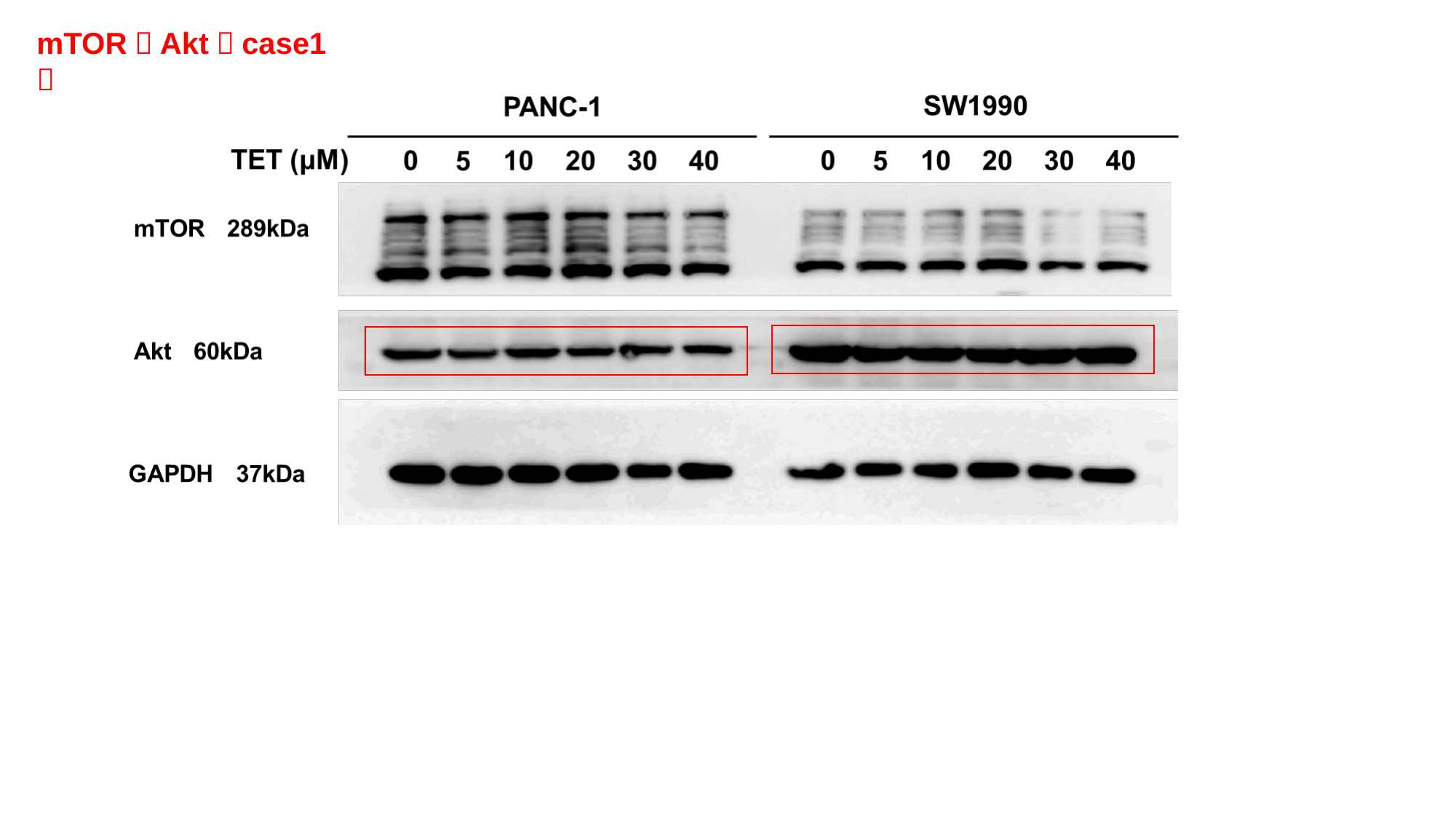

mTOR，Akt（case1）

## Slide 6
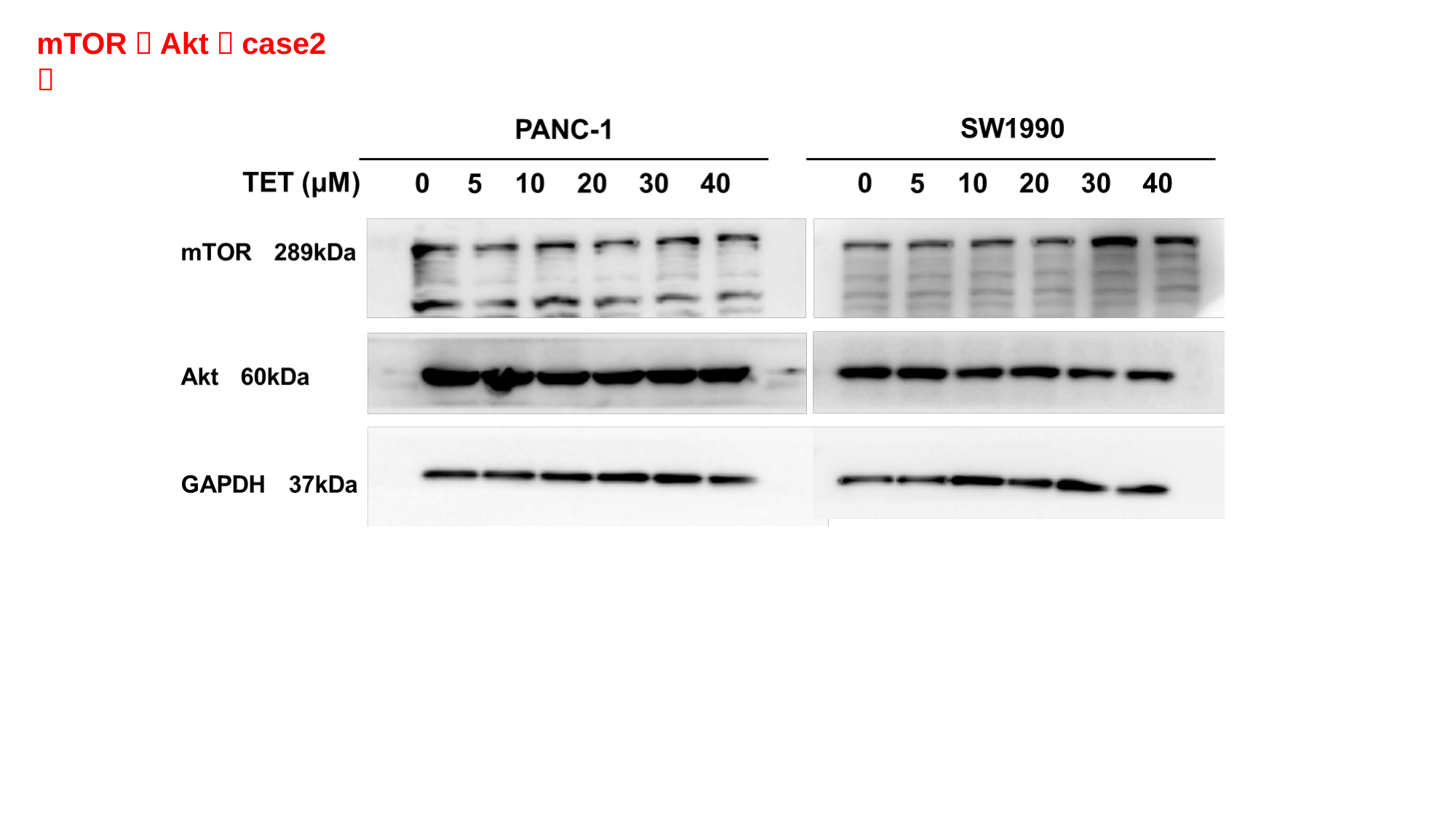

mTOR，Akt（case2）

## Slide 7
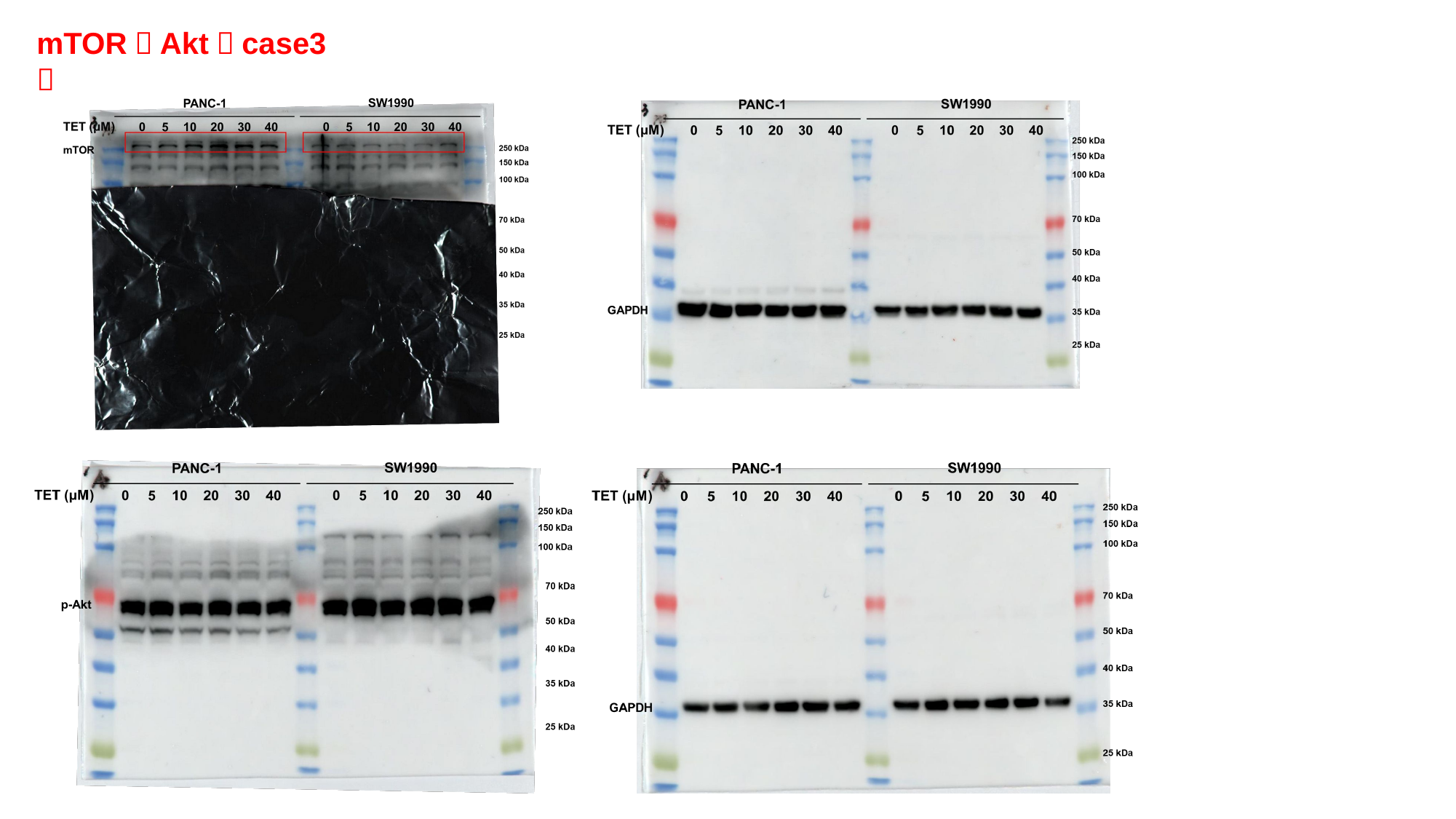

mTOR，Akt（case3）

## Slide 8
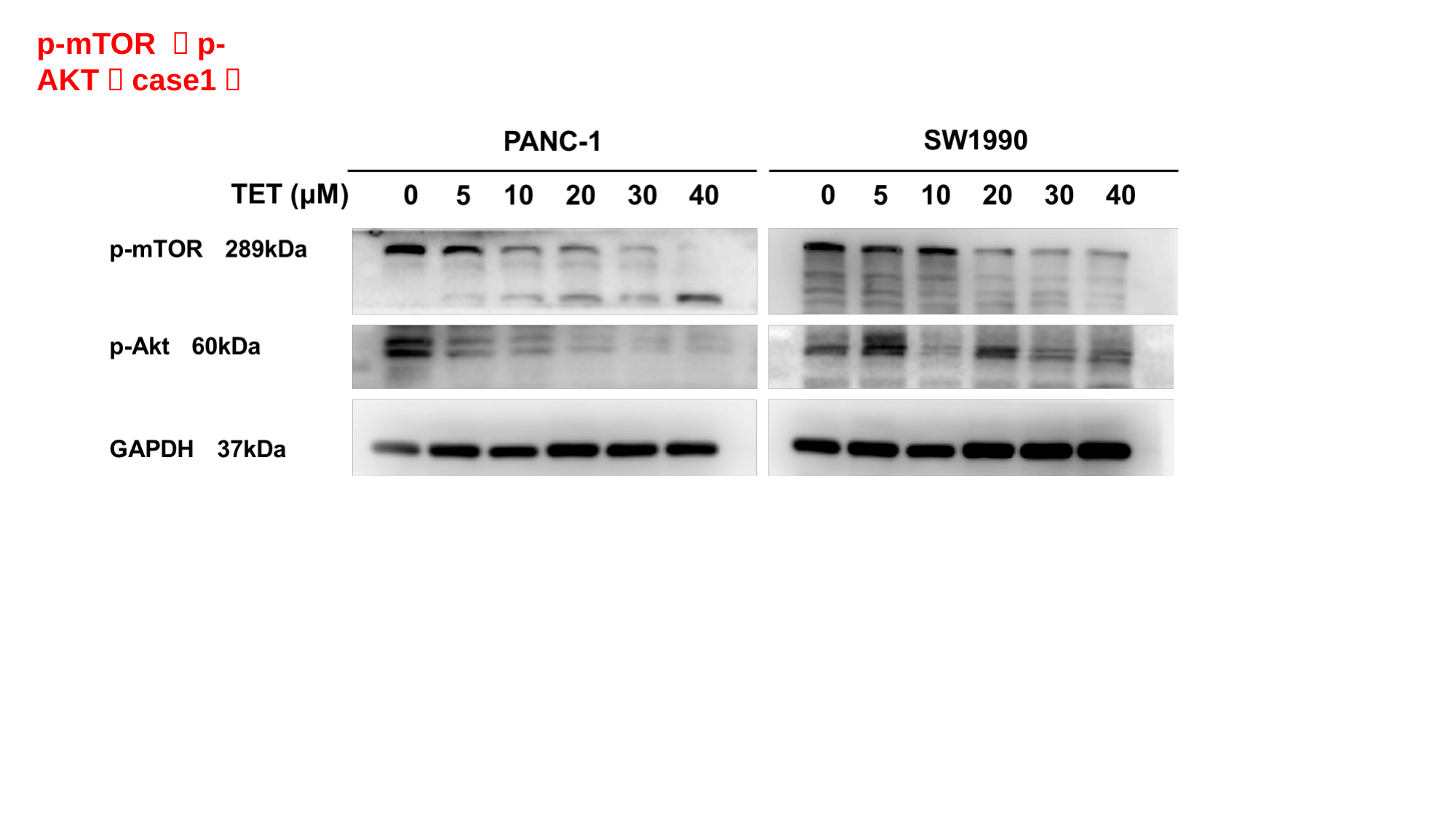

p-mTOR ，p-AKT（case1）

## Slide 9
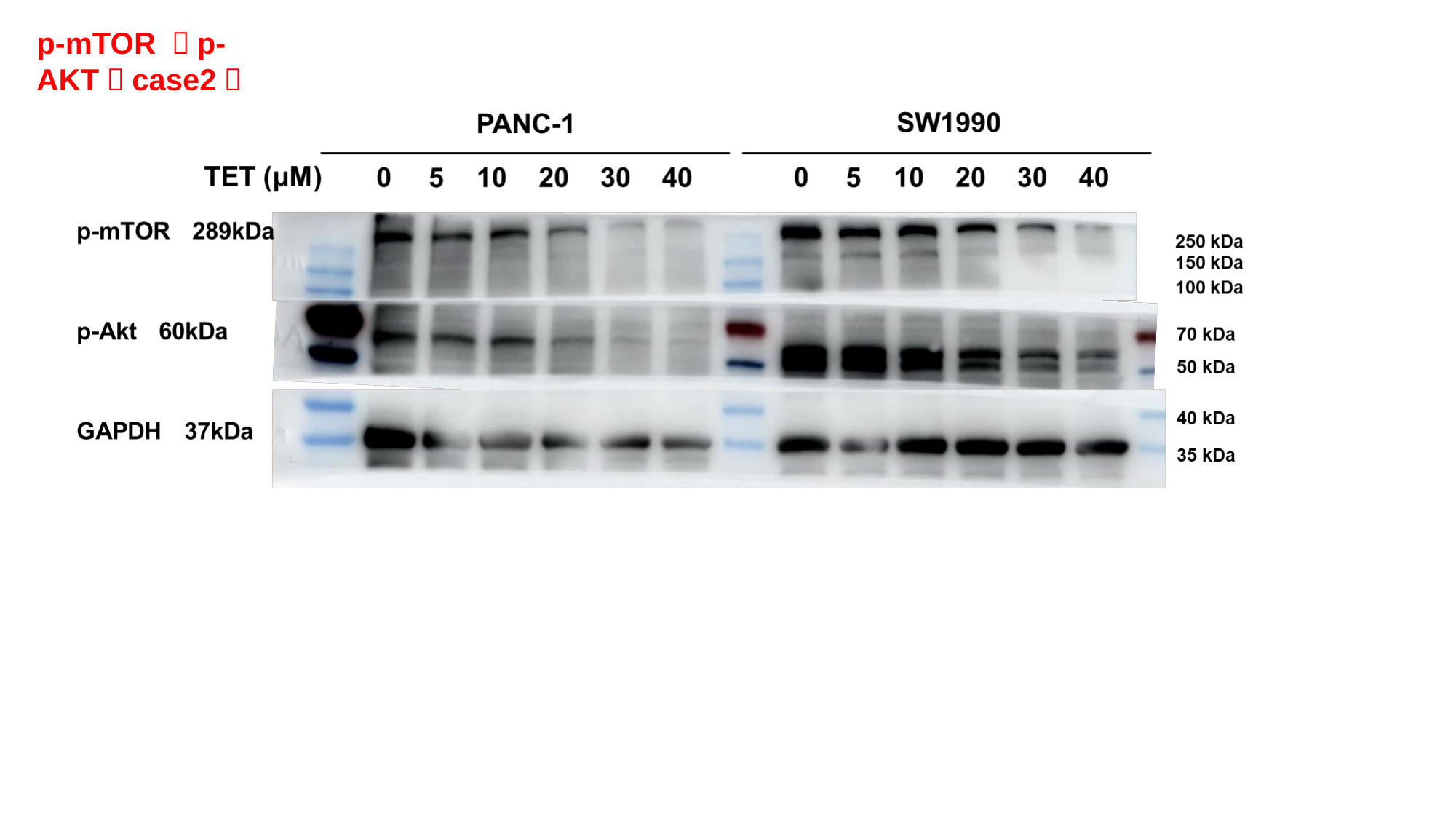

p-mTOR ，p-AKT（case2）

## Slide 10
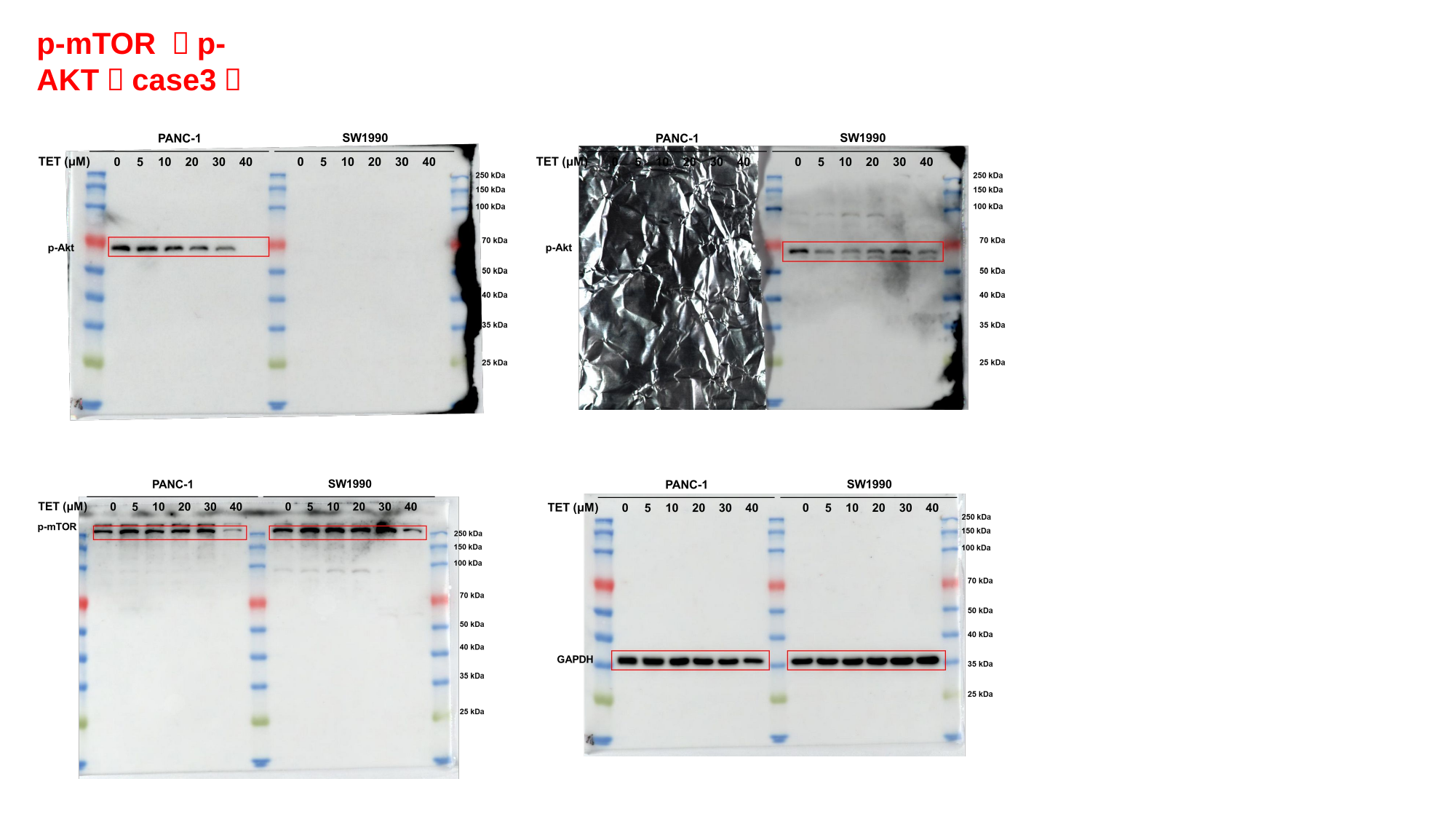

p-mTOR ，p-AKT（case3）

## Slide 11
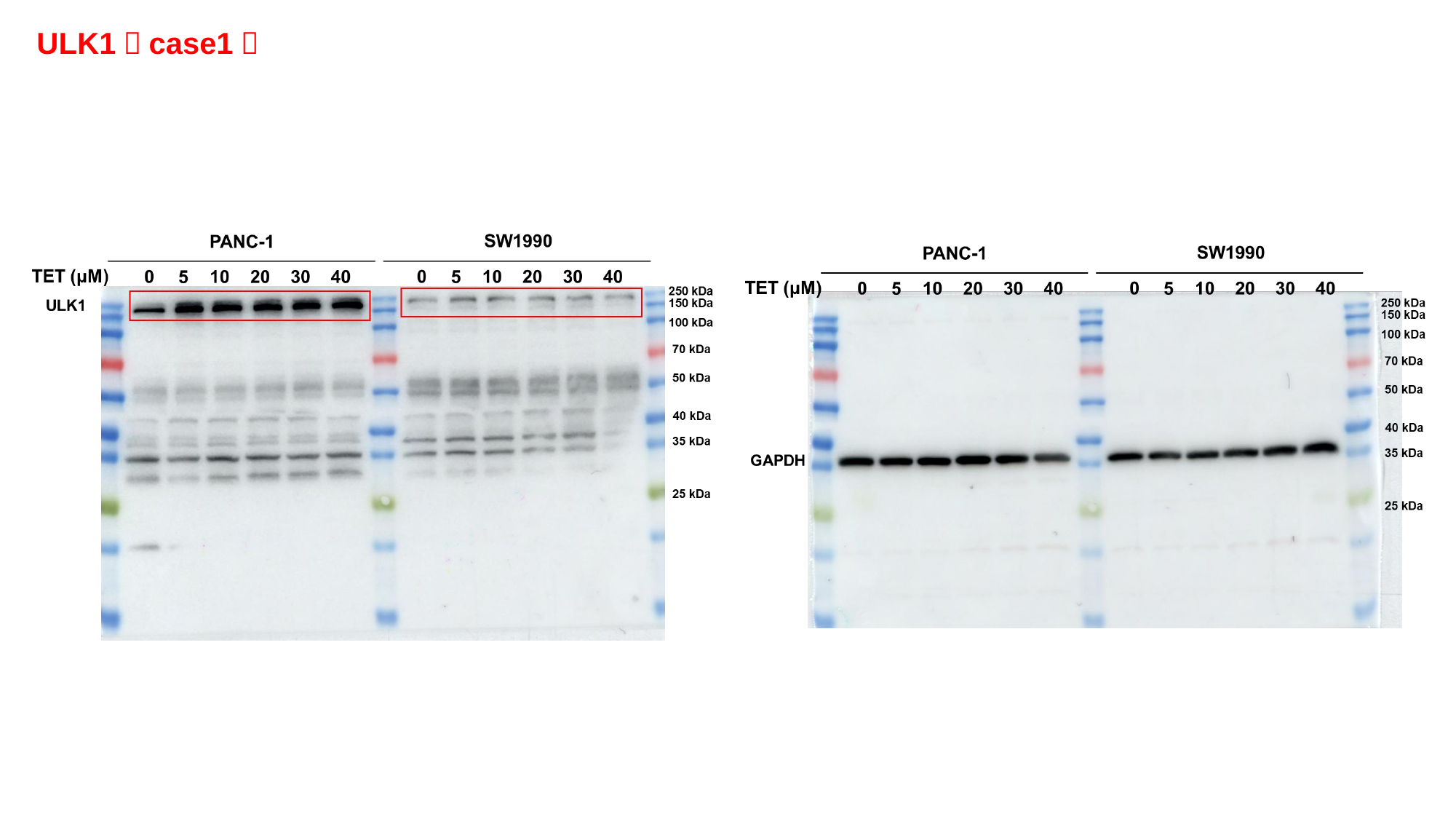

ULK1（case1）

## Slide 12
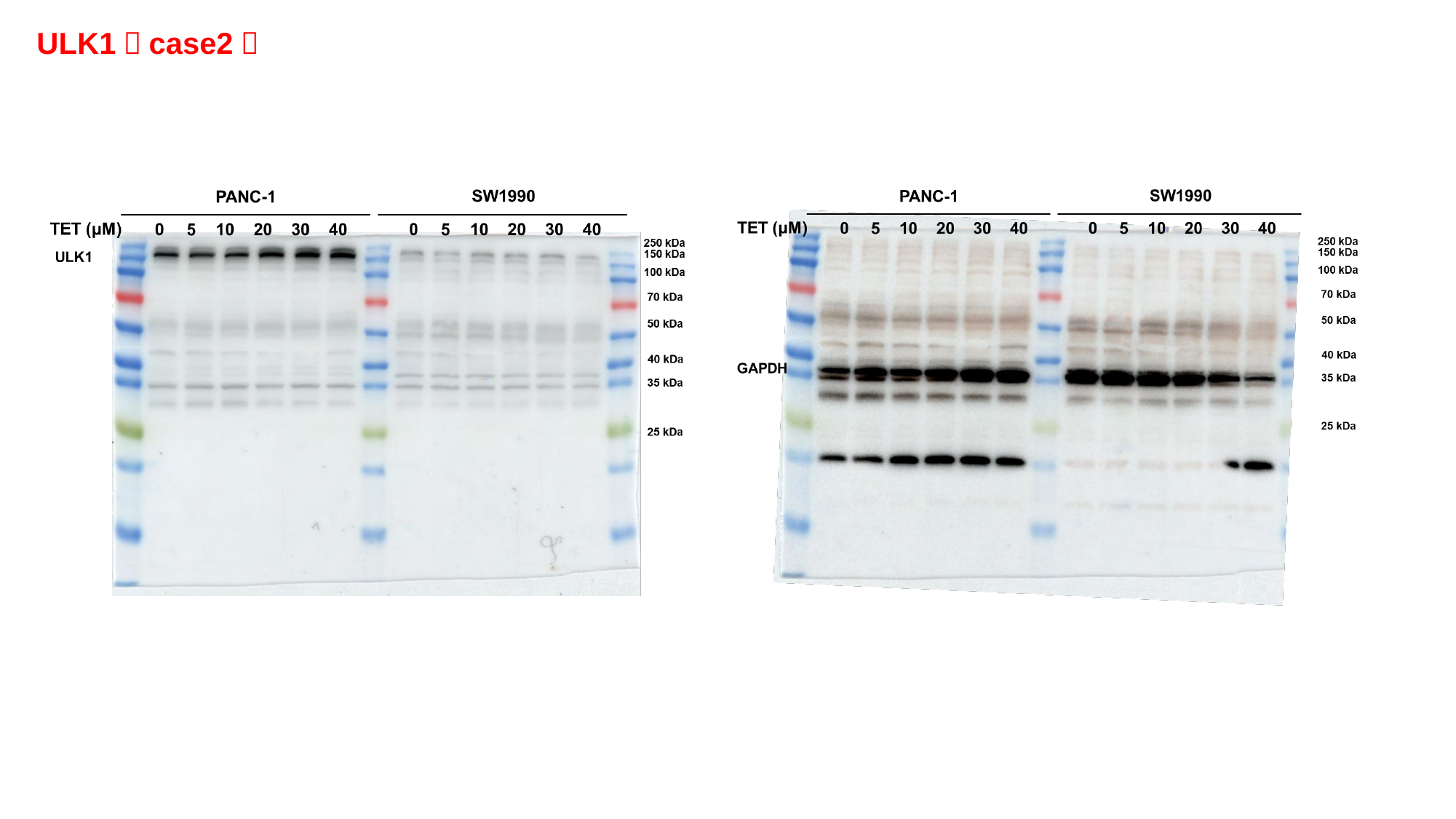

ULK1（case2）

## Slide 13
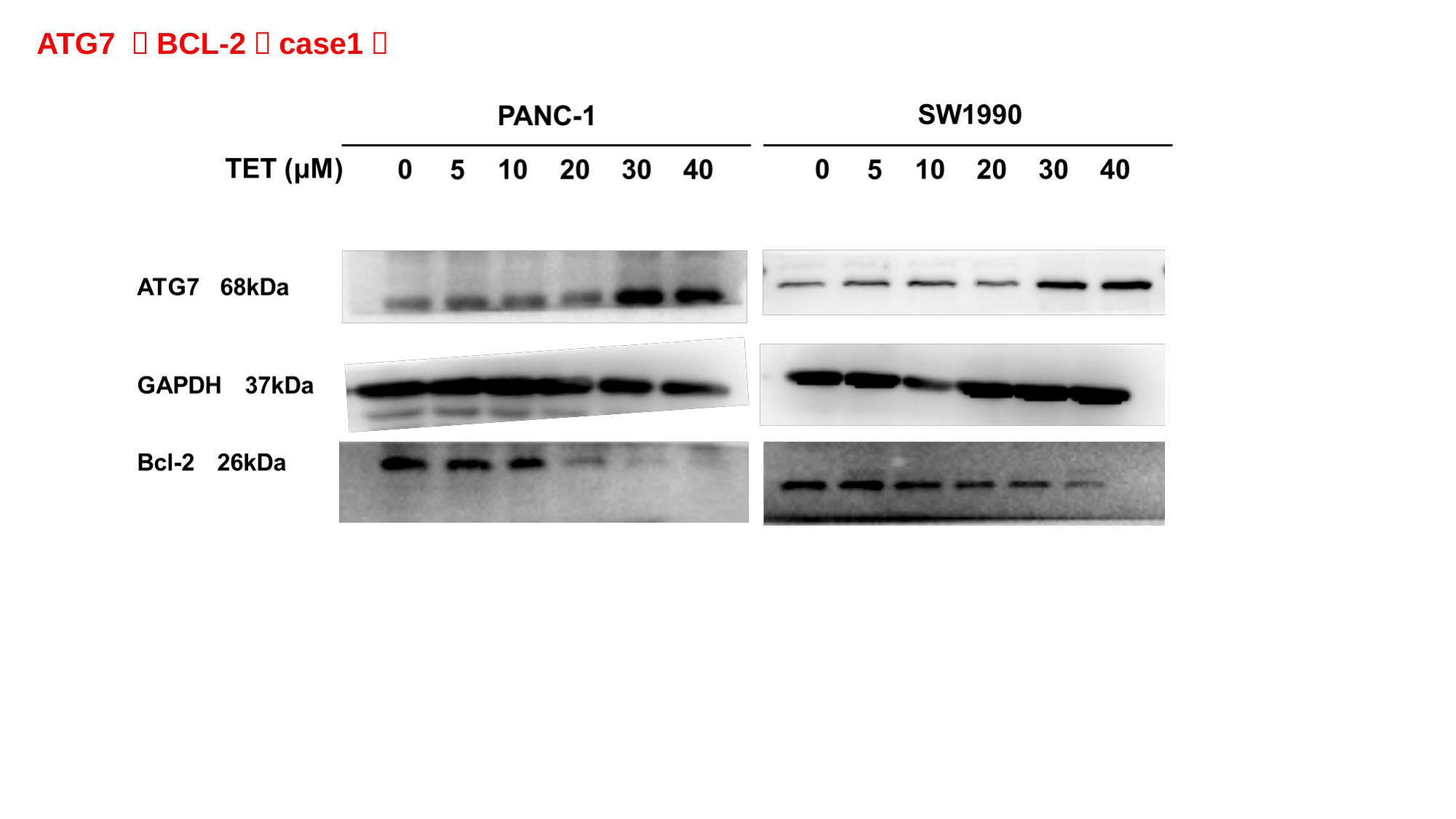

ATG7 ，BCL-2（case1）

## Slide 14
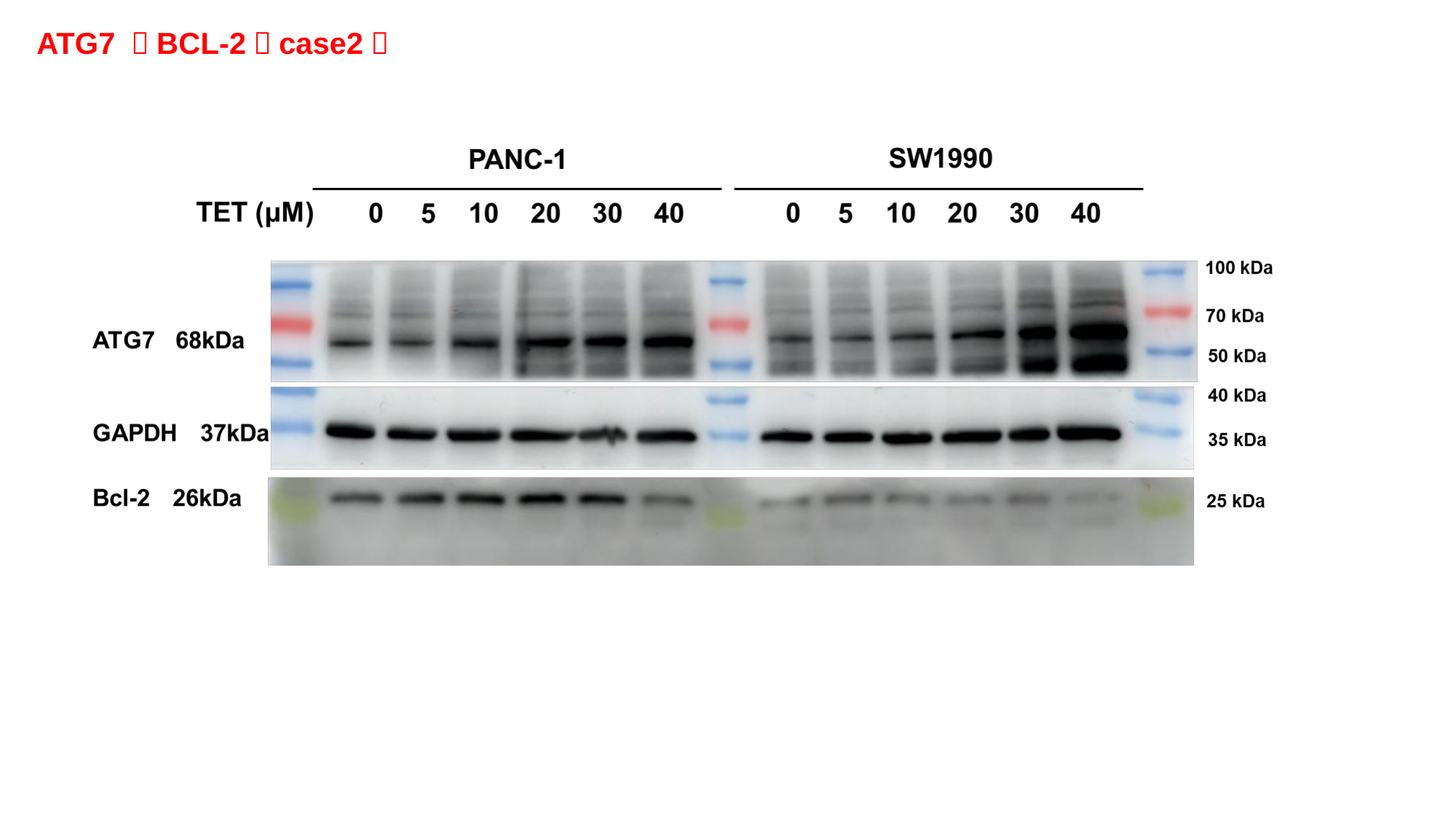

ATG7 ，BCL-2（case2）

## Slide 15
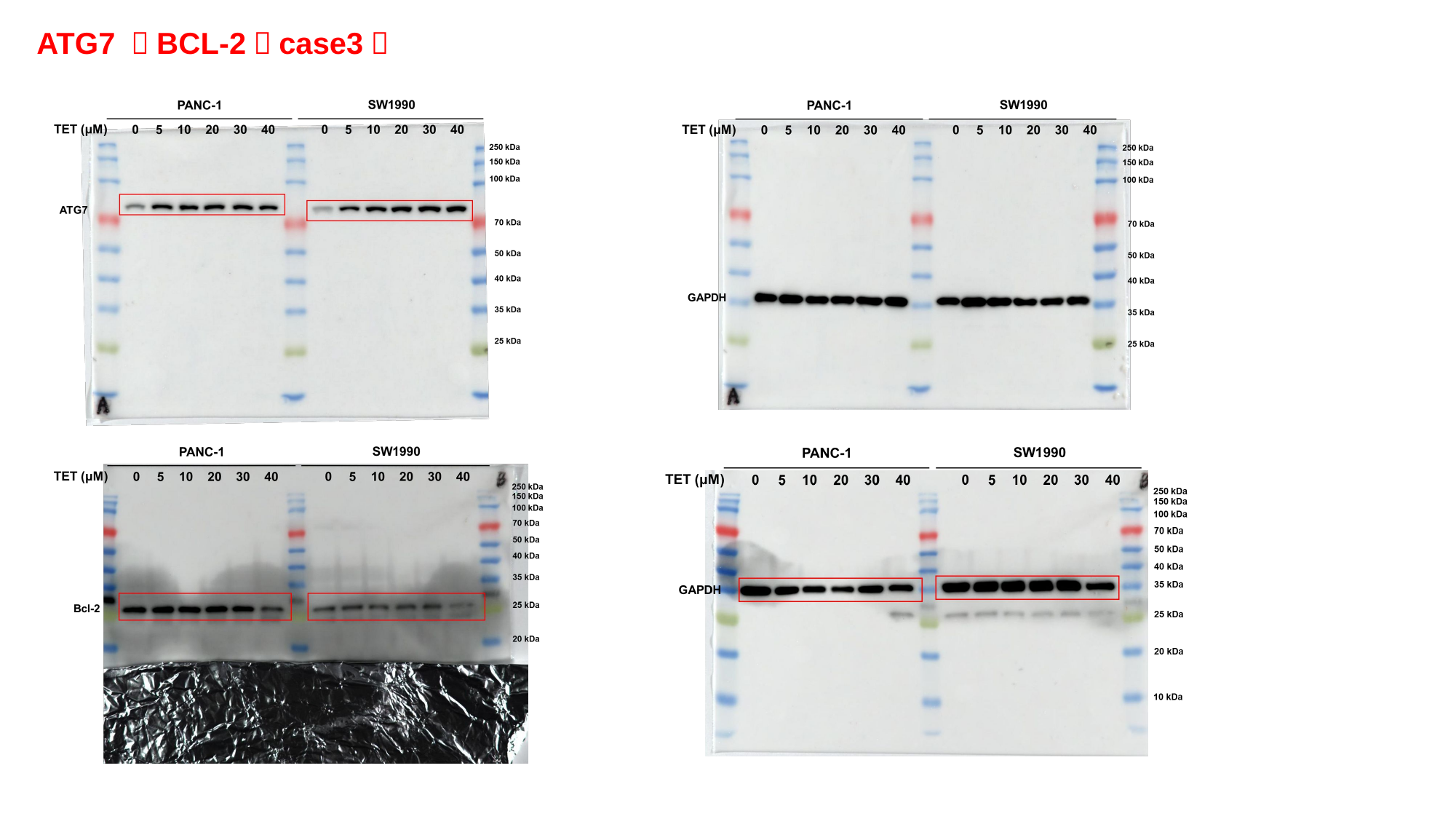

ATG7 ，BCL-2（case3）

## Slide 16
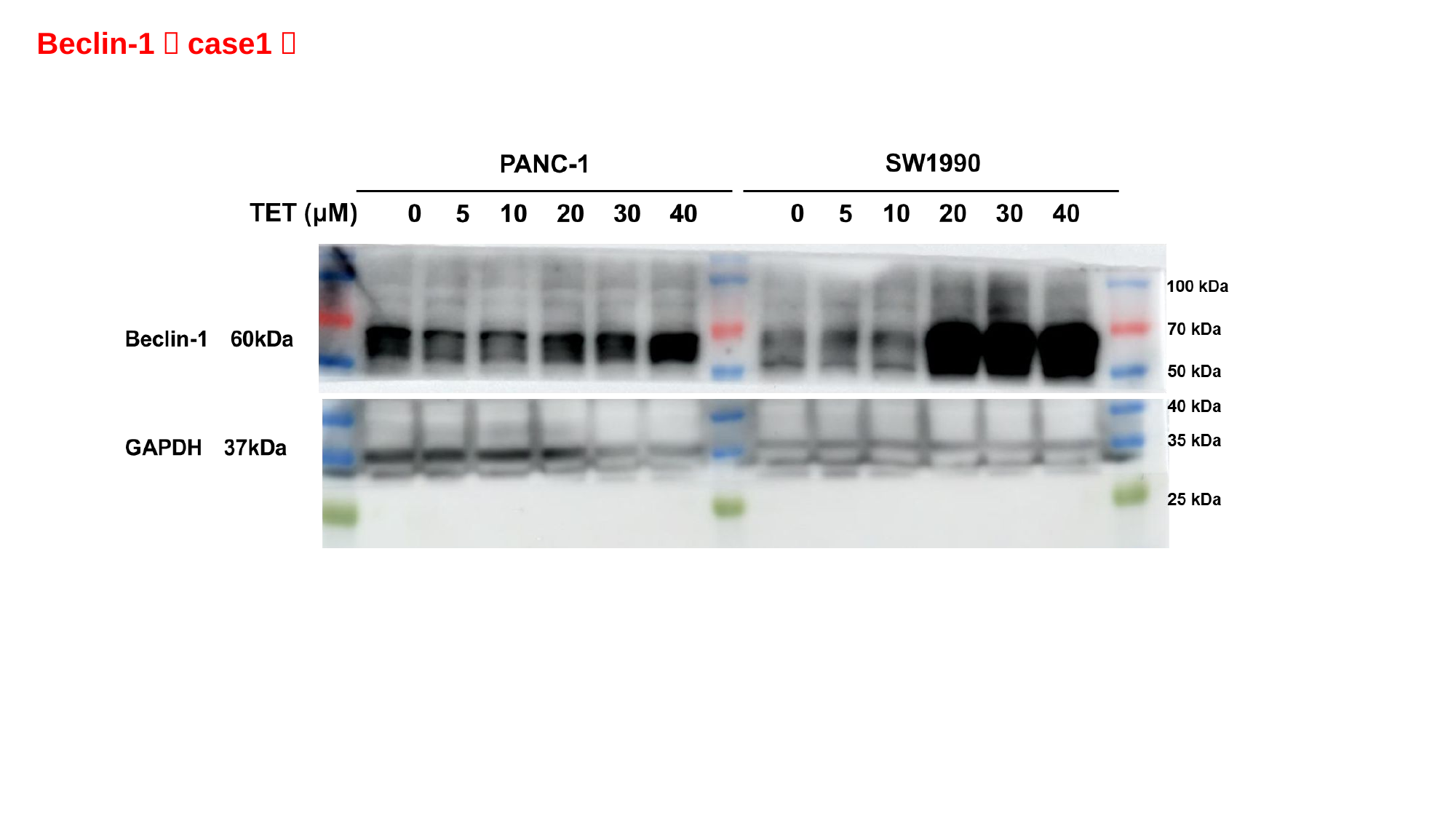

Beclin-1（case1）

## Slide 17
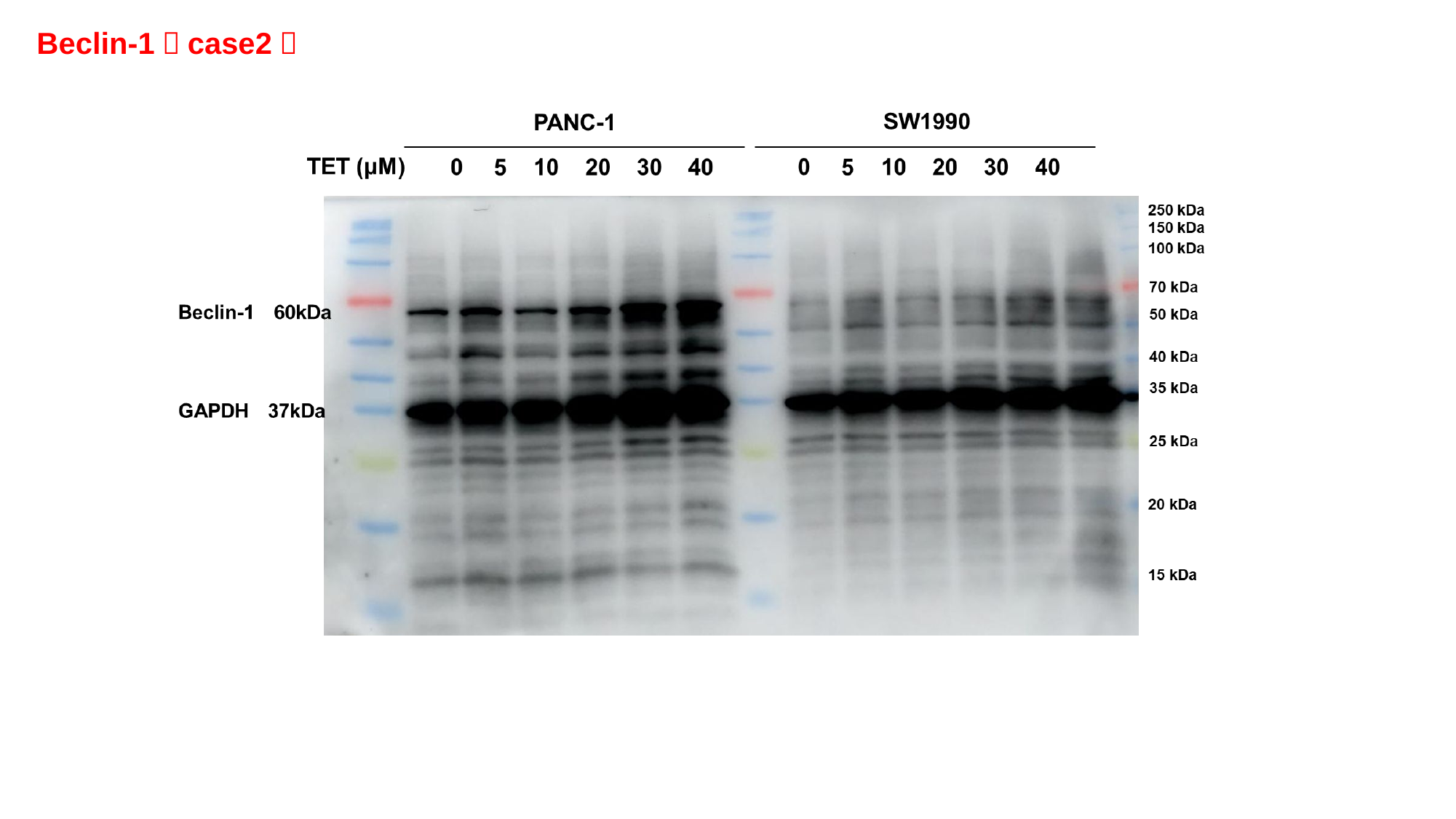

Beclin-1（case2）

## Slide 18
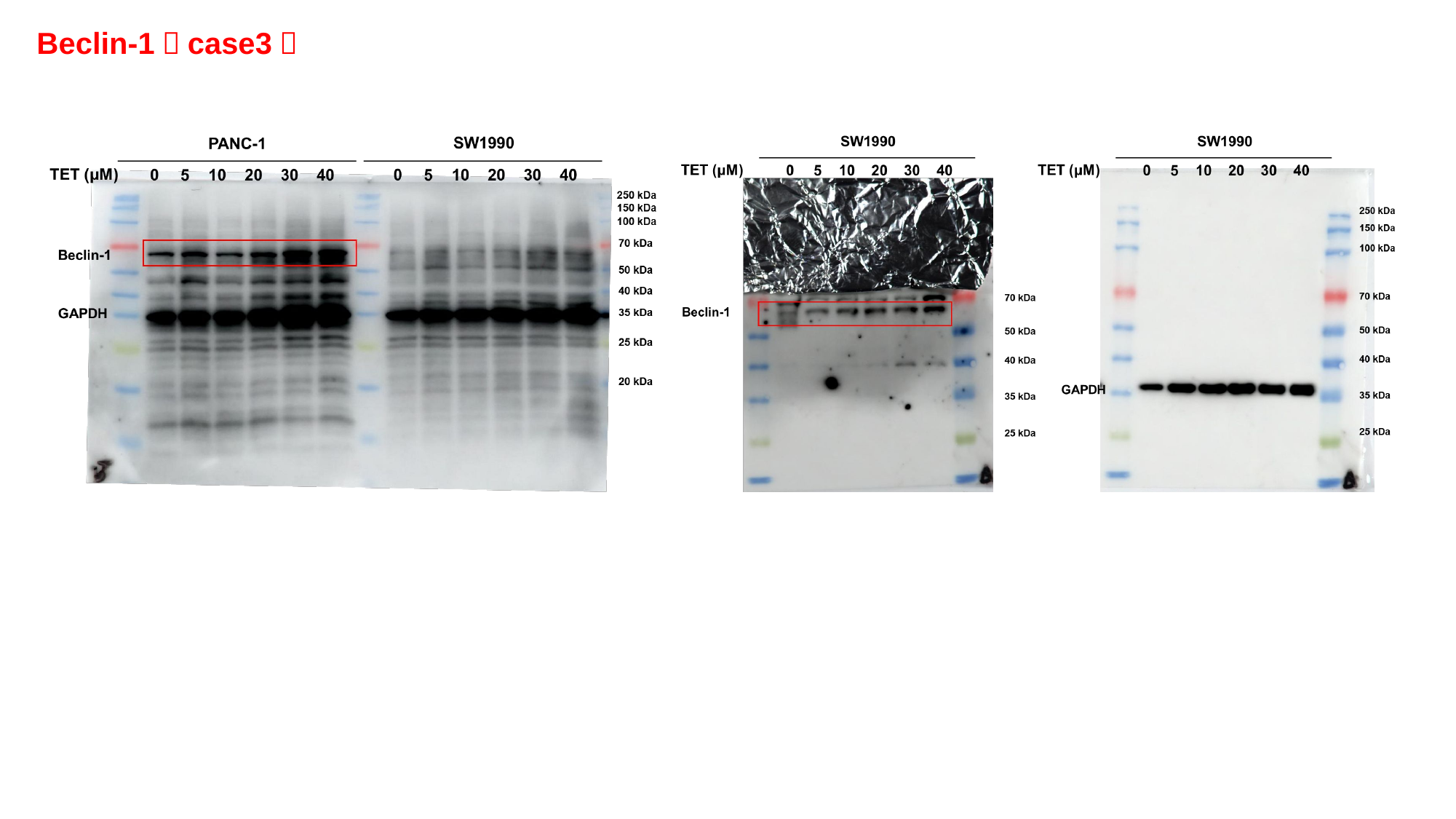

Beclin-1（case3）

## Slide 19
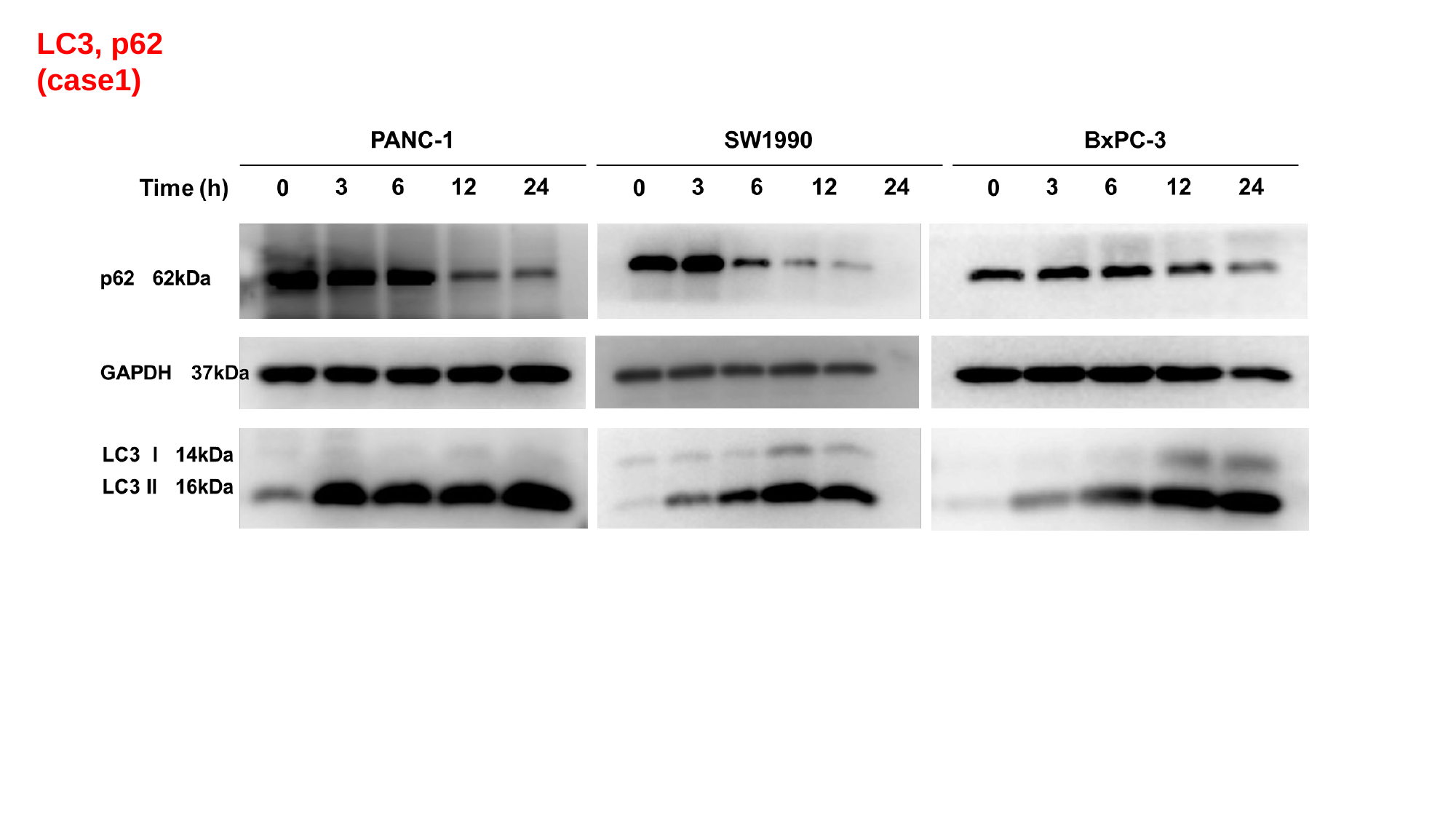

LC3, p62 (case1)

## Slide 20
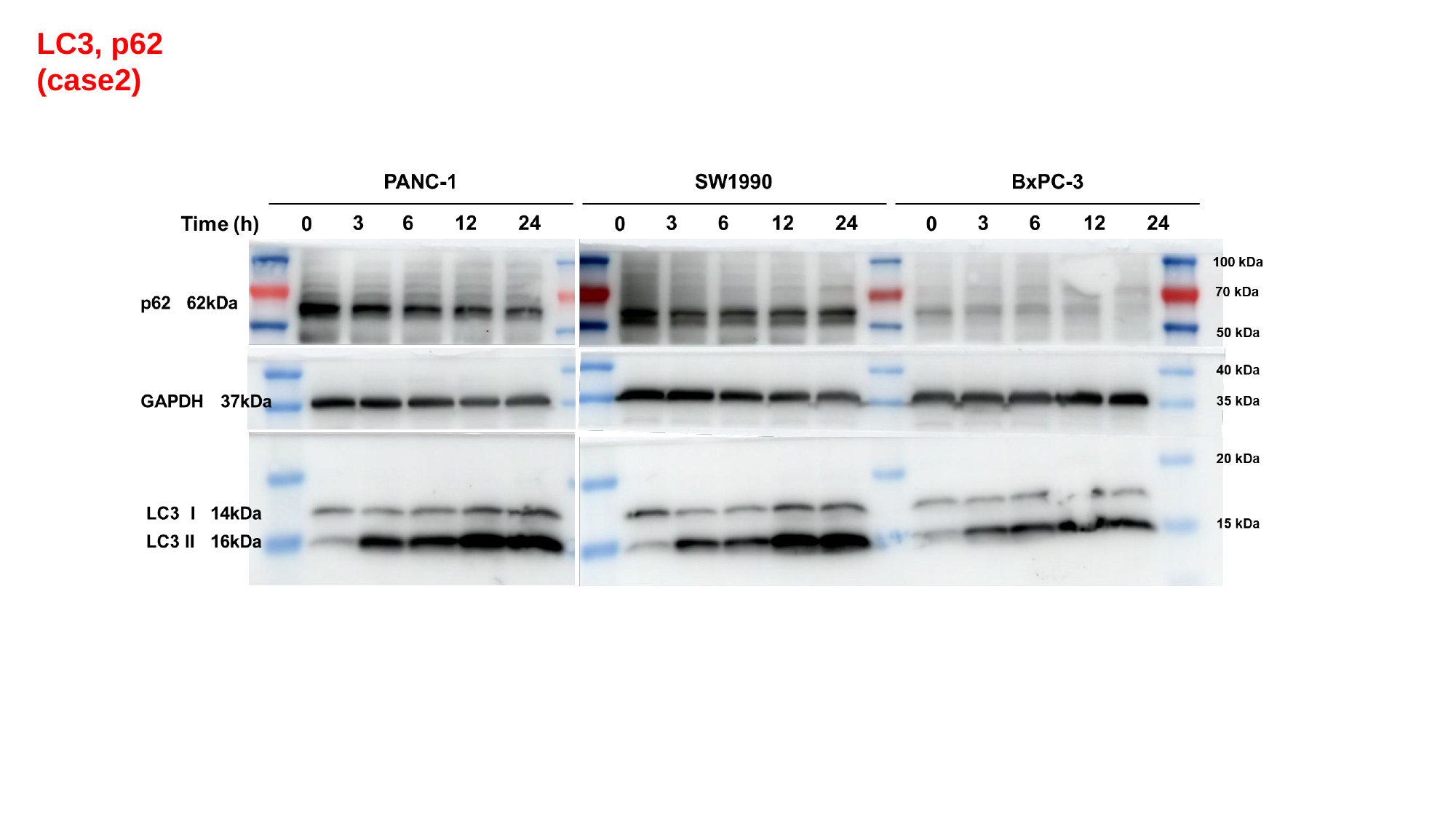

LC3, p62 (case2)

## Slide 21
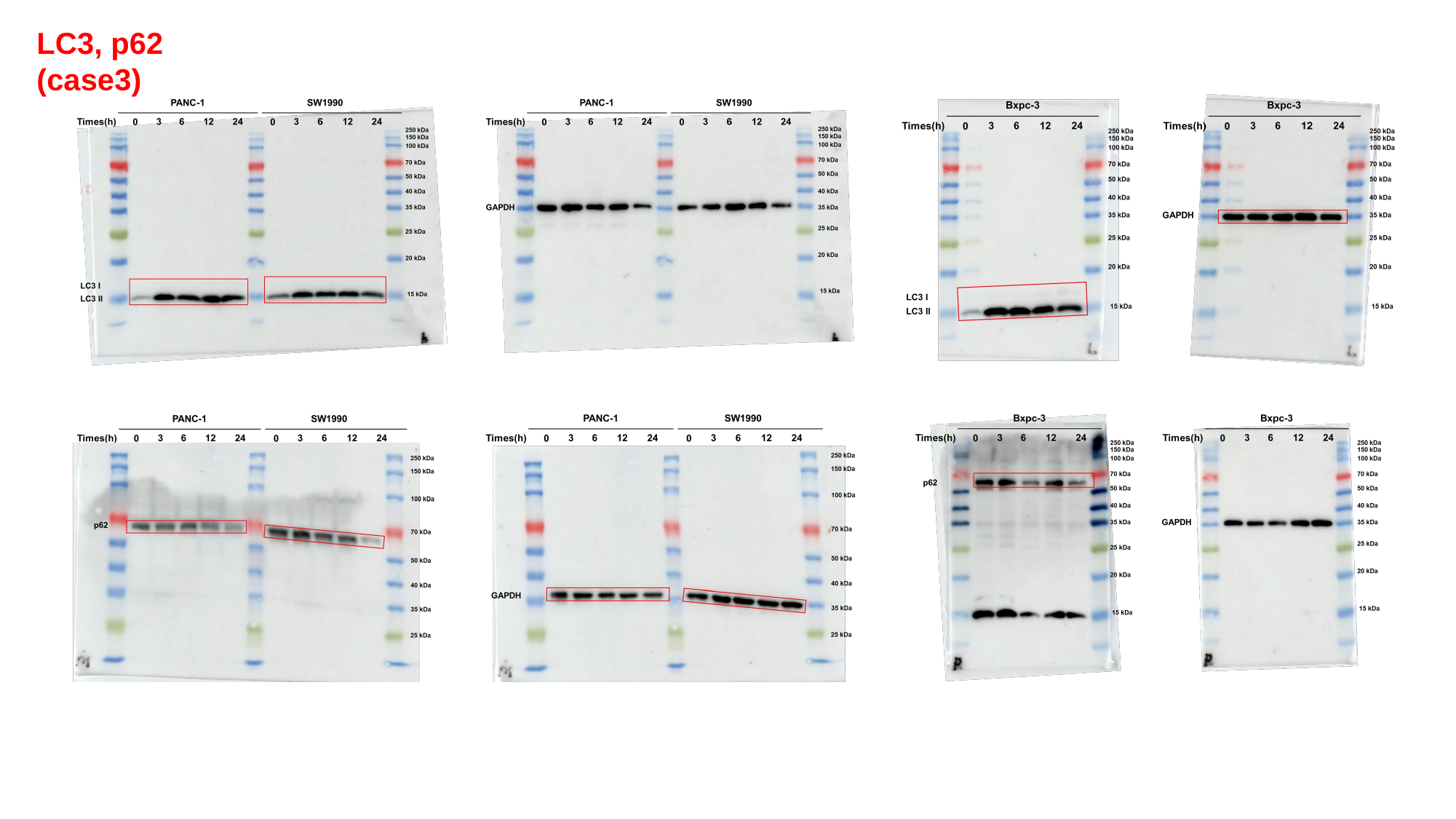

LC3, p62 (case3)

## Slide 22
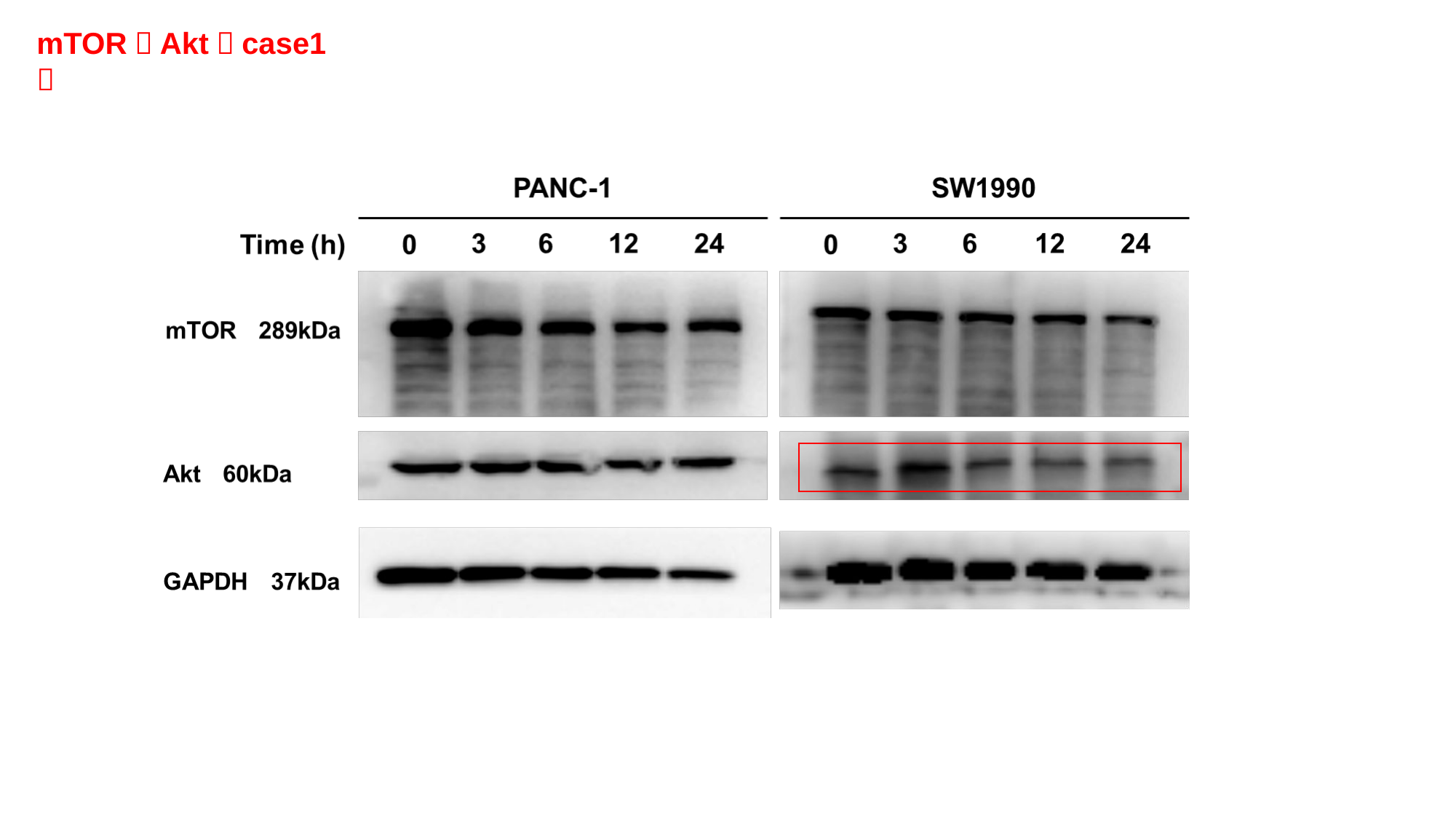

mTOR，Akt（case1）

## Slide 23
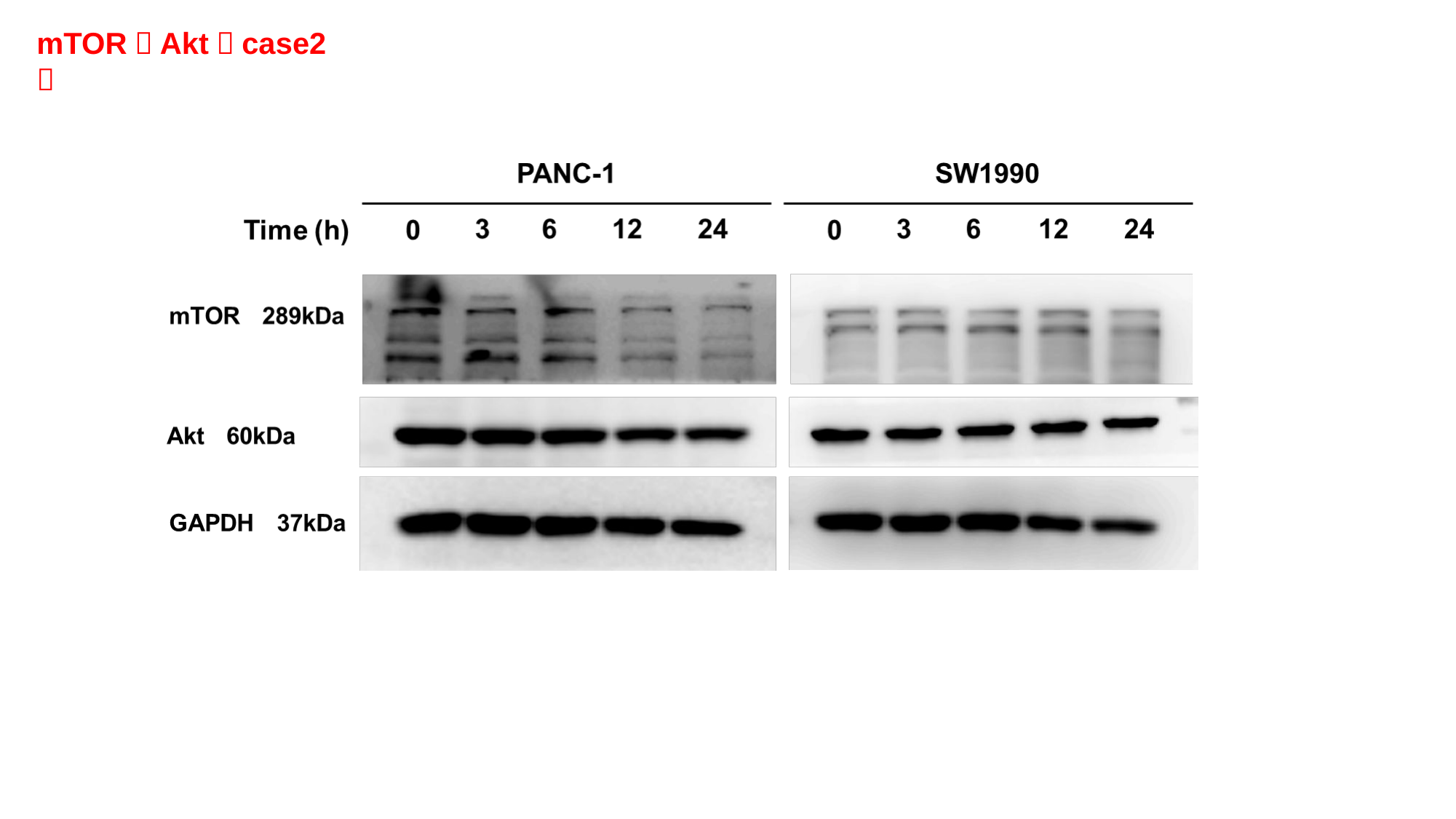

mTOR，Akt（case2）

## Slide 24
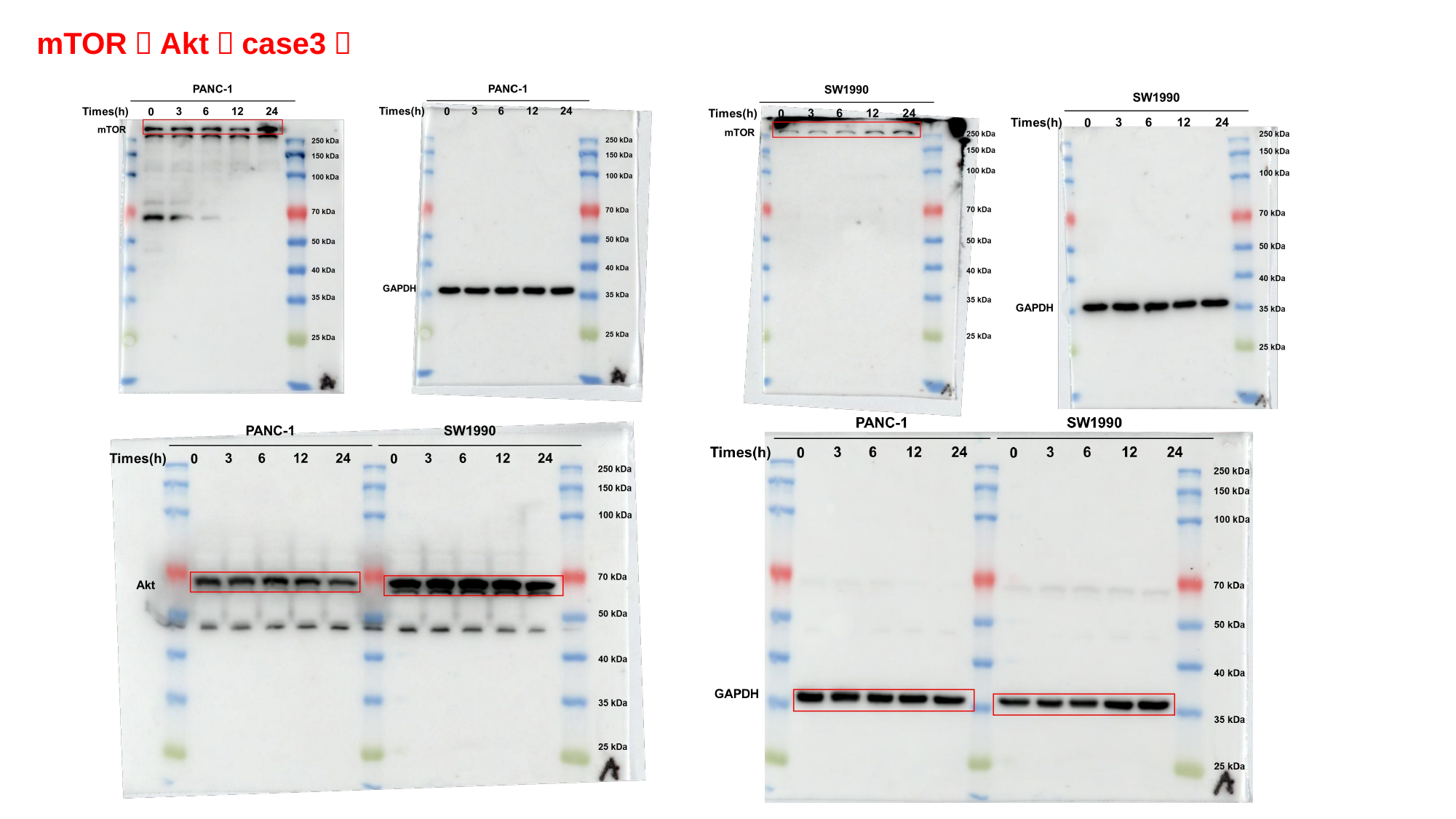

mTOR，Akt（case3）

## Slide 25
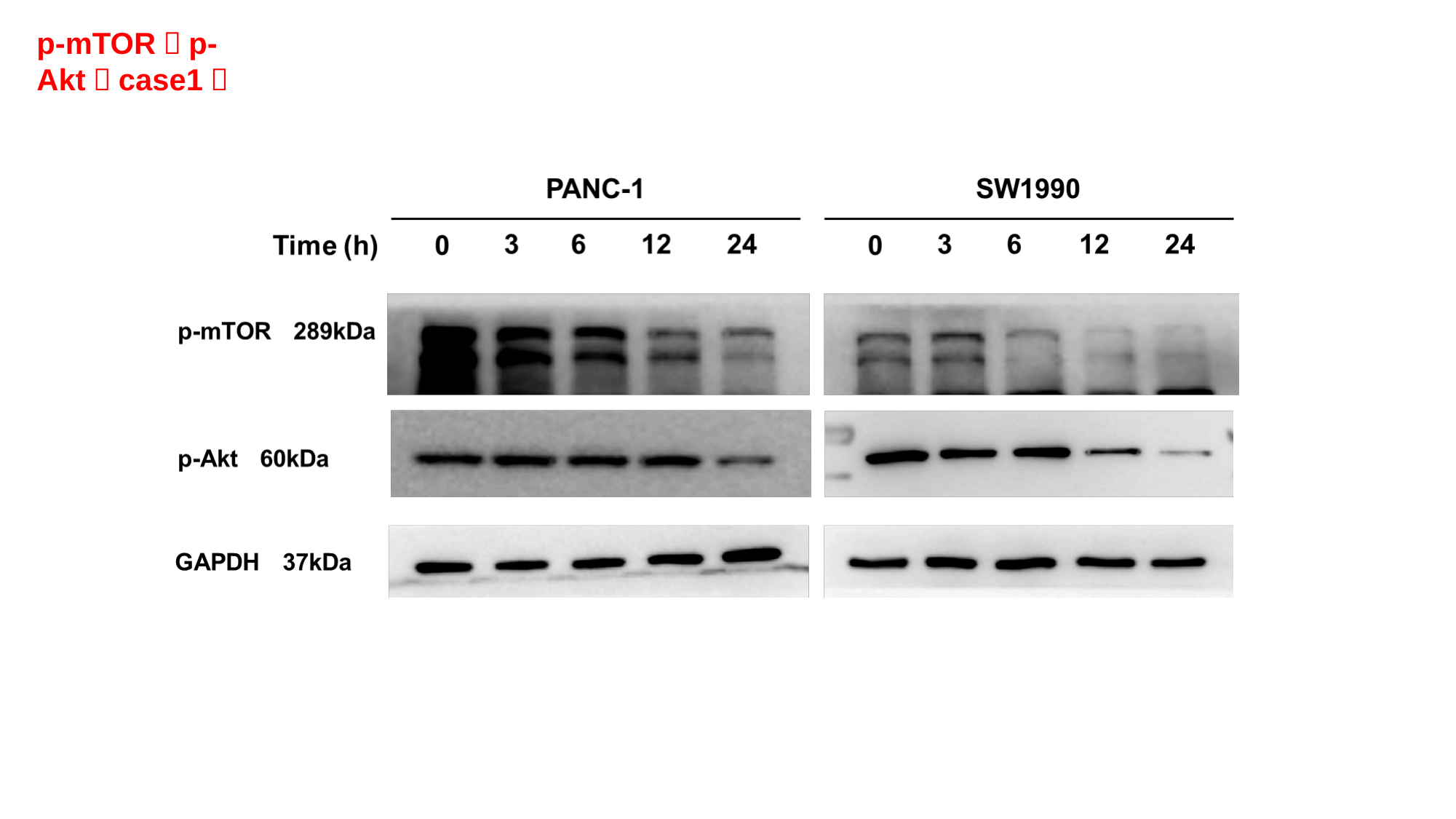

p-mTOR，p-Akt（case1）

## Slide 26
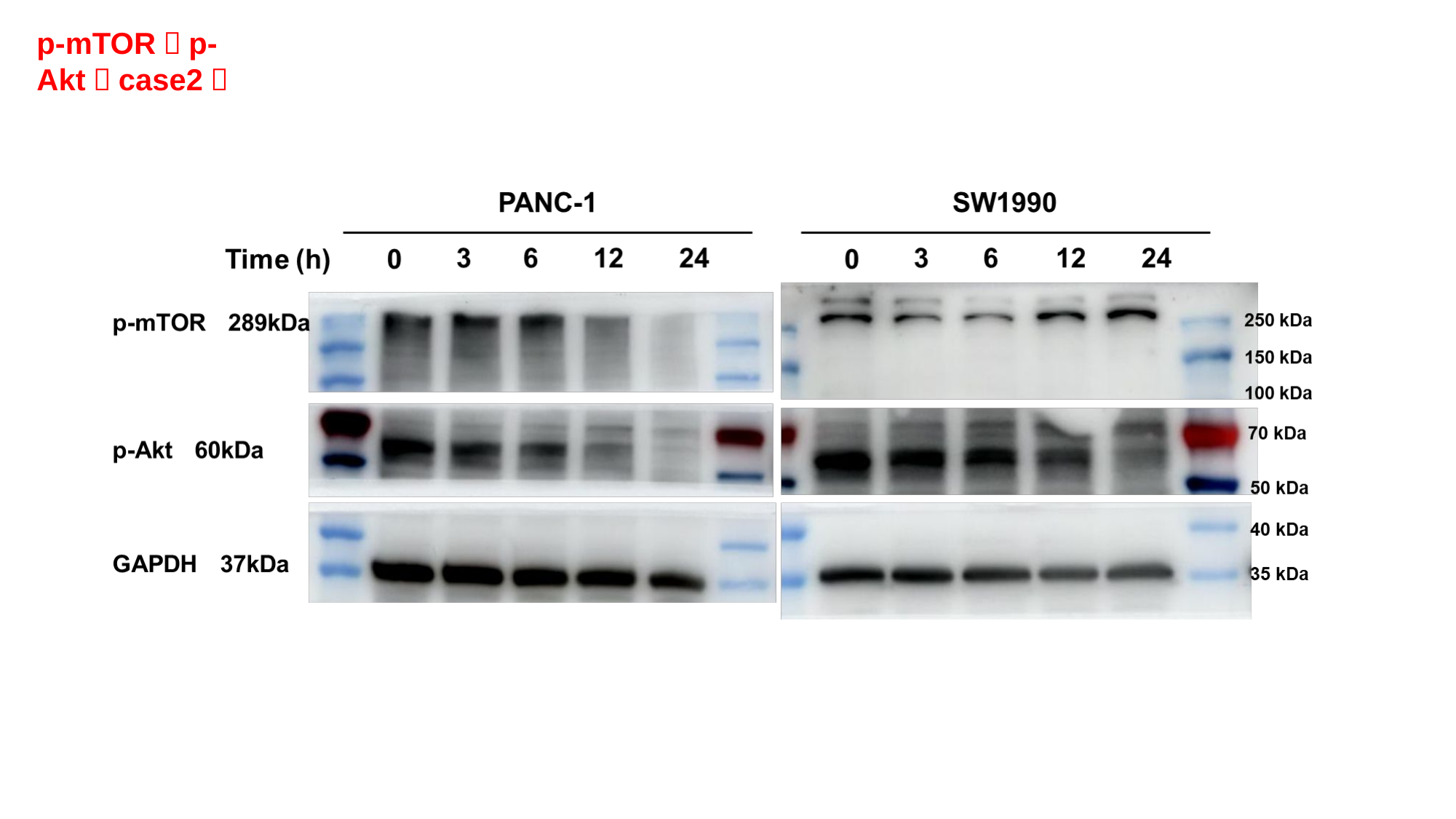

p-mTOR，p-Akt（case2）

## Slide 27
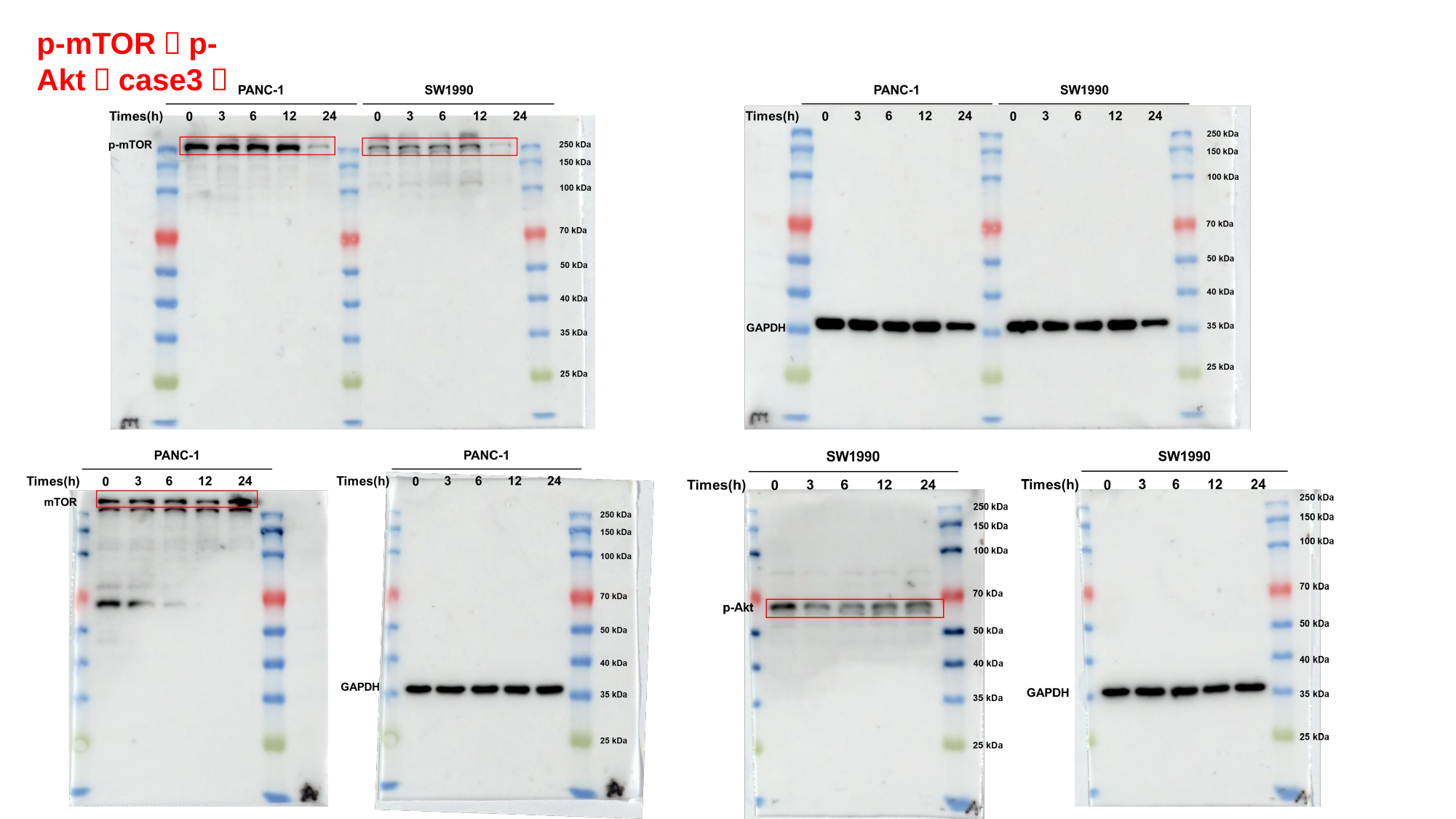

p-mTOR，p-Akt（case3）

## Slide 28
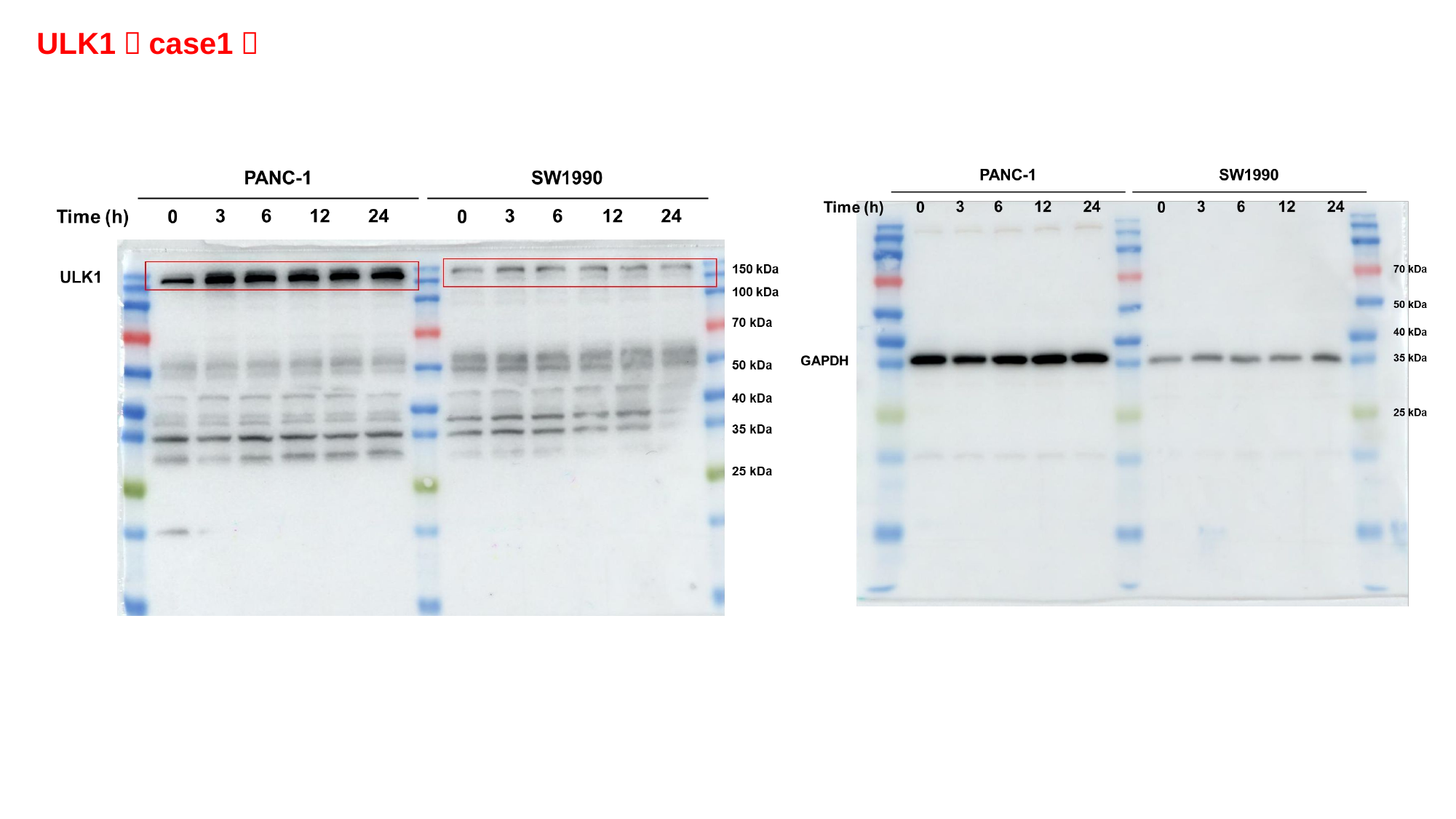

ULK1（case1）

## Slide 29
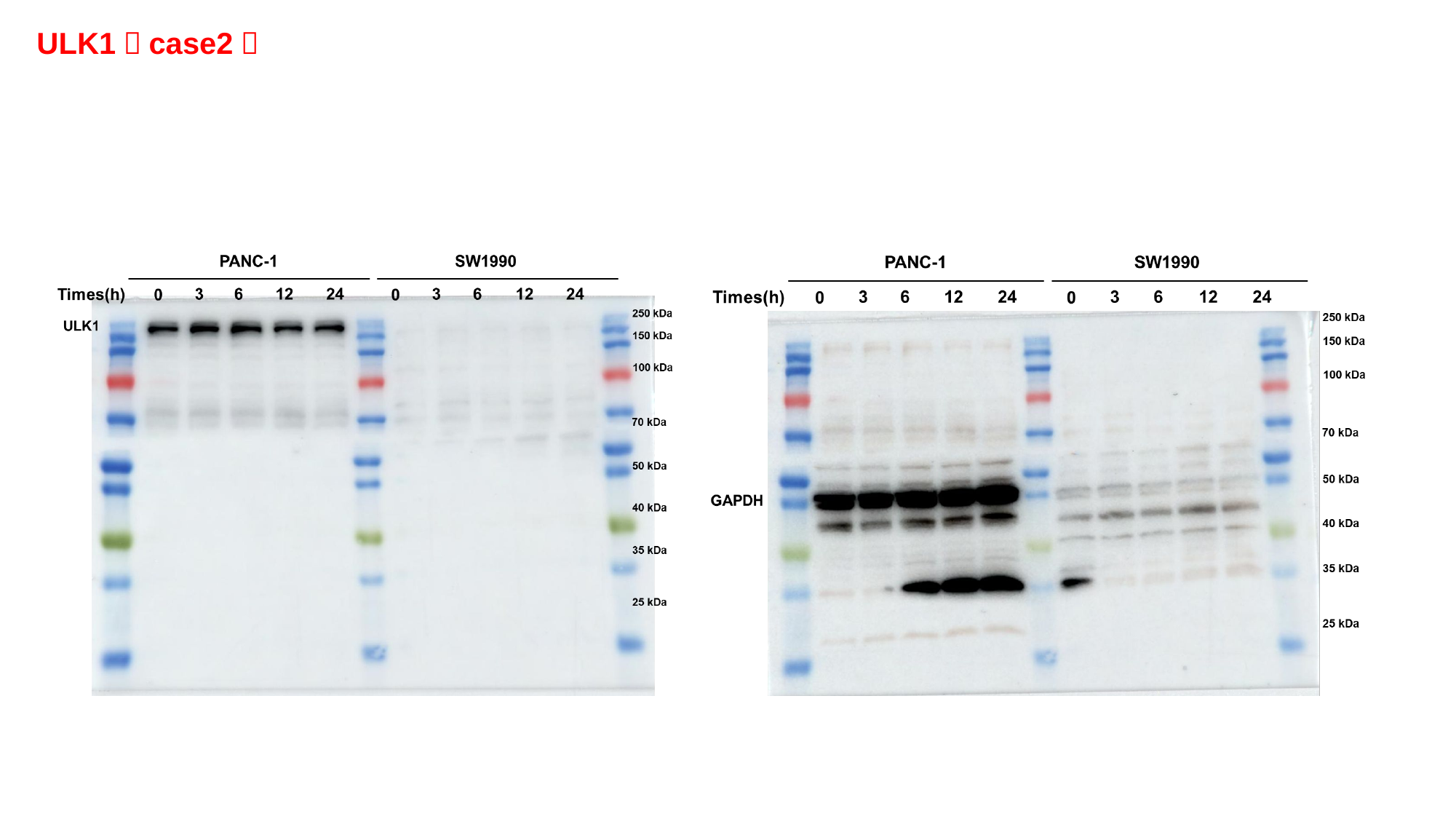

ULK1（case2）

## Slide 30
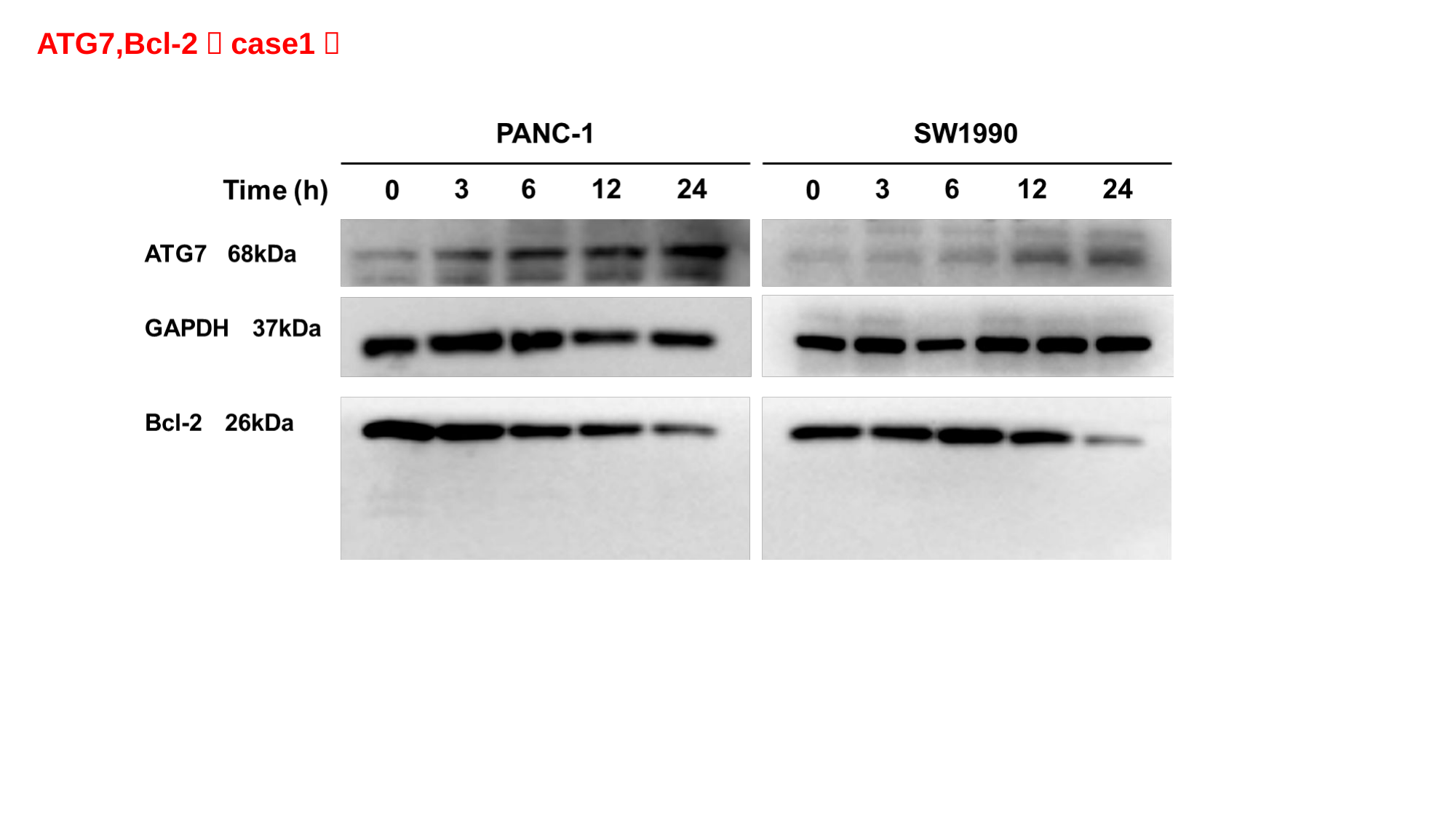

ATG7,Bcl-2（case1）

## Slide 31
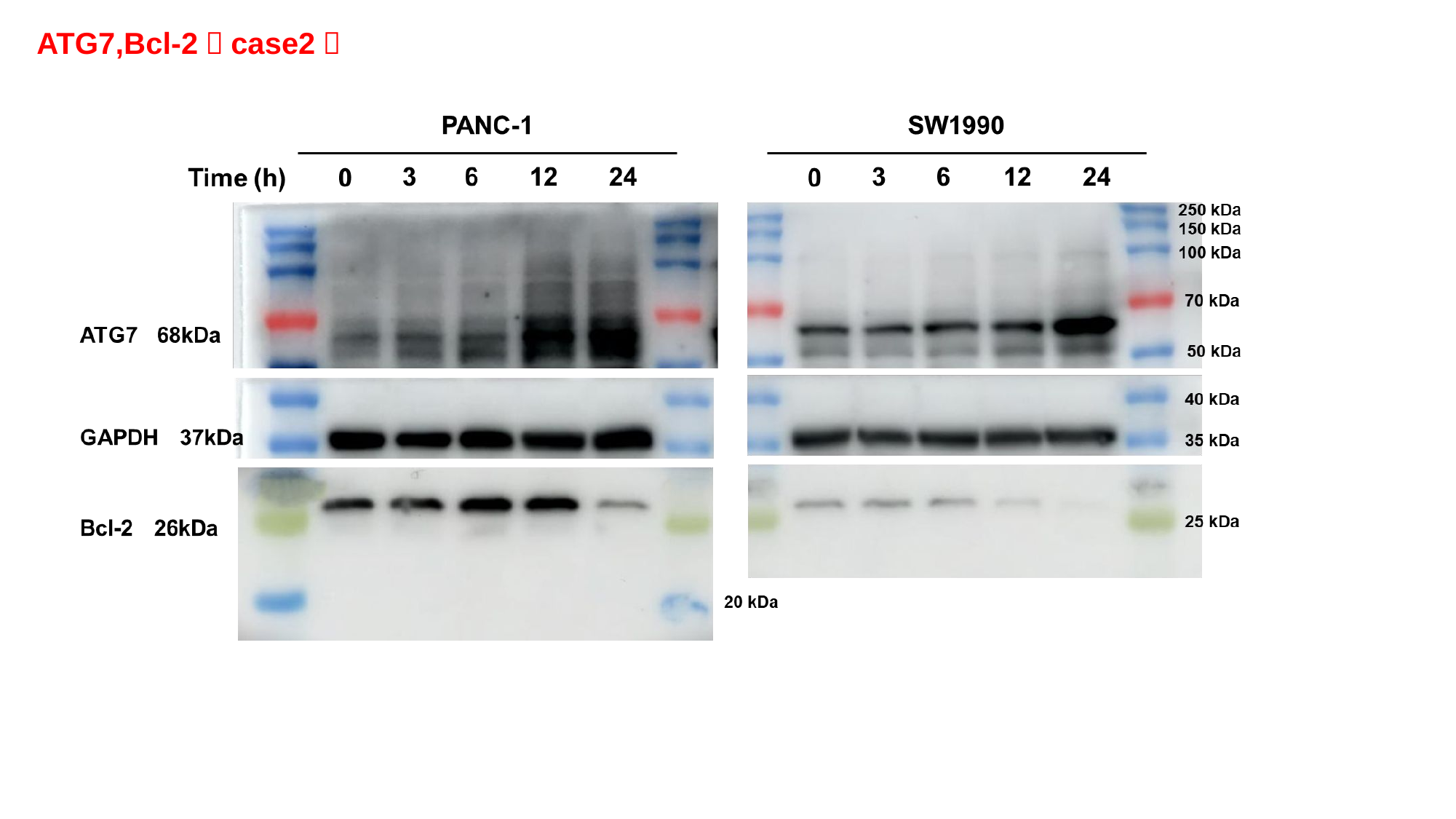

ATG7,Bcl-2（case2）

## Slide 32
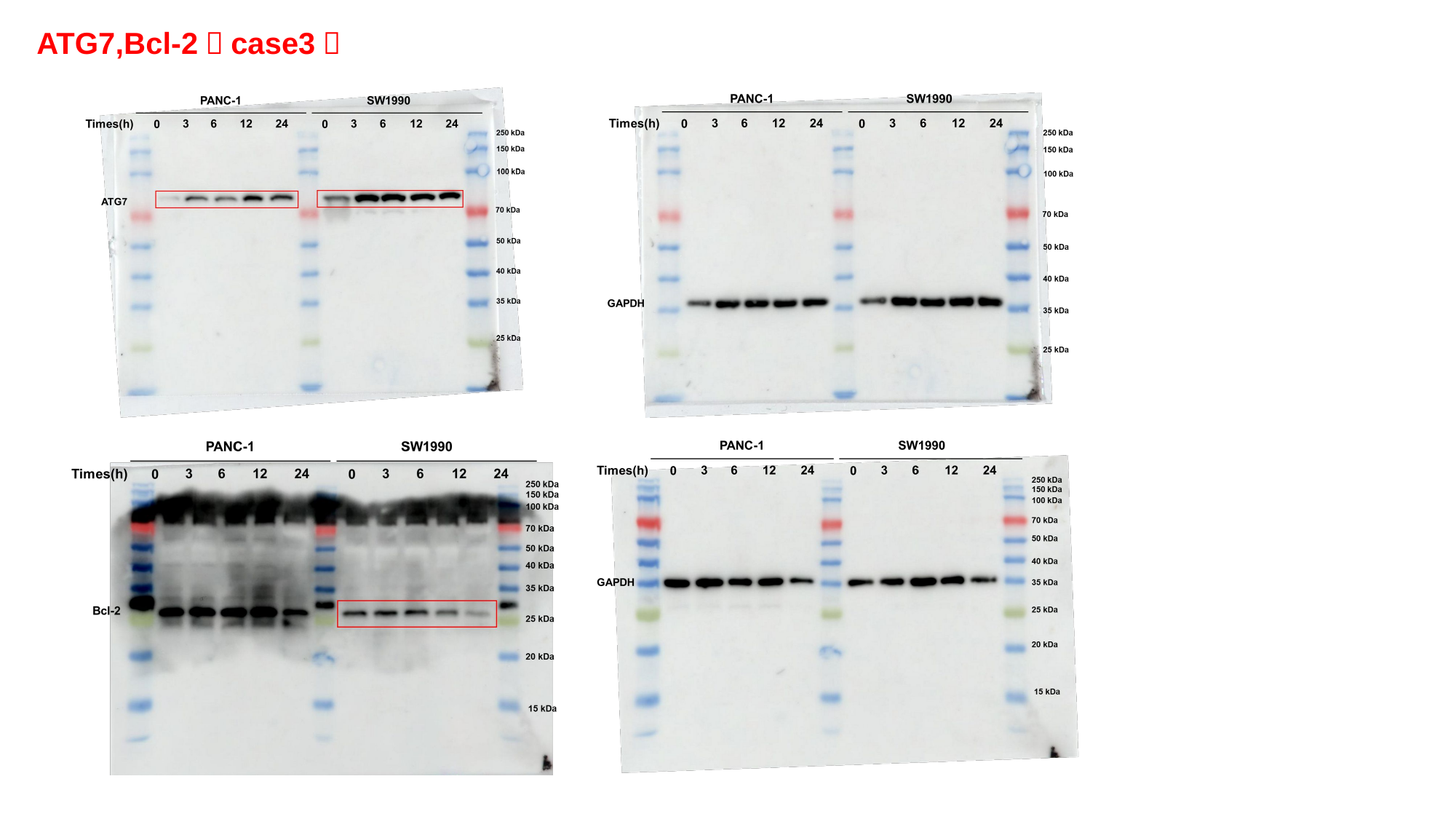

ATG7,Bcl-2（case3）

## Slide 33
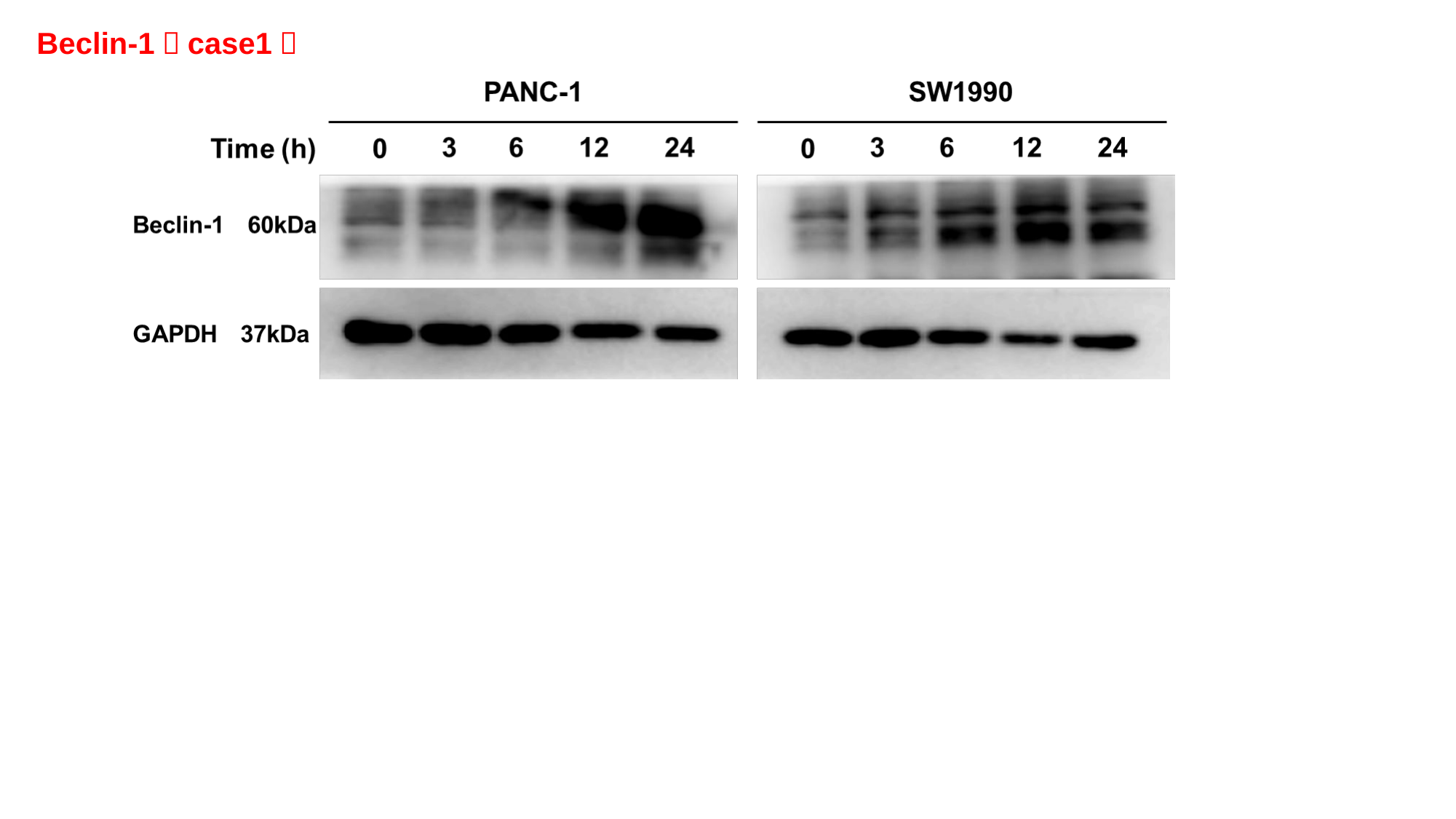

Beclin-1（case1）

## Slide 34
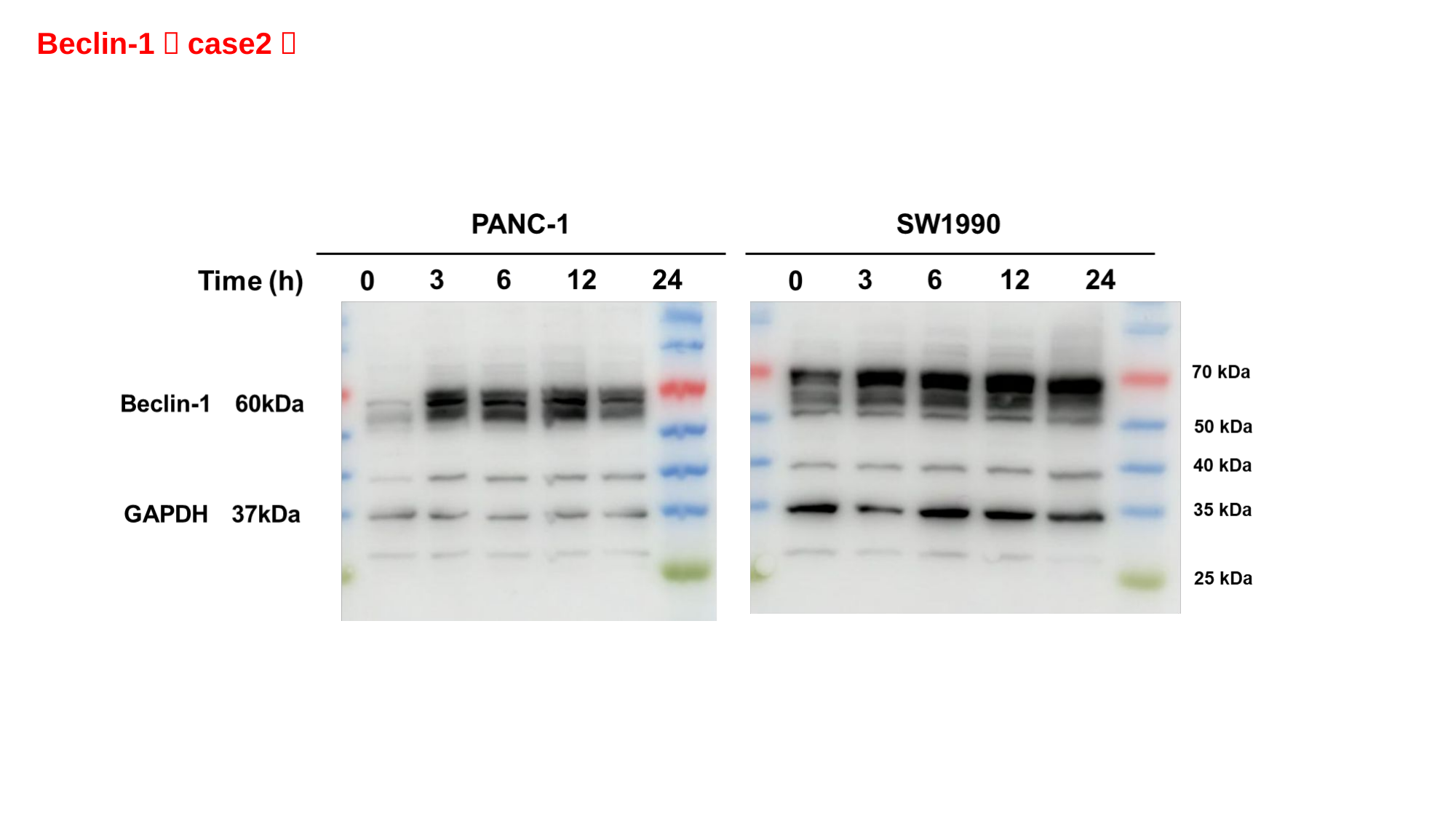

Beclin-1（case2）

## Slide 35
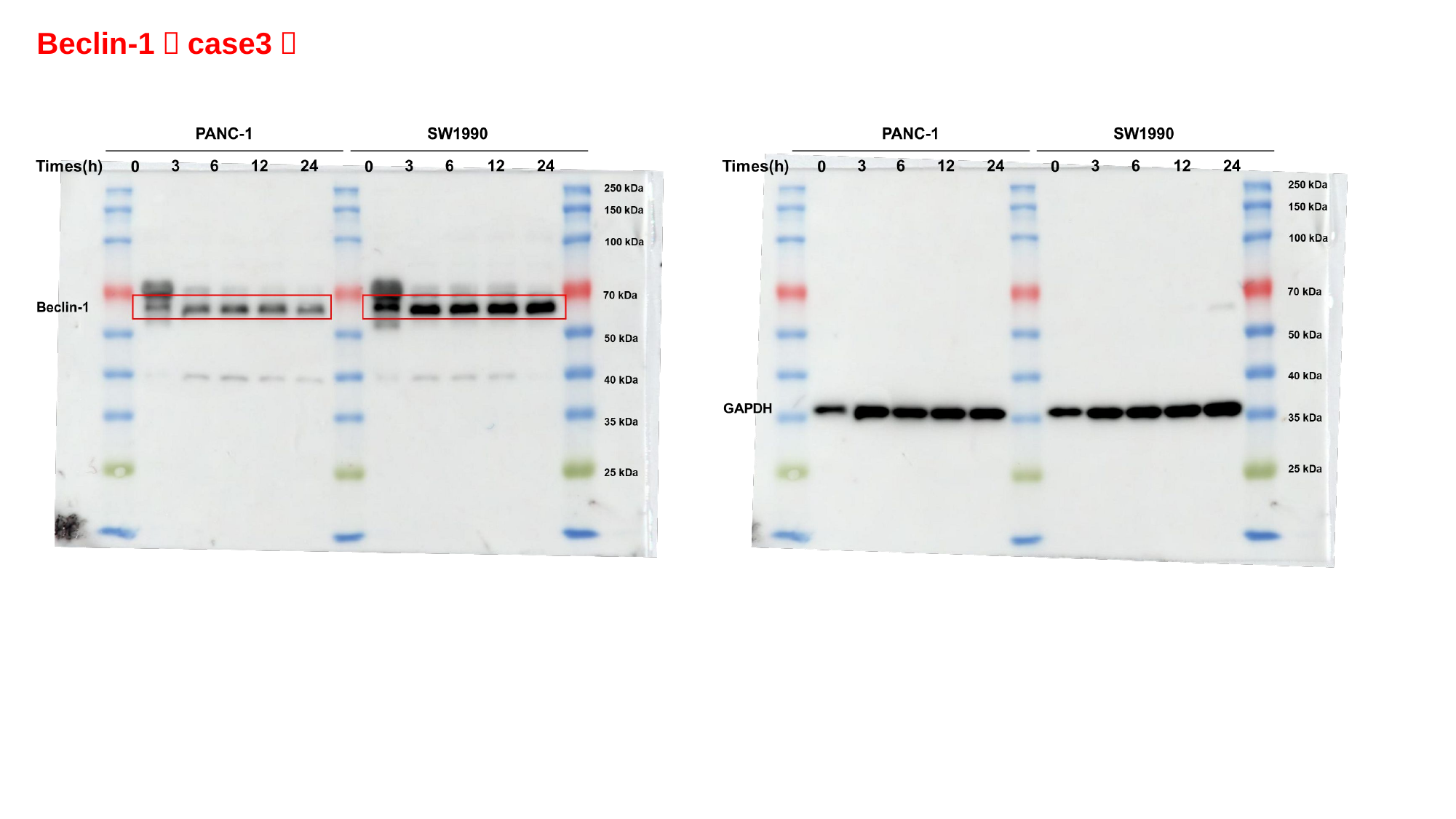

Beclin-1（case3）

## Slide 36
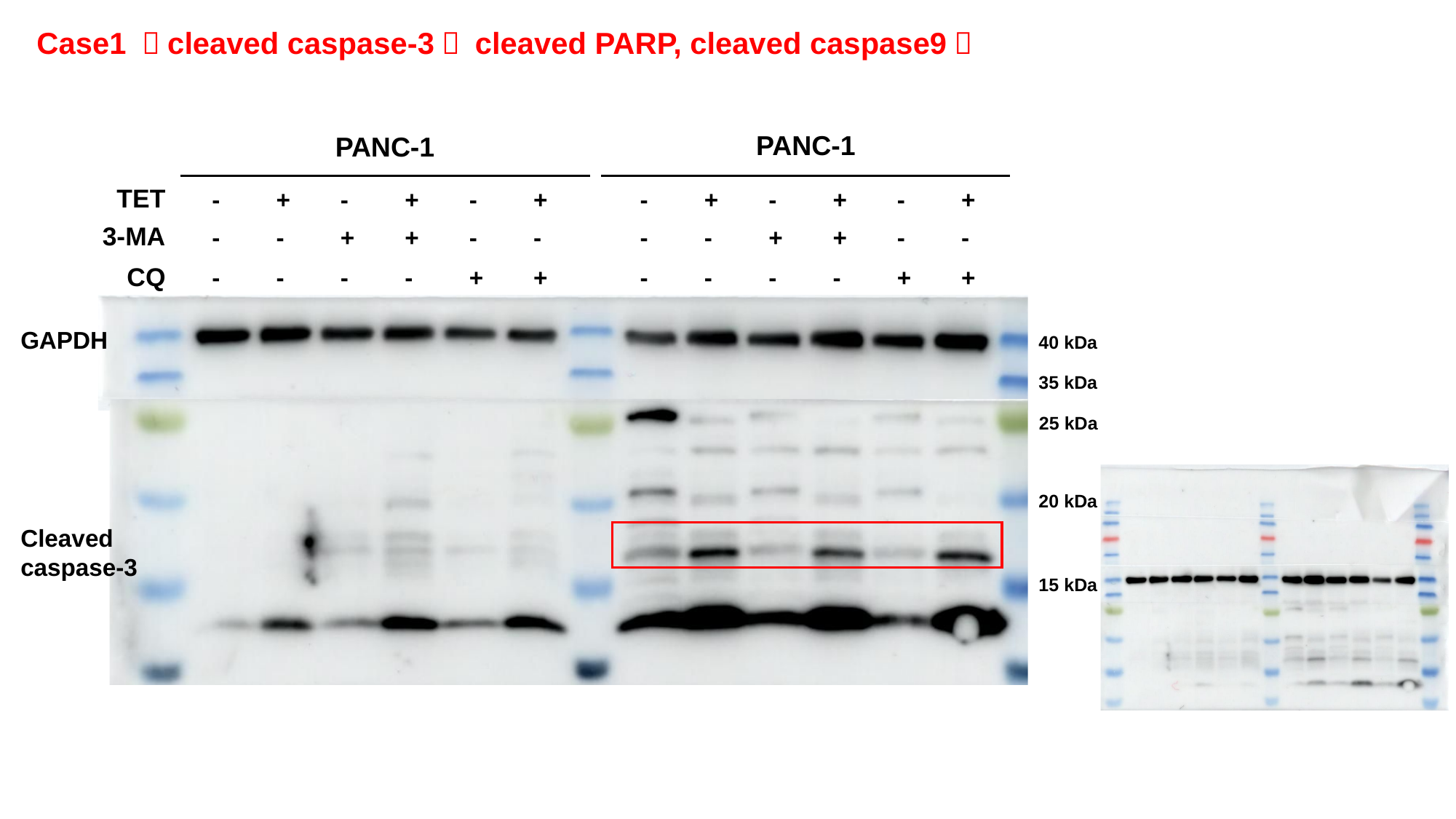

Case1 （cleaved caspase-3， cleaved PARP, cleaved caspase9）
PANC-1
PANC-1
TET
-
+
-
+
-
+
-
+
-
+
-
+
3-MA
-
-
+
+
-
-
-
-
+
+
-
-
CQ
-
-
-
-
+
+
-
-
-
-
+
+
GAPDH
40 kDa
35 kDa
25 kDa
20 kDa
Cleaved caspase-3
15 kDa

## Slide 37
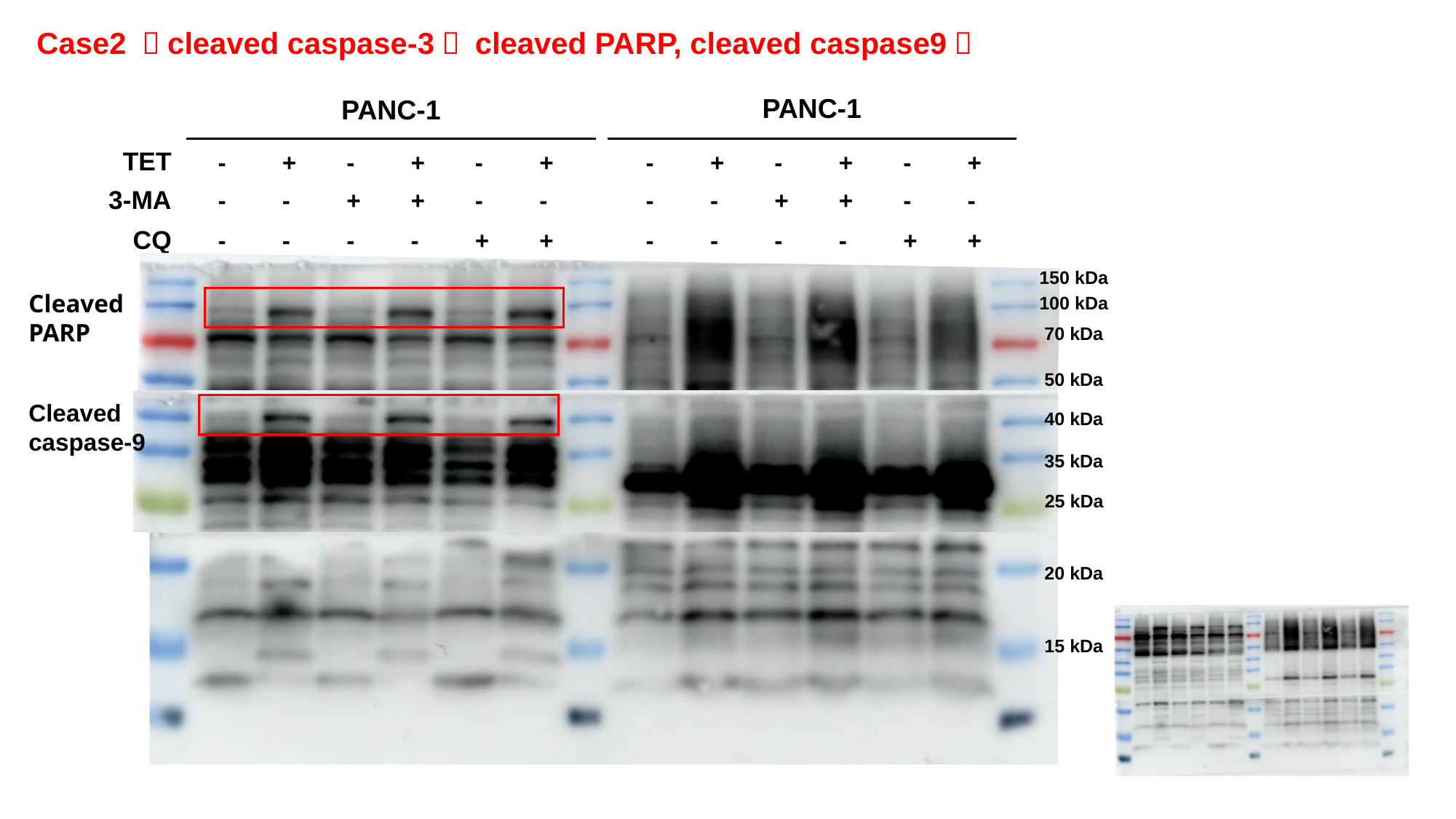

Case2 （cleaved caspase-3， cleaved PARP, cleaved caspase9）
PANC-1
PANC-1
TET
-
+
-
+
-
+
-
+
-
+
-
+
3-MA
-
-
+
+
-
-
-
-
+
+
-
-
CQ
-
-
-
-
+
+
-
-
-
-
+
+
150 kDa
Cleaved
PARP
100 kDa
70 kDa
50 kDa
Cleaved caspase-9
40 kDa
35 kDa
25 kDa
20 kDa
15 kDa

## Slide 38
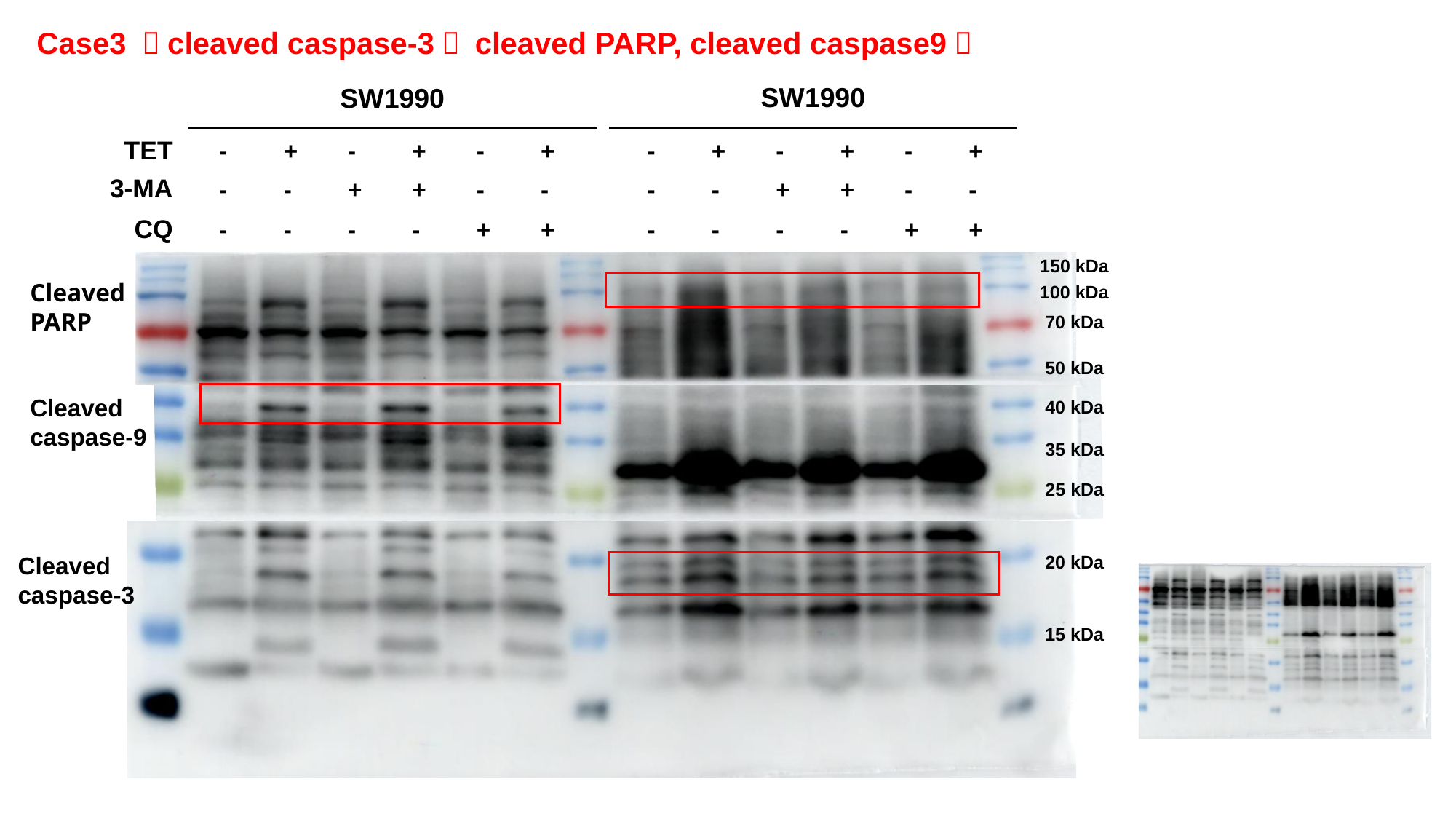

Case3 （cleaved caspase-3， cleaved PARP, cleaved caspase9）
SW1990
SW1990
TET
-
+
-
+
-
+
-
+
-
+
-
+
3-MA
-
-
+
+
-
-
-
-
+
+
-
-
CQ
-
-
-
-
+
+
-
-
-
-
+
+
150 kDa
Cleaved
PARP
100 kDa
70 kDa
50 kDa
Cleaved caspase-9
40 kDa
35 kDa
25 kDa
20 kDa
Cleaved caspase-3
15 kDa

## Slide 39
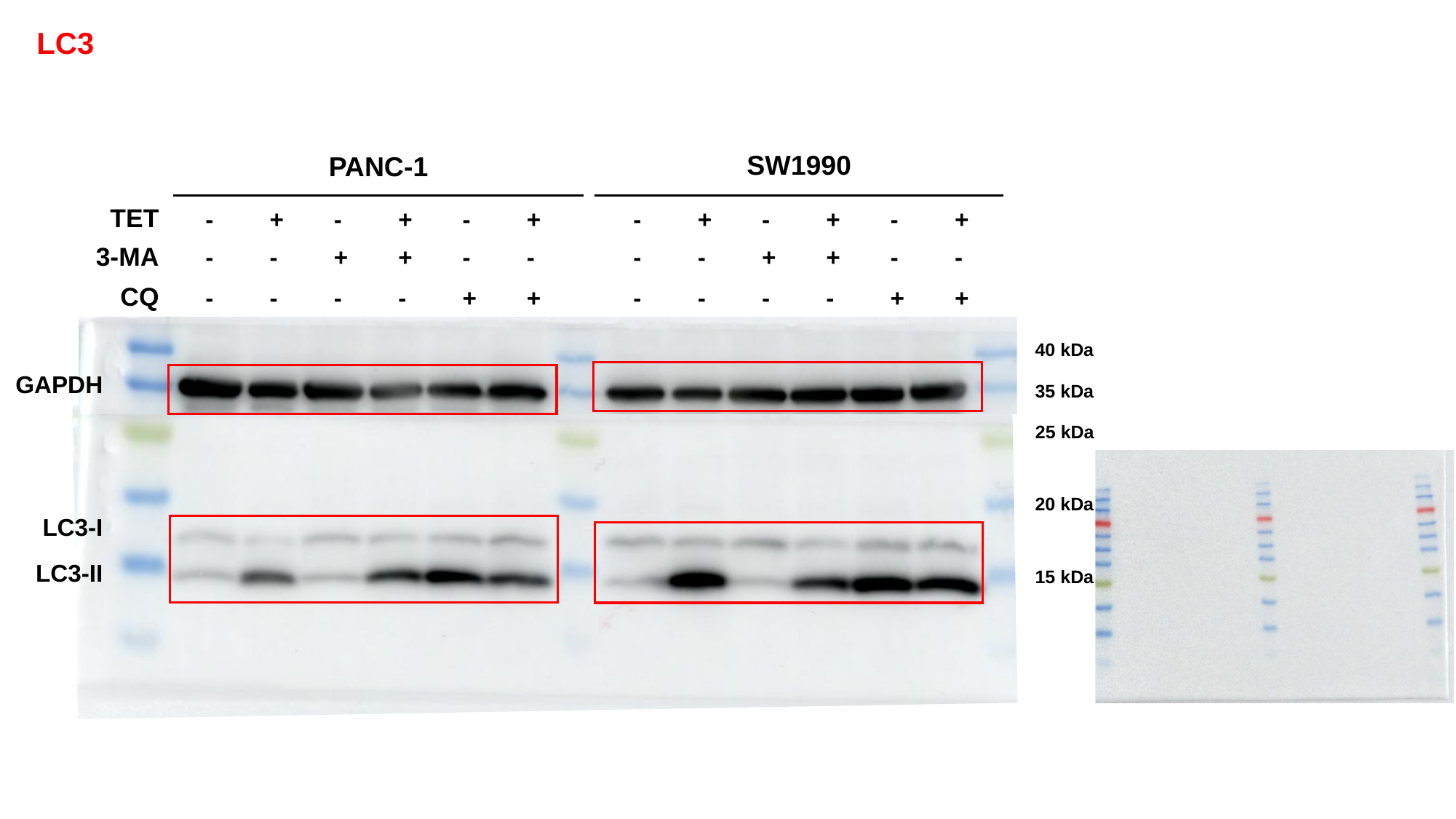

LC3
SW1990
PANC-1
TET
-
+
-
+
-
+
-
+
-
+
-
+
3-MA
-
-
+
+
-
-
-
-
+
+
-
-
CQ
-
-
-
-
+
+
-
-
-
-
+
+
40 kDa
GAPDH
35 kDa
25 kDa
20 kDa
LC3-I
LC3-II
15 kDa

## Slide 40
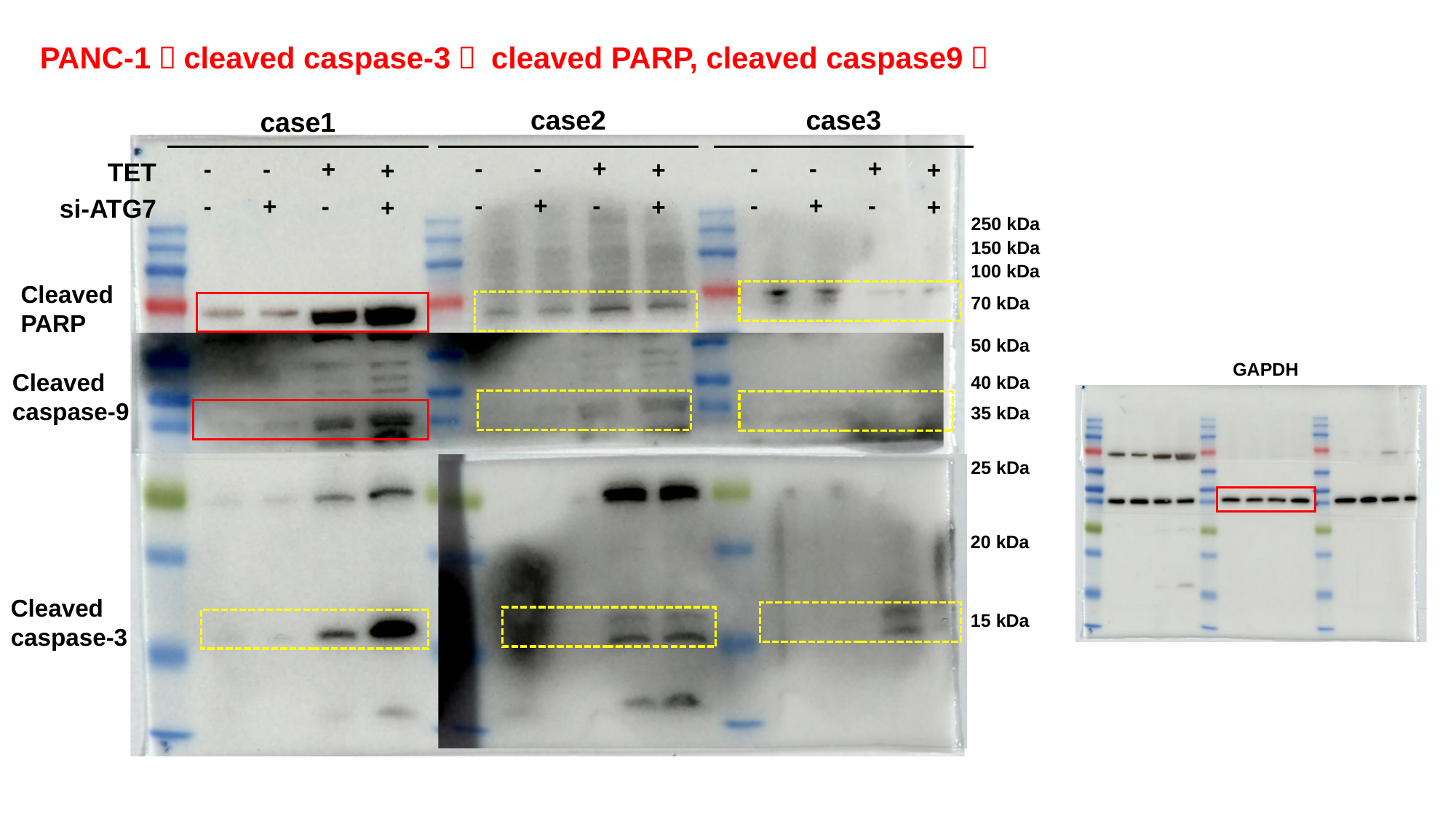

PANC-1（cleaved caspase-3， cleaved PARP, cleaved caspase9）
case2
case3
case1
-
-
+
-
-
+
-
-
+
+
+
+
TET
-
+
-
-
+
-
-
+
-
+
+
+
si-ATG7
250 kDa
150 kDa
100 kDa
Cleaved
PARP
70 kDa
50 kDa
Cleaved caspase-9
40 kDa
35 kDa
25 kDa
20 kDa
Cleaved caspase-3
15 kDa
GAPDH

## Slide 41
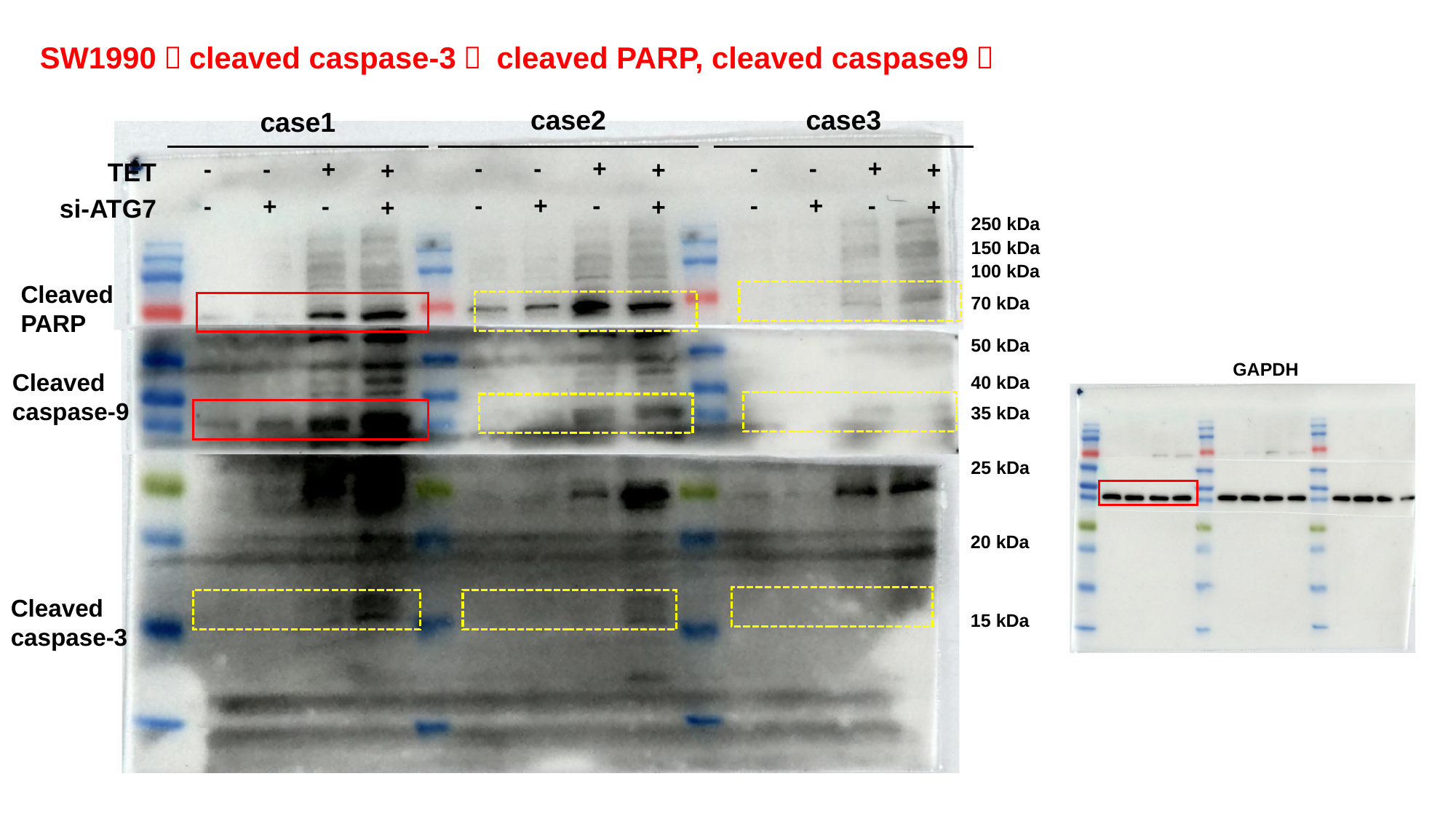

SW1990（cleaved caspase-3， cleaved PARP, cleaved caspase9）
case2
case3
case1
-
-
+
-
-
+
-
-
+
+
+
+
TET
-
+
-
-
+
-
-
+
-
+
+
+
si-ATG7
250 kDa
150 kDa
100 kDa
Cleaved
PARP
70 kDa
50 kDa
GAPDH
Cleaved caspase-9
40 kDa
35 kDa
25 kDa
20 kDa
Cleaved caspase-3
15 kDa

## Slide 42
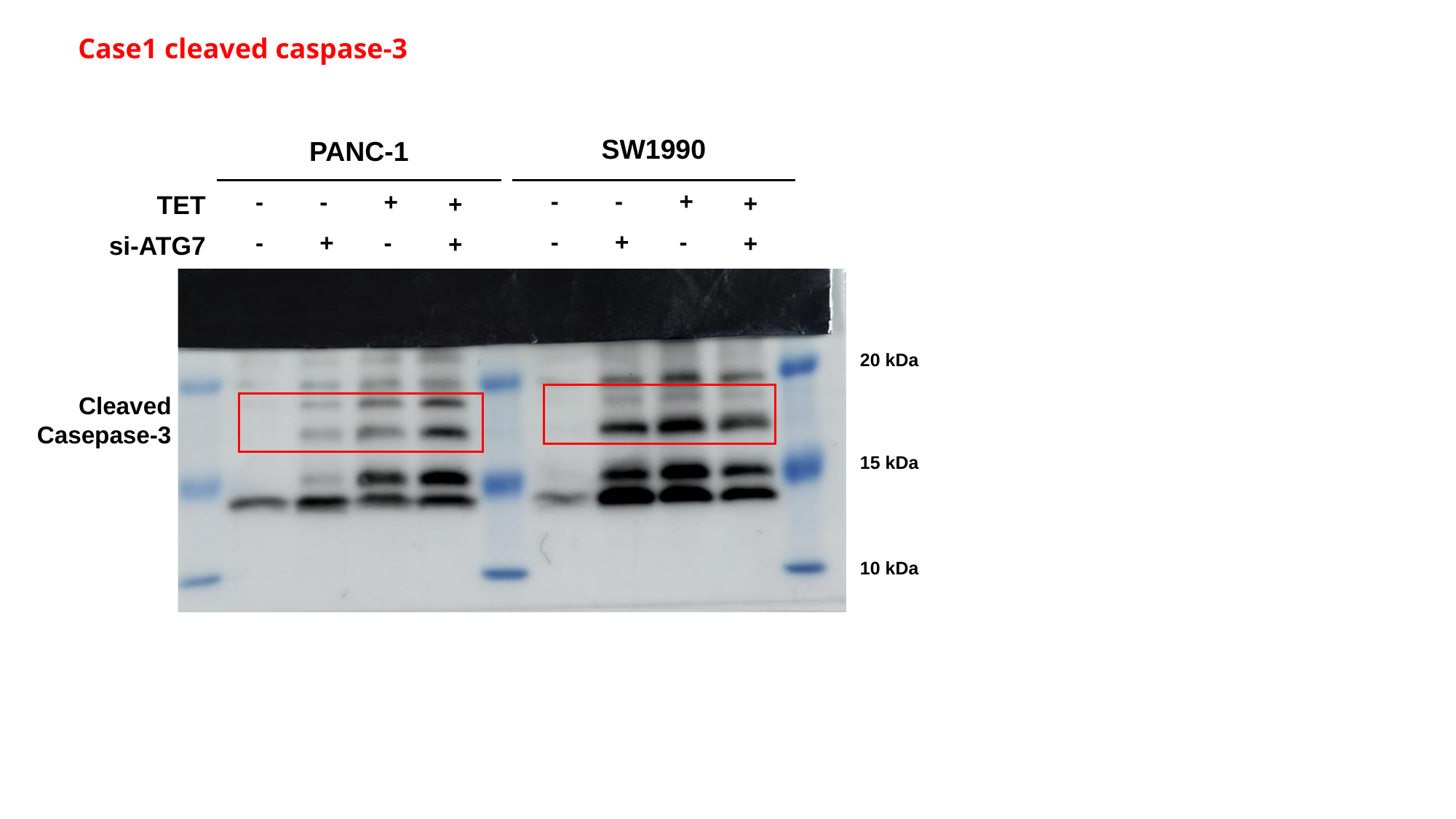

Case1 cleaved caspase-3
SW1990
PANC-1
-
-
+
-
-
+
+
+
TET
-
+
-
-
+
-
+
+
si-ATG7
20 kDa
Cleaved
Casepase-3
15 kDa
10 kDa

## Slide 43
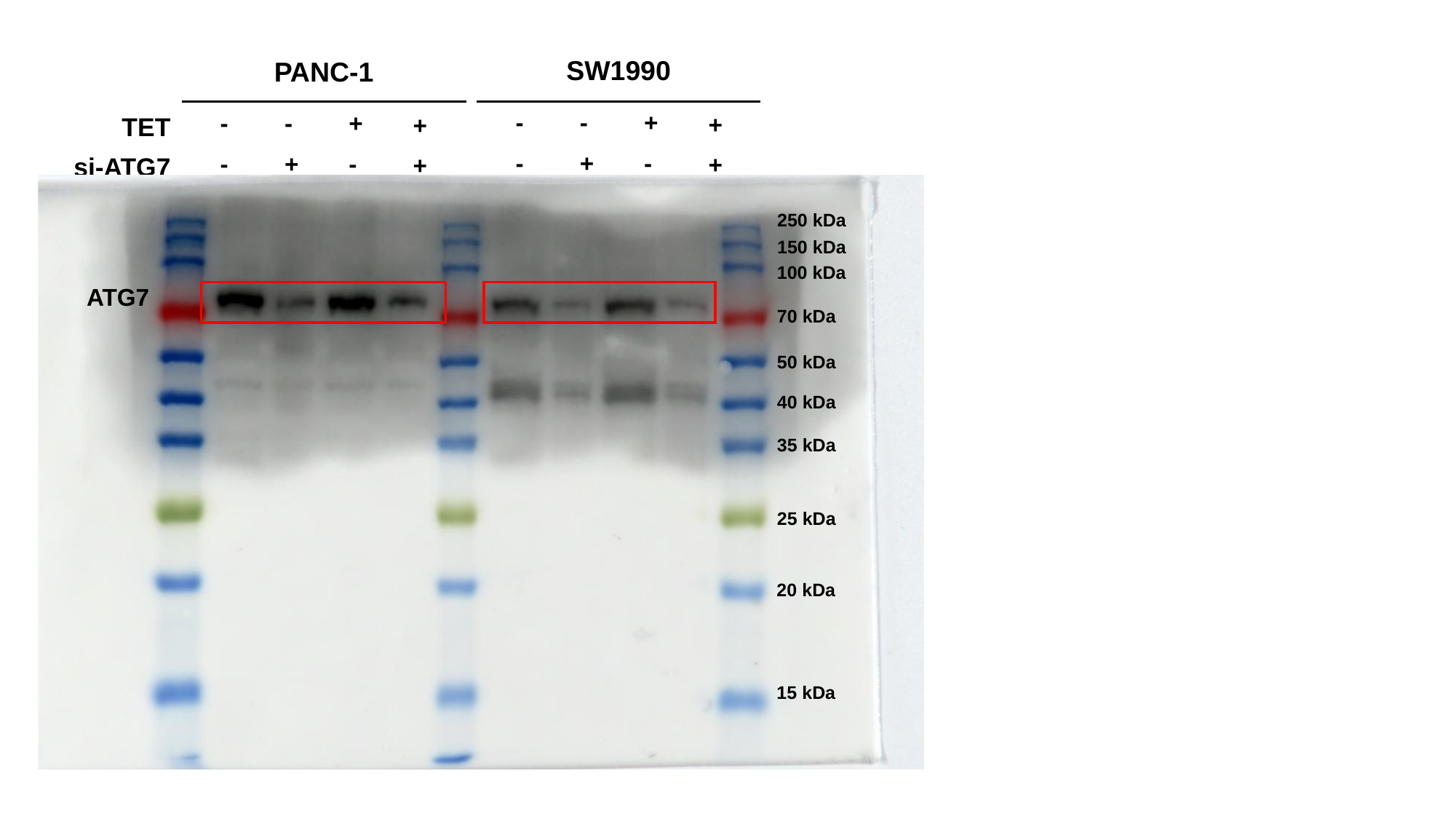

SW1990
PANC-1
-
-
+
-
-
+
+
+
TET
-
+
-
-
+
-
+
+
si-ATG7
250 kDa
150 kDa
100 kDa
ATG7
70 kDa
50 kDa
40 kDa
35 kDa
25 kDa
20 kDa
15 kDa

## Slide 44
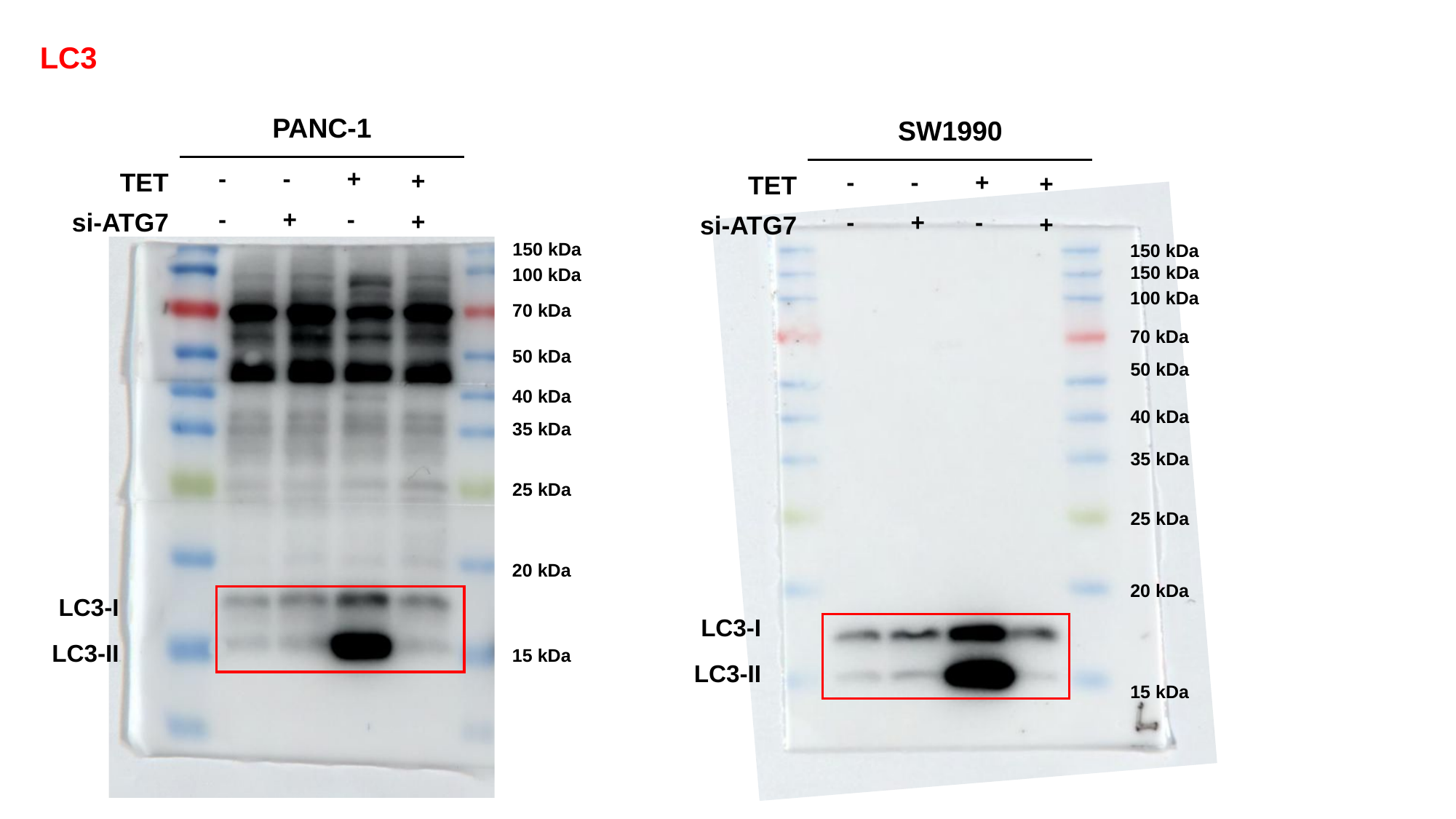

LC3
PANC-1
SW1990
-
-
+
+
TET
-
-
+
+
TET
-
+
-
+
si-ATG7
-
+
-
+
si-ATG7
150 kDa
150 kDa
150 kDa
100 kDa
100 kDa
70 kDa
70 kDa
50 kDa
50 kDa
40 kDa
40 kDa
35 kDa
35 kDa
25 kDa
25 kDa
20 kDa
20 kDa
LC3-I
LC3-I
LC3-II
15 kDa
LC3-II
15 kDa

## Slide 45
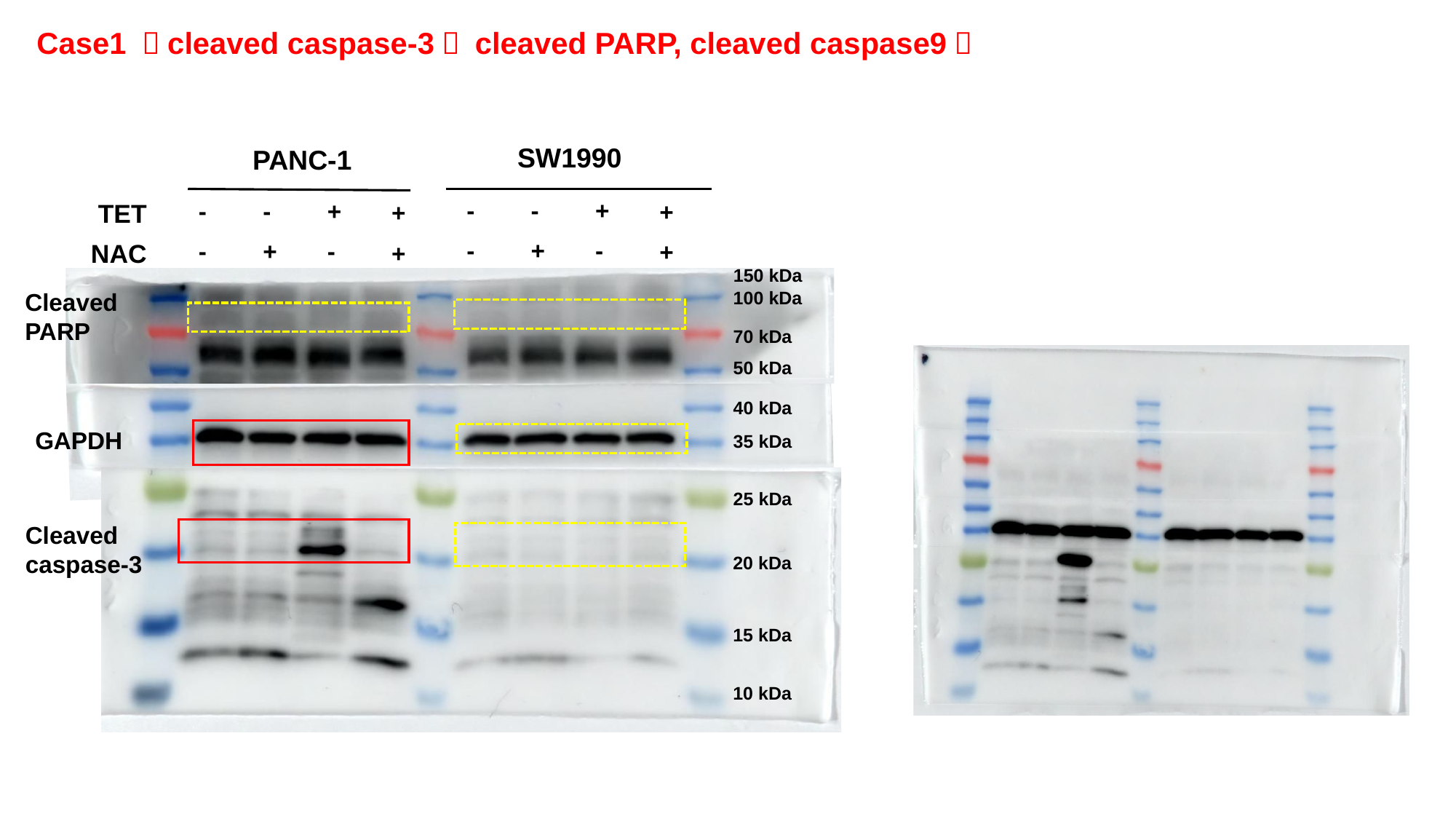

Case1 （cleaved caspase-3， cleaved PARP, cleaved caspase9）
SW1990
PANC-1
-
-
+
-
-
+
+
TET
+
-
+
-
-
+
-
+
NAC
+
150 kDa
100 kDa
Cleaved
PARP
70 kDa
50 kDa
40 kDa
GAPDH
35 kDa
25 kDa
Cleaved caspase-3
20 kDa
15 kDa
10 kDa

## Slide 46
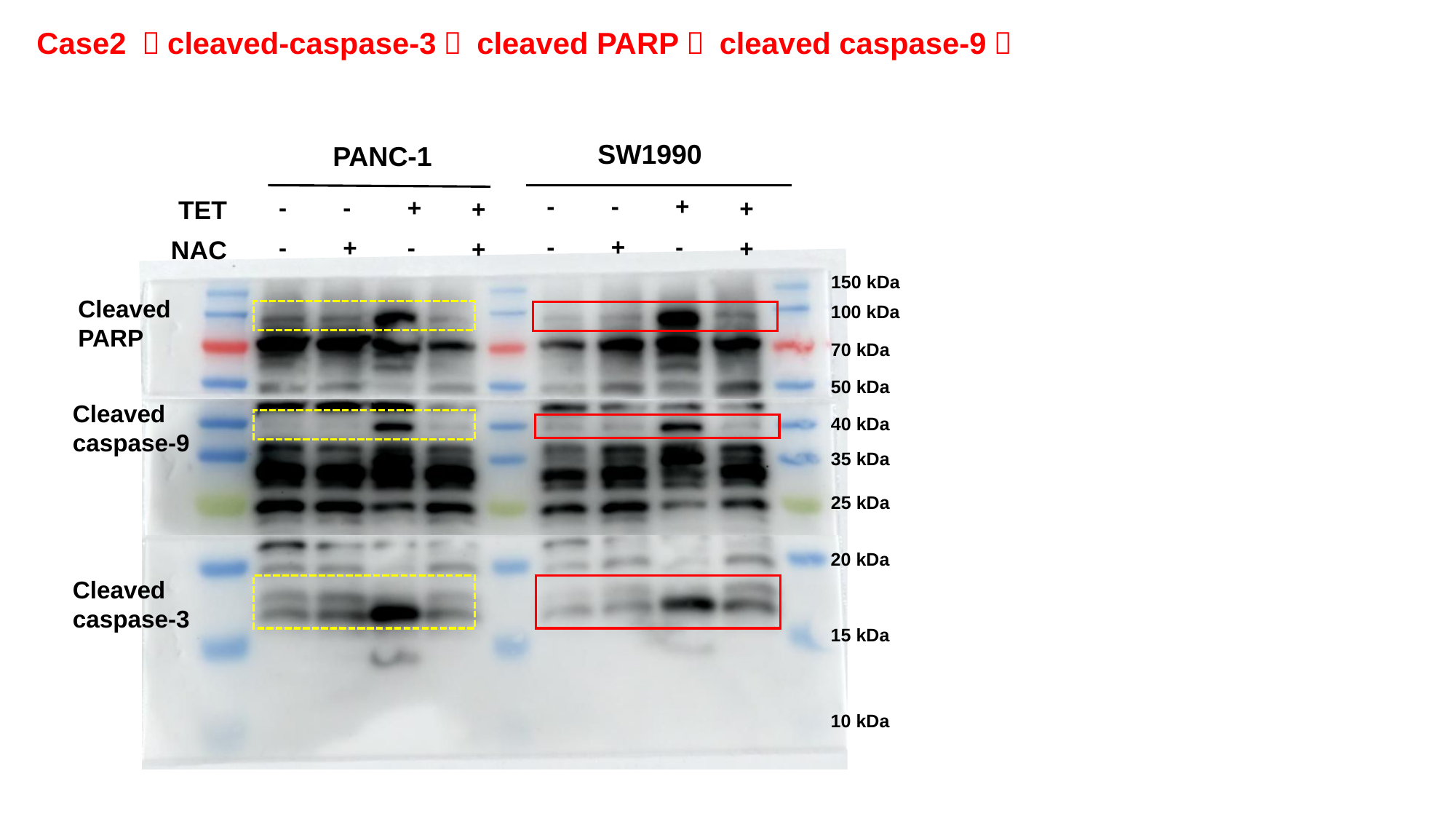

Case2 （cleaved-caspase-3， cleaved PARP， cleaved caspase-9）
SW1990
PANC-1
-
-
+
-
-
+
+
TET
+
-
+
-
-
+
-
+
NAC
+
150 kDa
Cleaved
PARP
100 kDa
70 kDa
50 kDa
Cleaved caspase-9
40 kDa
35 kDa
25 kDa
20 kDa
Cleaved caspase-3
15 kDa
10 kDa

## Slide 47
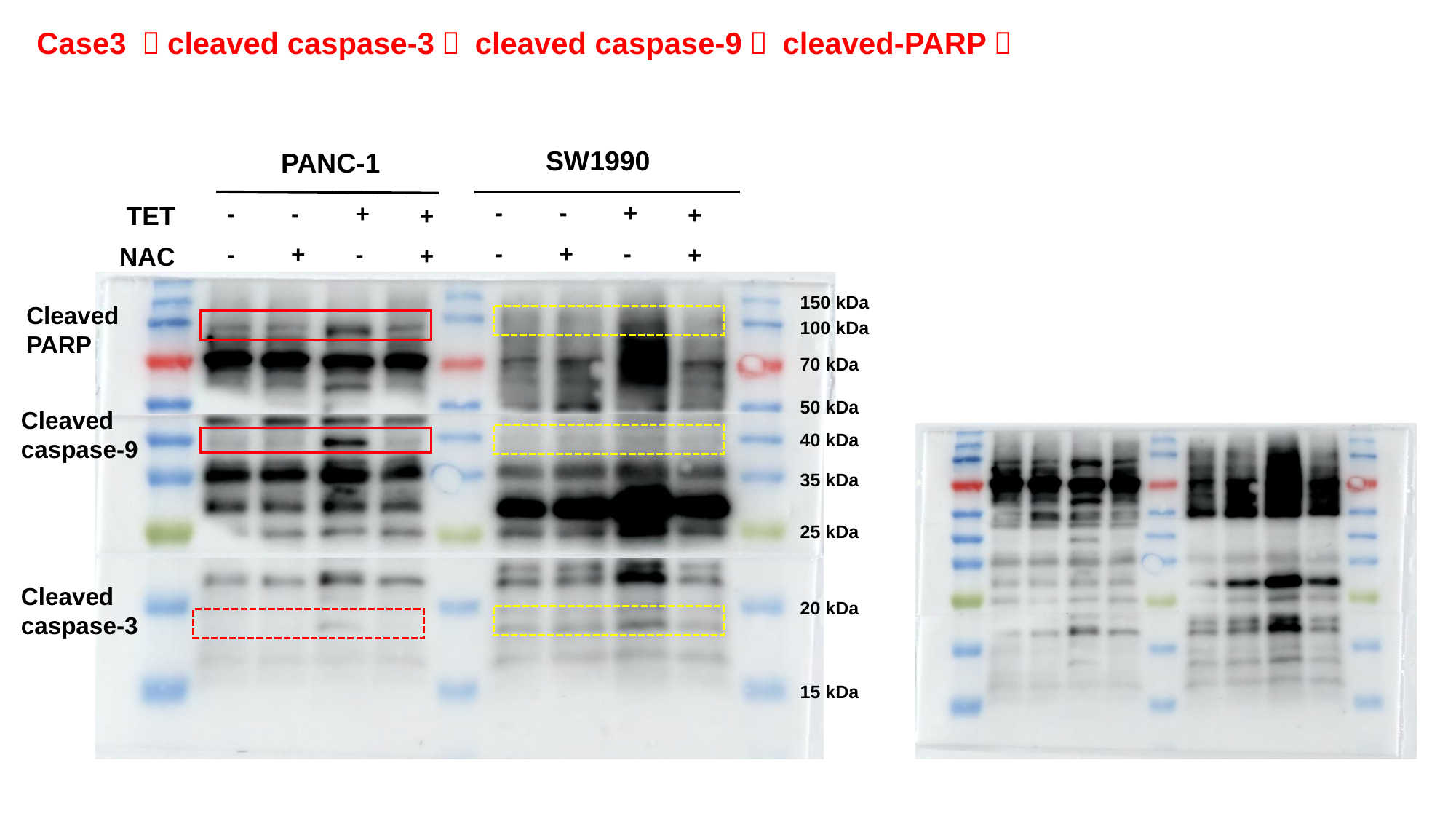

Case3 （cleaved caspase-3， cleaved caspase-9， cleaved-PARP）
SW1990
PANC-1
-
-
+
-
-
+
+
TET
+
-
+
-
-
+
-
+
NAC
+
150 kDa
Cleaved
PARP
100 kDa
70 kDa
50 kDa
Cleaved caspase-9
40 kDa
35 kDa
25 kDa
Cleaved caspase-3
20 kDa
15 kDa

## Slide 48
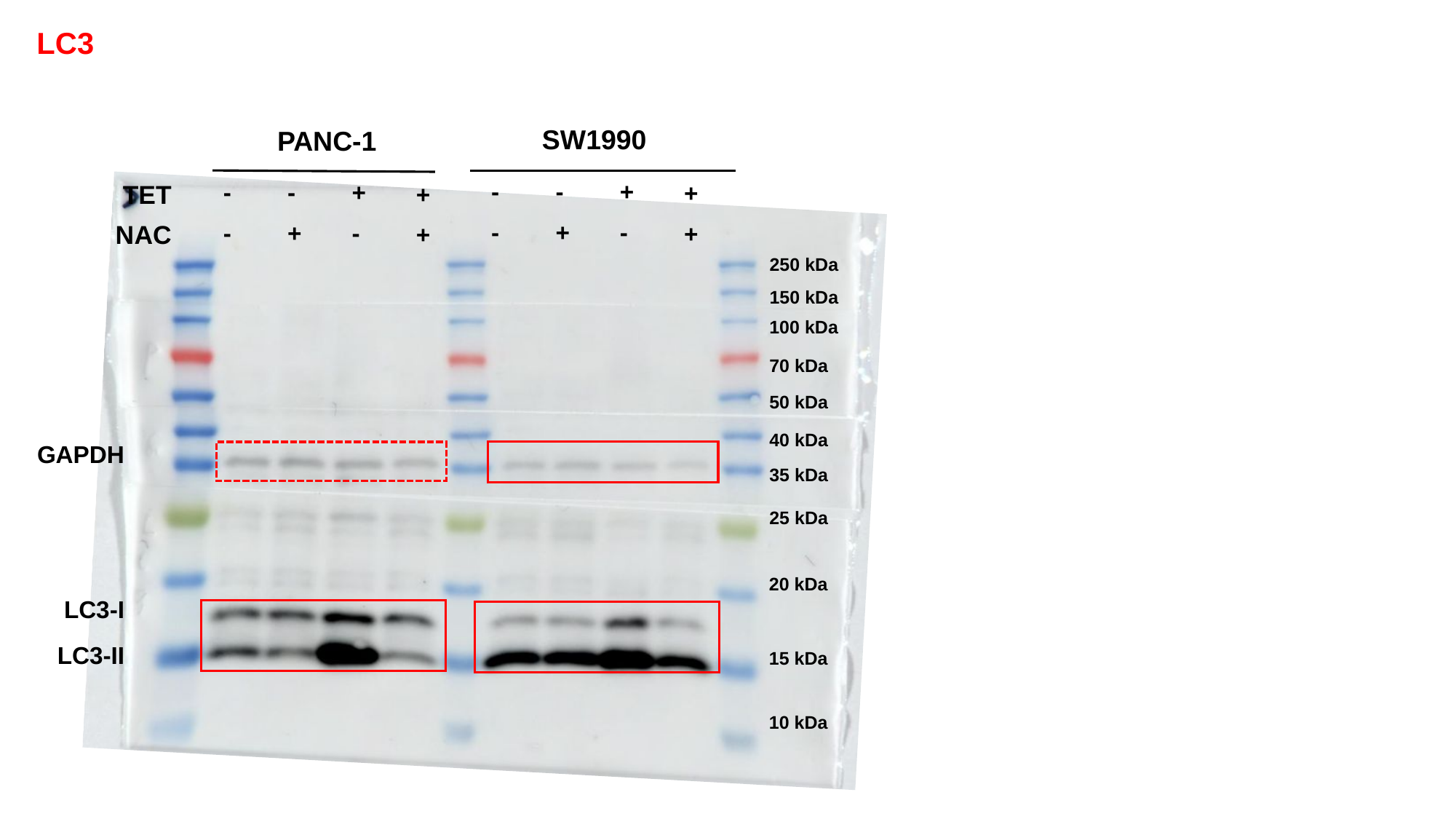

LC3
SW1990
PANC-1
-
-
+
-
-
+
+
TET
+
-
+
-
-
+
-
+
NAC
+
250 kDa
150 kDa
100 kDa
70 kDa
50 kDa
40 kDa
GAPDH
35 kDa
25 kDa
20 kDa
LC3-I
LC3-II
15 kDa
10 kDa

## Slide 49
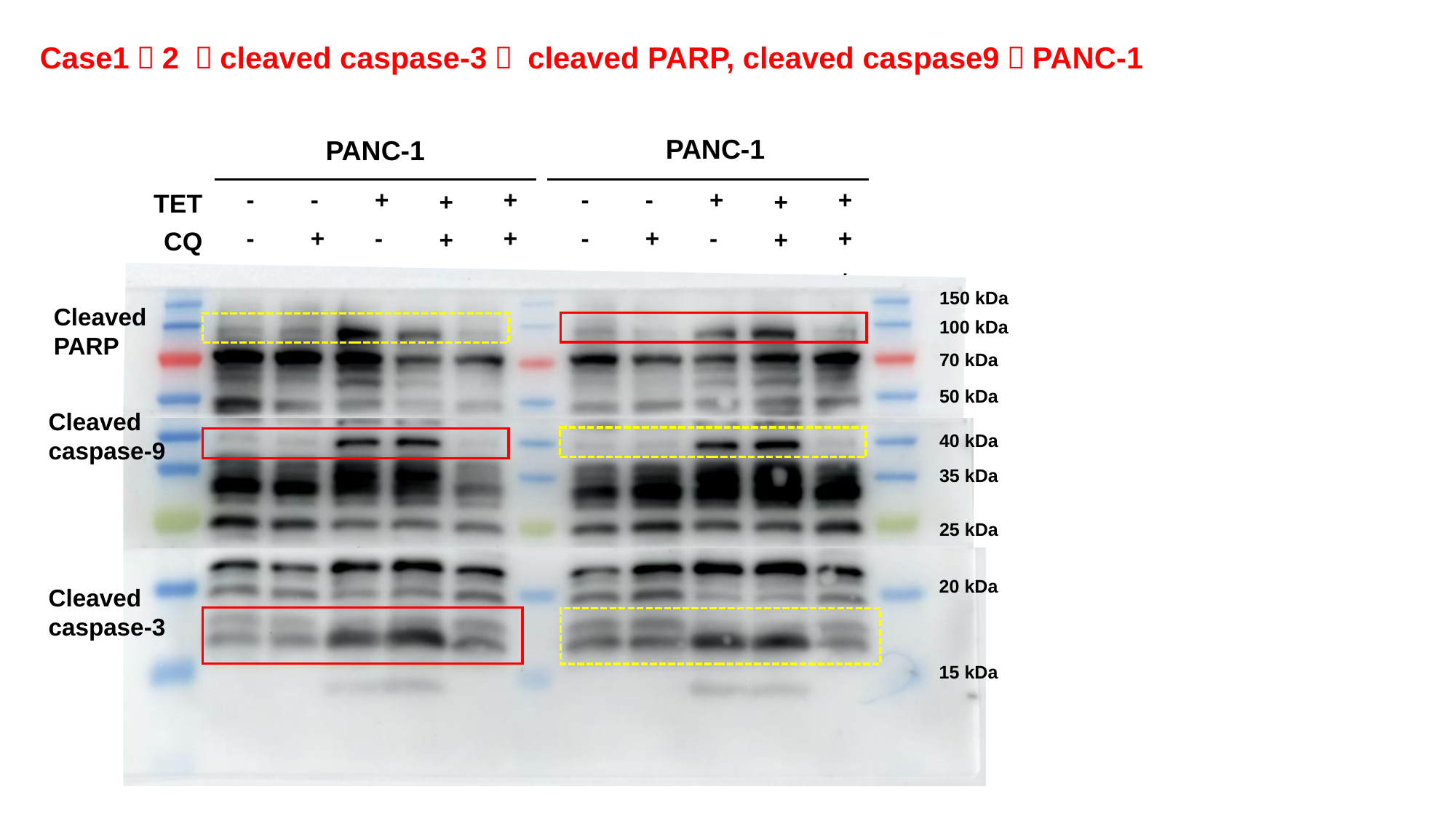

Case1，2 （cleaved caspase-3， cleaved PARP, cleaved caspase9）PANC-1
PANC-1
PANC-1
-
-
+
+
-
-
+
+
+
+
TET
-
+
-
+
-
+
-
+
+
+
CQ
-
-
-
+
-
-
-
+
-
-
NAC
150 kDa
Cleaved
PARP
100 kDa
70 kDa
50 kDa
Cleaved caspase-9
40 kDa
35 kDa
25 kDa
20 kDa
Cleaved caspase-3
15 kDa

## Slide 50
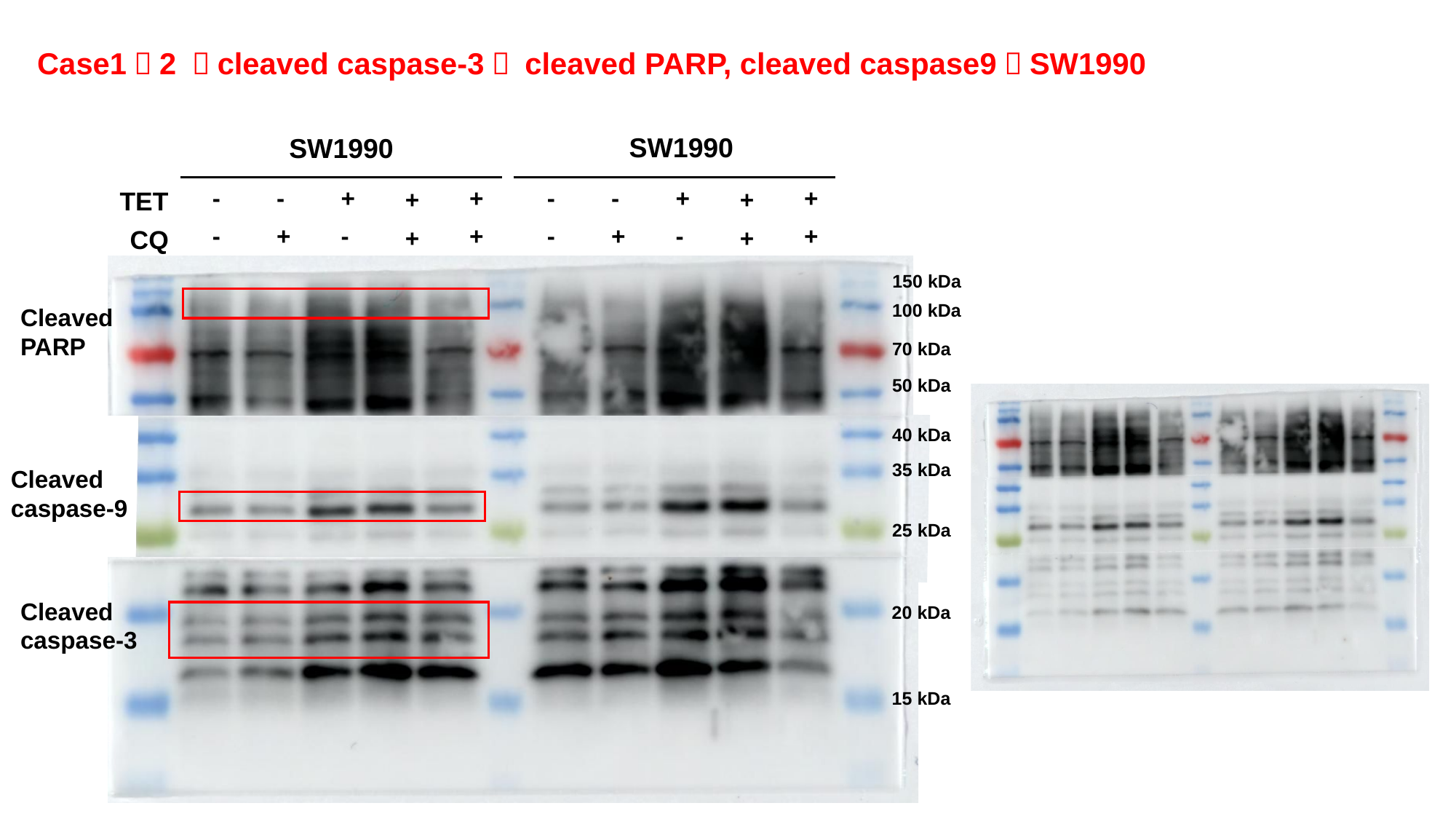

Case1，2 （cleaved caspase-3， cleaved PARP, cleaved caspase9）SW1990
SW1990
SW1990
-
-
+
+
-
-
+
+
+
+
TET
-
+
-
+
-
+
-
+
+
+
CQ
150 kDa
100 kDa
Cleaved
PARP
70 kDa
50 kDa
40 kDa
35 kDa
Cleaved caspase-9
25 kDa
Cleaved caspase-3
20 kDa
15 kDa

## Slide 51
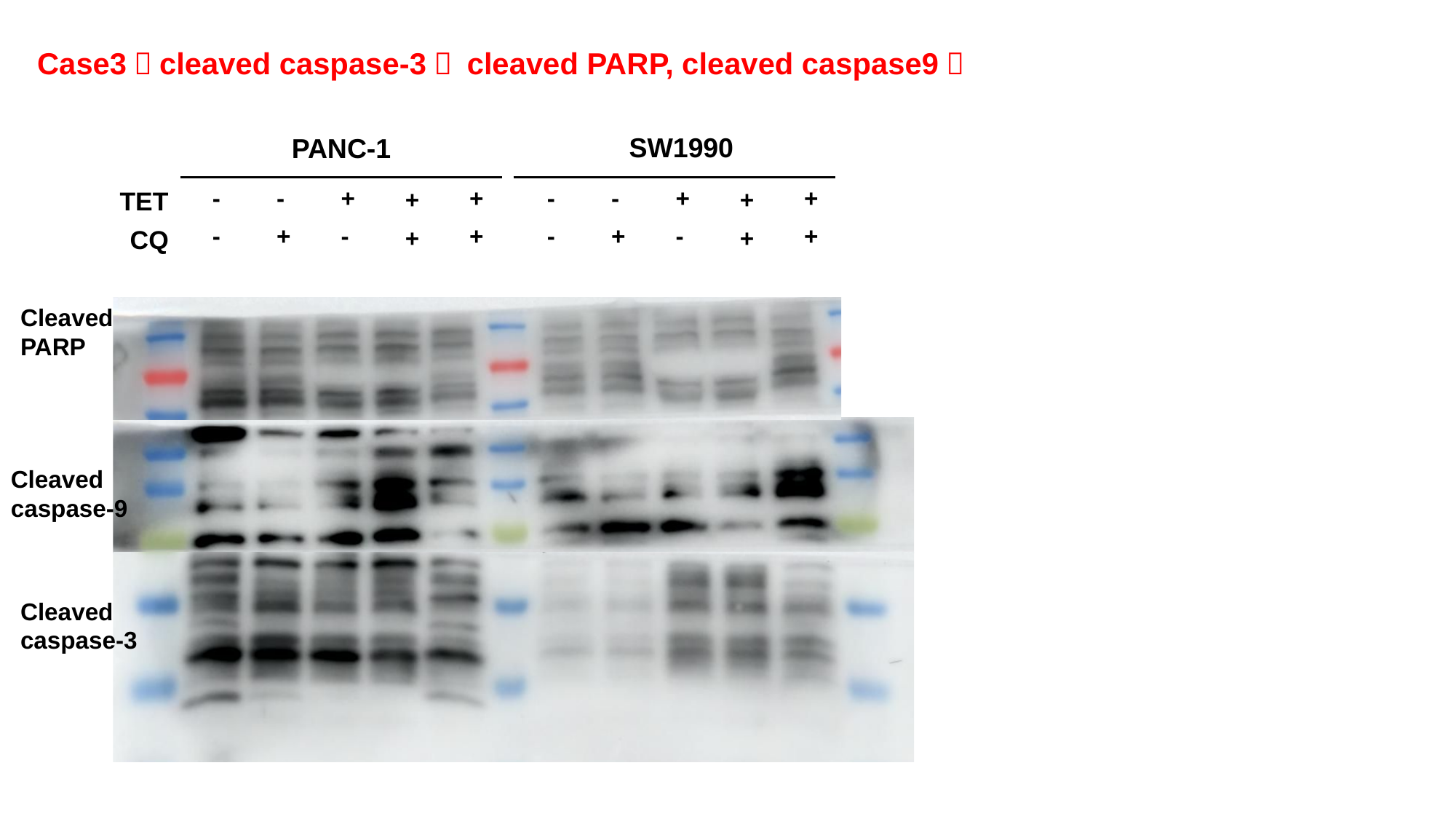

Case3（cleaved caspase-3， cleaved PARP, cleaved caspase9）
SW1990
PANC-1
-
-
+
+
-
-
+
+
+
+
TET
-
+
-
+
-
+
-
+
+
+
CQ
Cleaved
PARP
Cleaved caspase-9
Cleaved caspase-3

## Slide 52
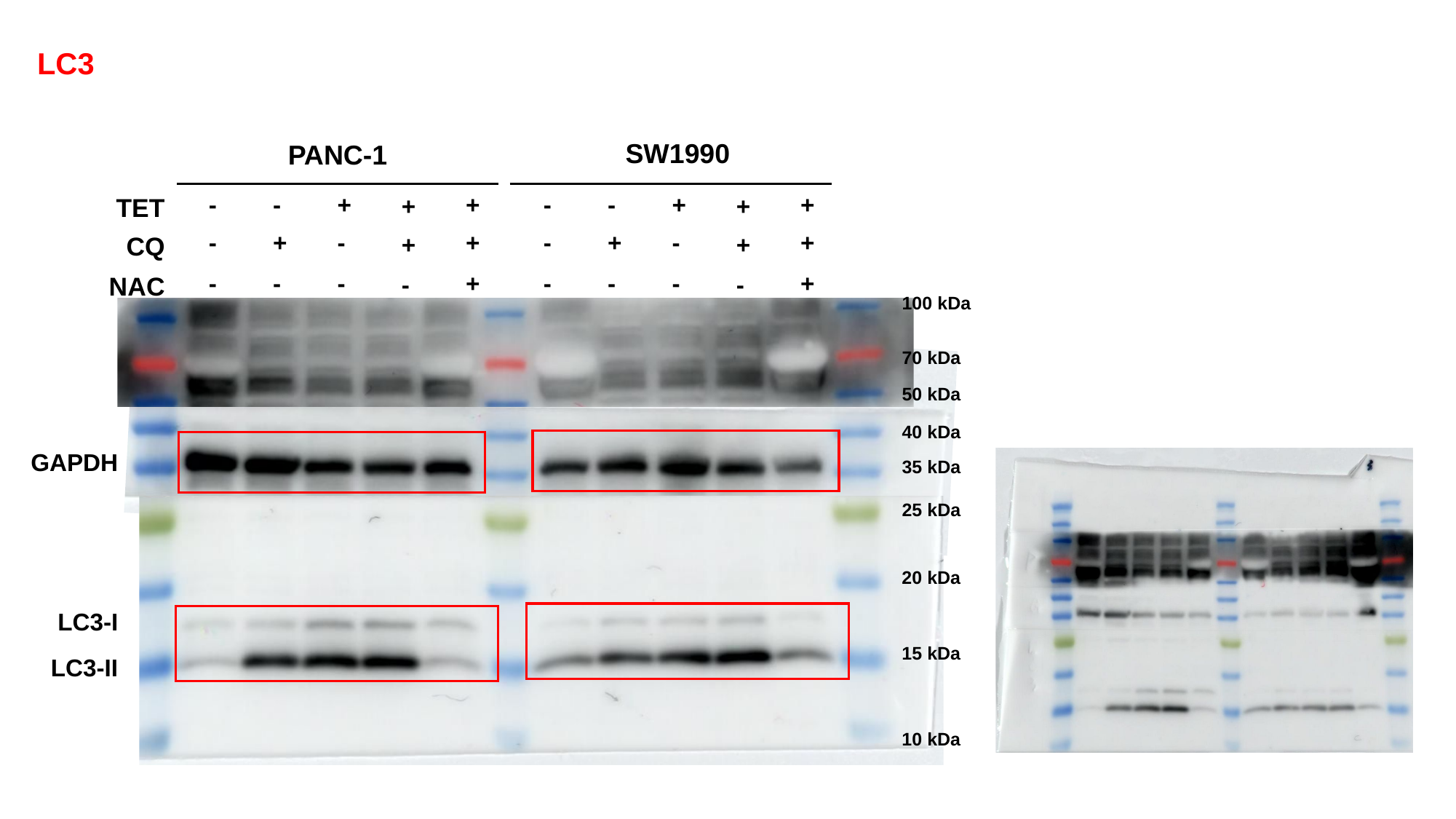

LC3
SW1990
PANC-1
-
-
+
+
-
-
+
+
+
+
TET
-
+
-
+
-
+
-
+
+
+
CQ
-
-
-
+
-
-
-
+
-
-
NAC
100 kDa
70 kDa
50 kDa
40 kDa
GAPDH
35 kDa
25 kDa
20 kDa
LC3-I
15 kDa
LC3-II
10 kDa
